# Supplementary figures and images for: Cell size reduction scales spindle elongation but not chromosome segregation in C. elegans
Source: bioRxiv. 2025 Oct 14:2025.10.13.681585. Preprint. [Version 1] doi: 10.1101/2025.10.13.681585 (PMC12632992; doi:10.1101/2025.10.13.681585)

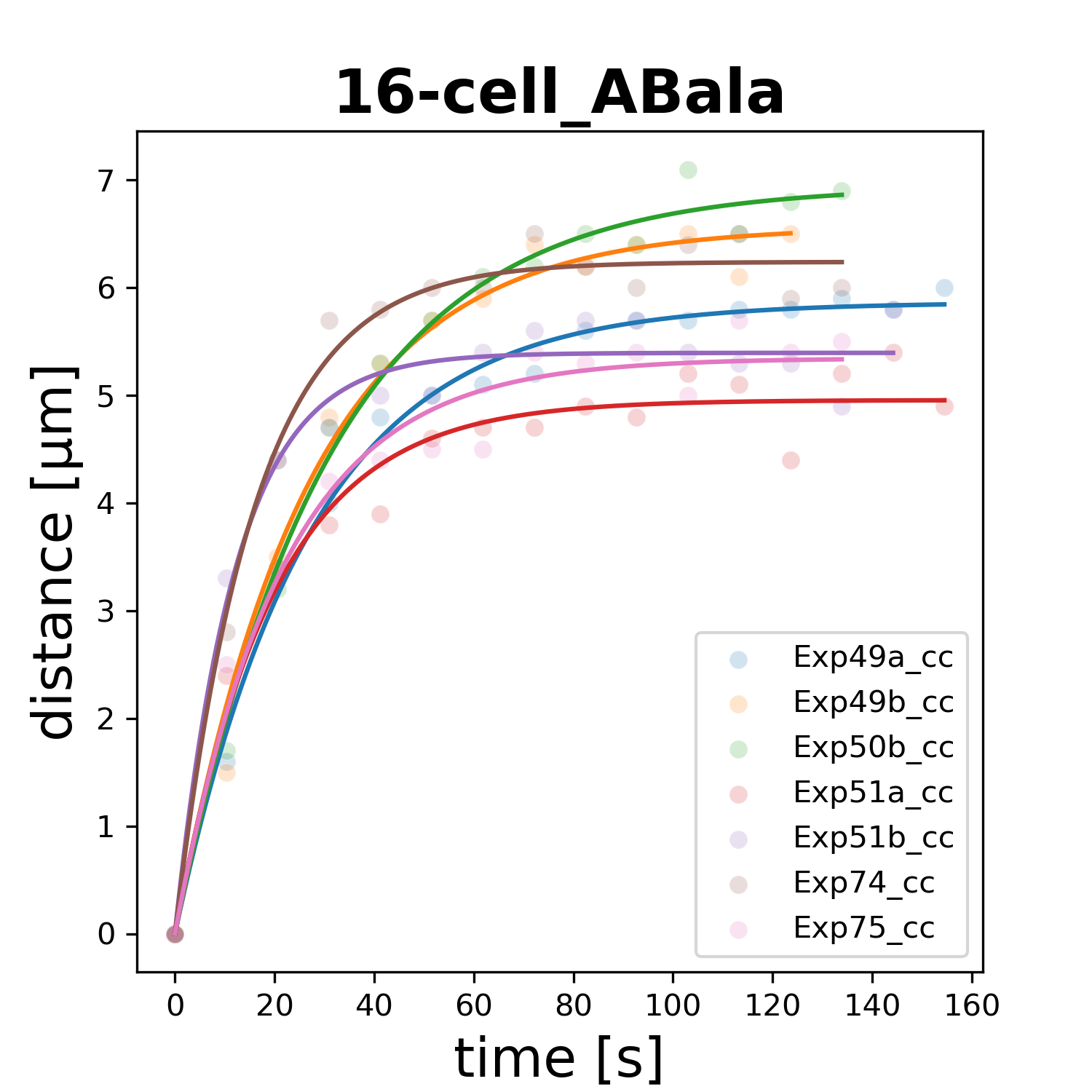

Supplement: Supplement 2 [file media-2.zip › Supplementary Material/ani2(RNAi)_chromosome_to_chromosome_distance/16-cell_ABala.png]

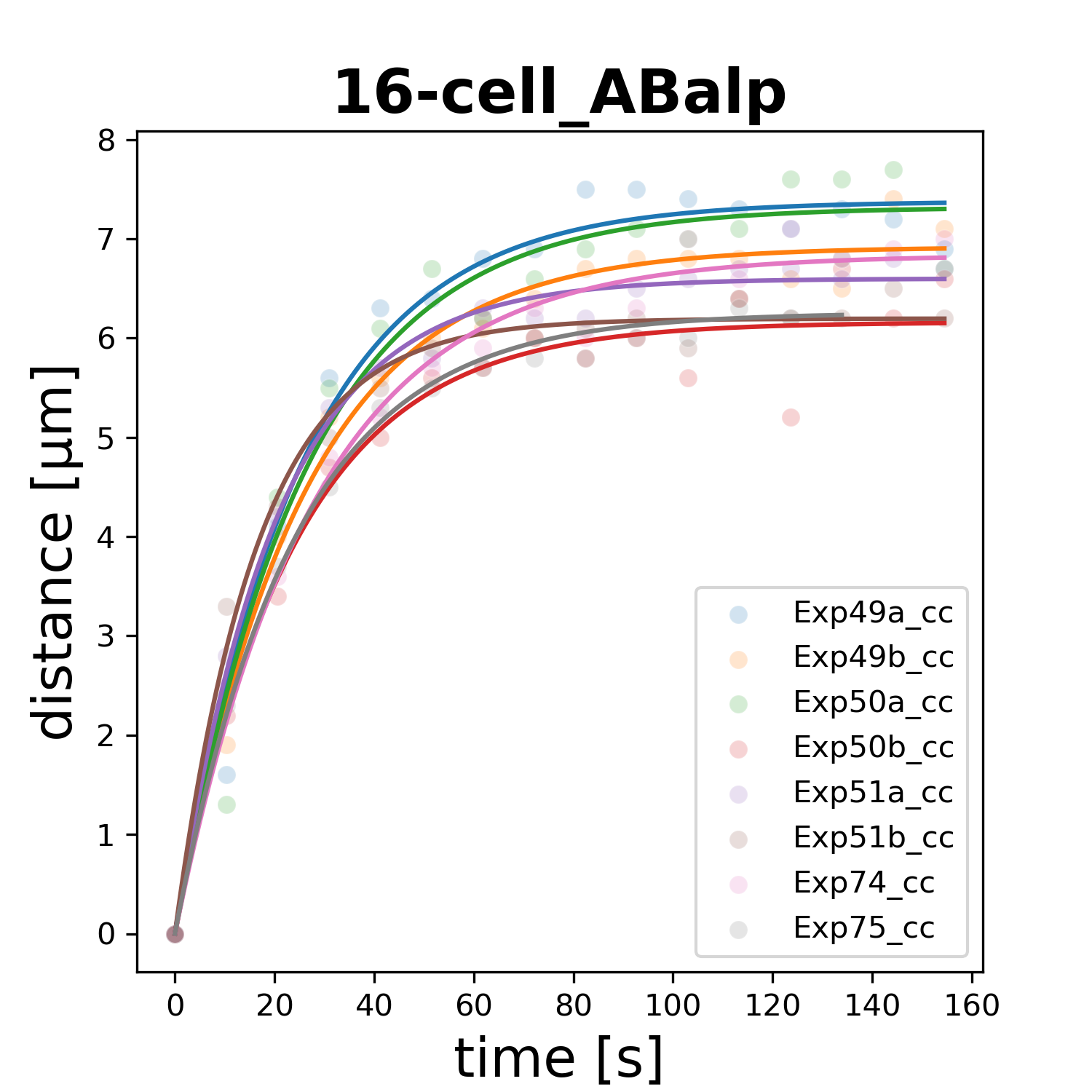

Supplement: Supplement 2 [file media-2.zip › Supplementary Material/ani2(RNAi)_chromosome_to_chromosome_distance/16-cell_ABalp.png]

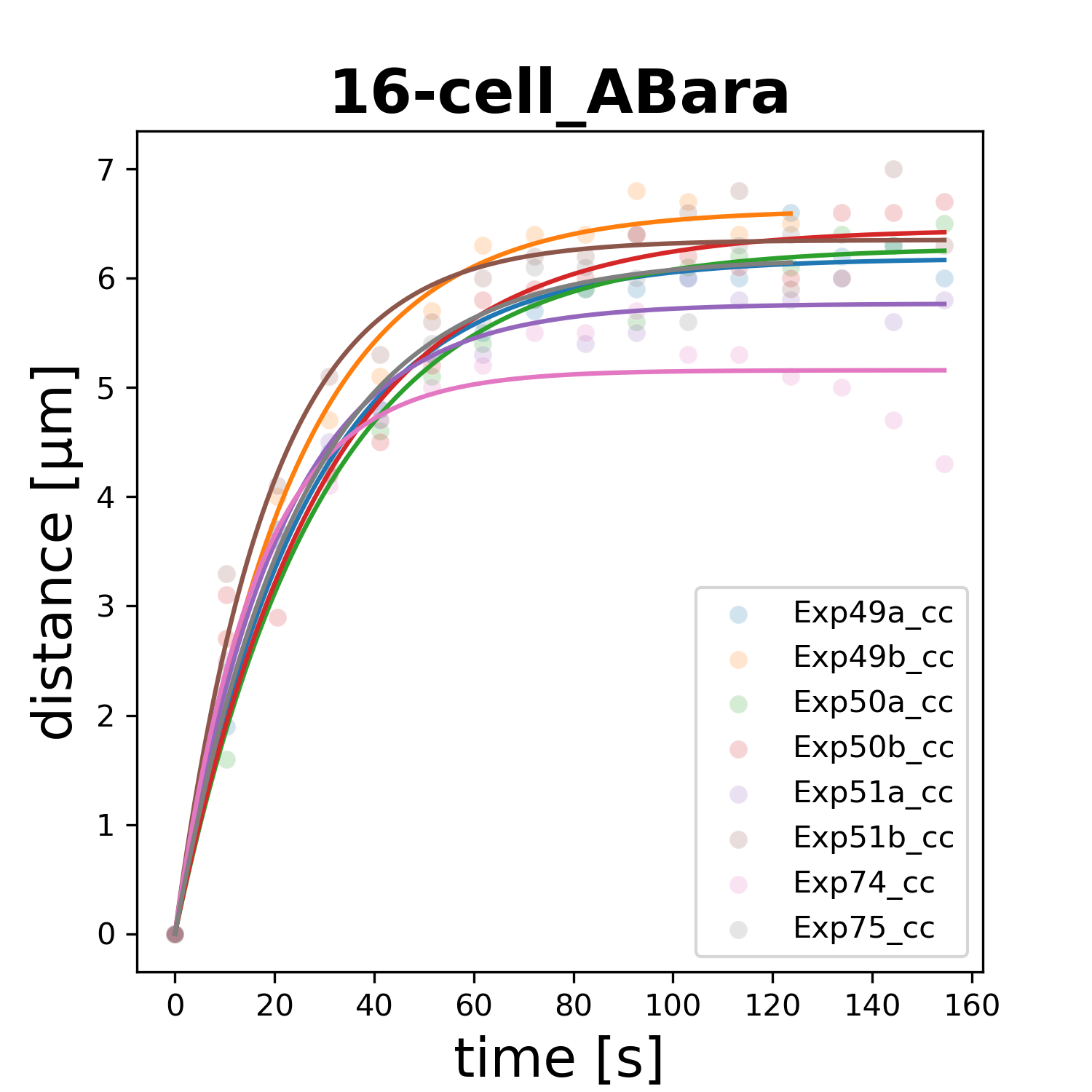

Supplement: Supplement 2 [file media-2.zip › Supplementary Material/ani2(RNAi)_chromosome_to_chromosome_distance/16-cell_ABara.png]

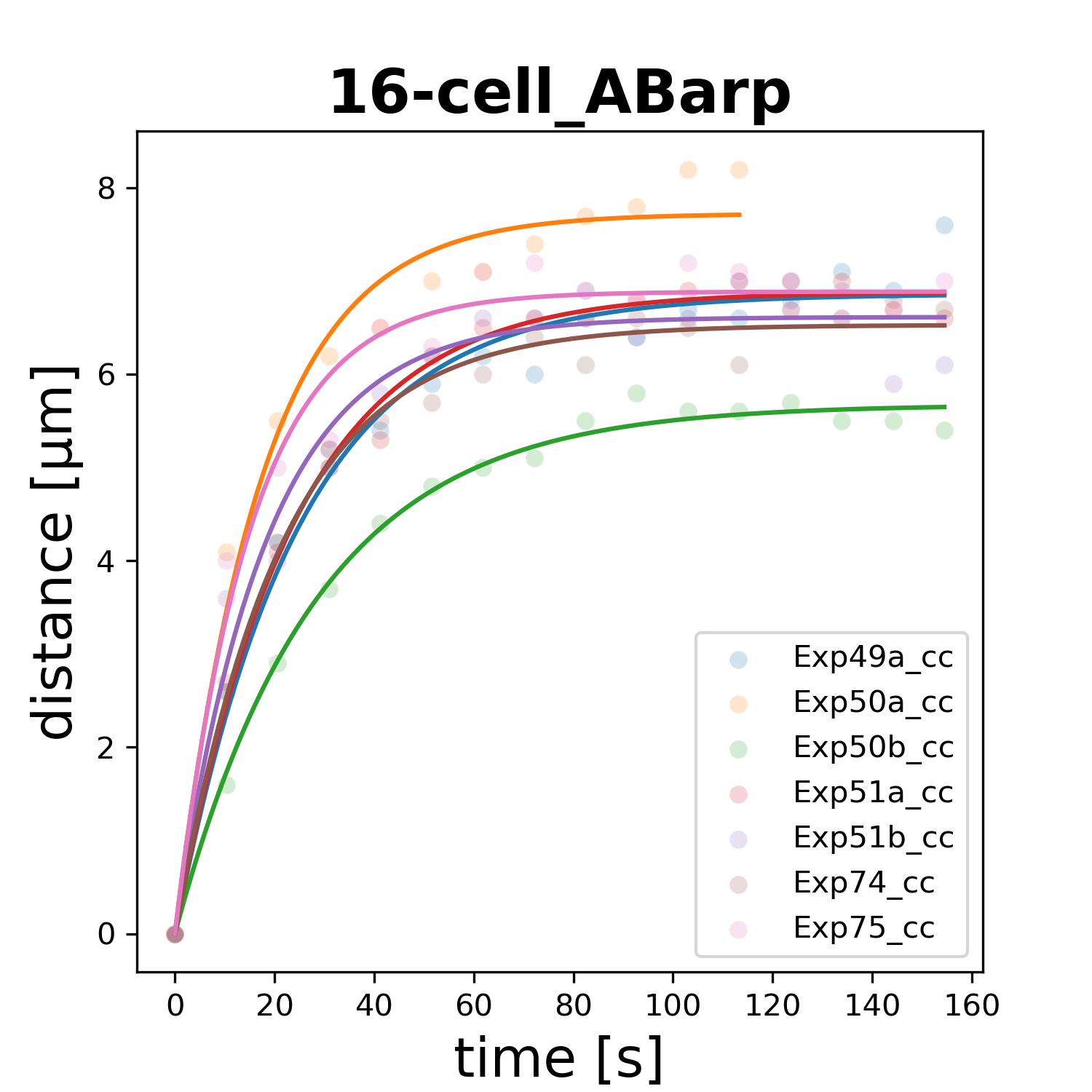

Supplement: Supplement 2 [file media-2.zip › Supplementary Material/ani2(RNAi)_chromosome_to_chromosome_distance/16-cell_ABarp.png]

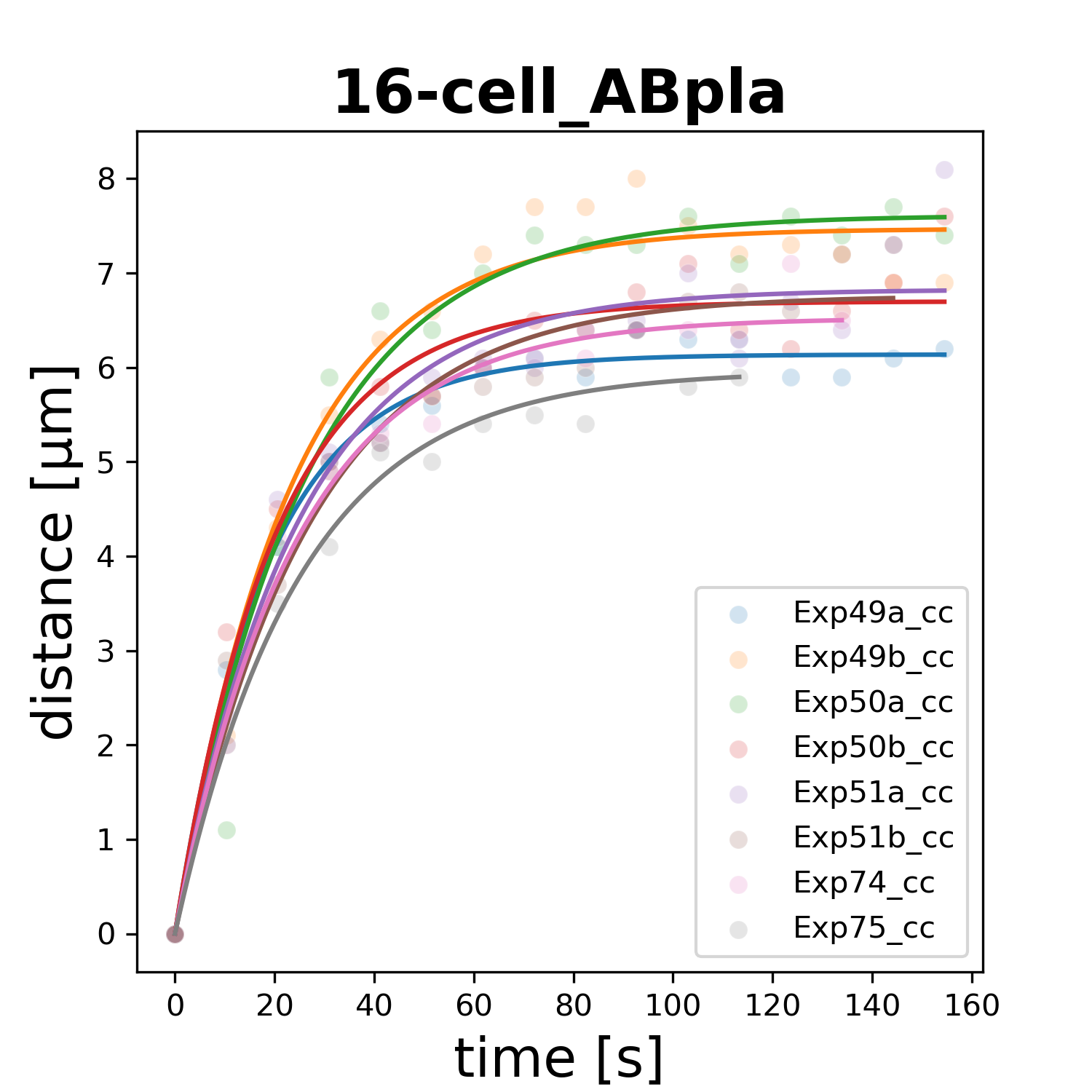

Supplement: Supplement 2 [file media-2.zip › Supplementary Material/ani2(RNAi)_chromosome_to_chromosome_distance/16-cell_ABpla.png]

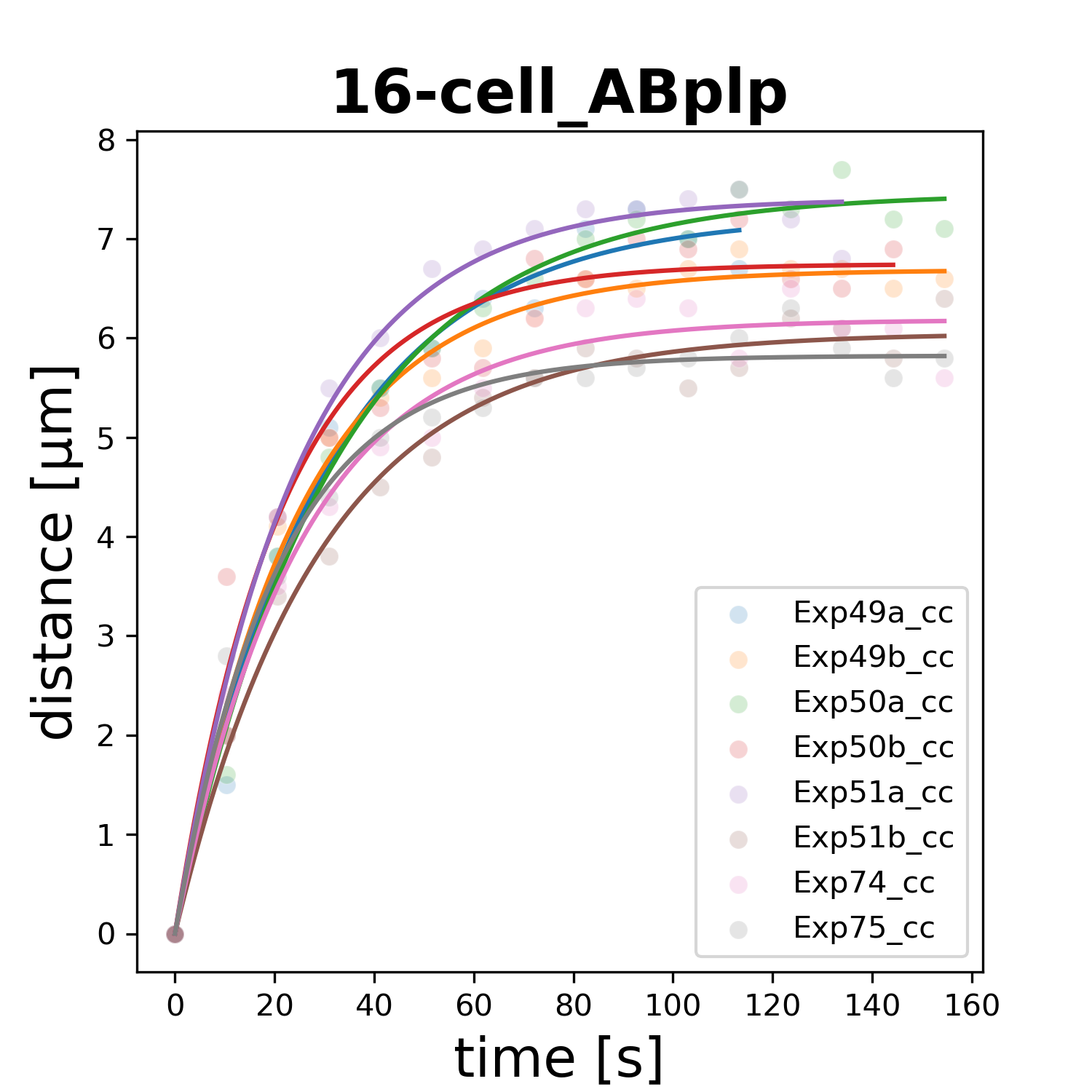

Supplement: Supplement 2 [file media-2.zip › Supplementary Material/ani2(RNAi)_chromosome_to_chromosome_distance/16-cell_ABplp.png]

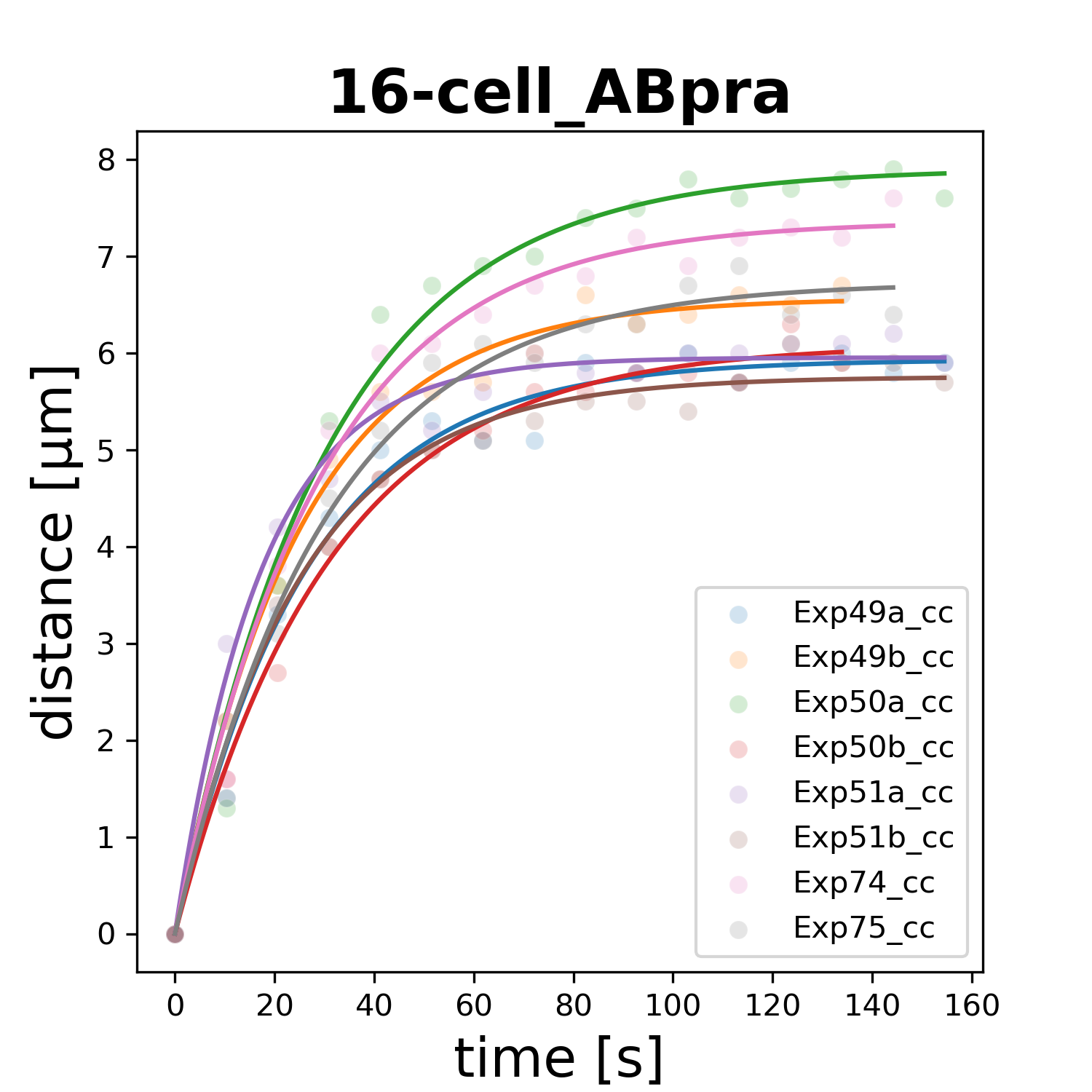

Supplement: Supplement 2 [file media-2.zip › Supplementary Material/ani2(RNAi)_chromosome_to_chromosome_distance/16-cell_ABpra.png]

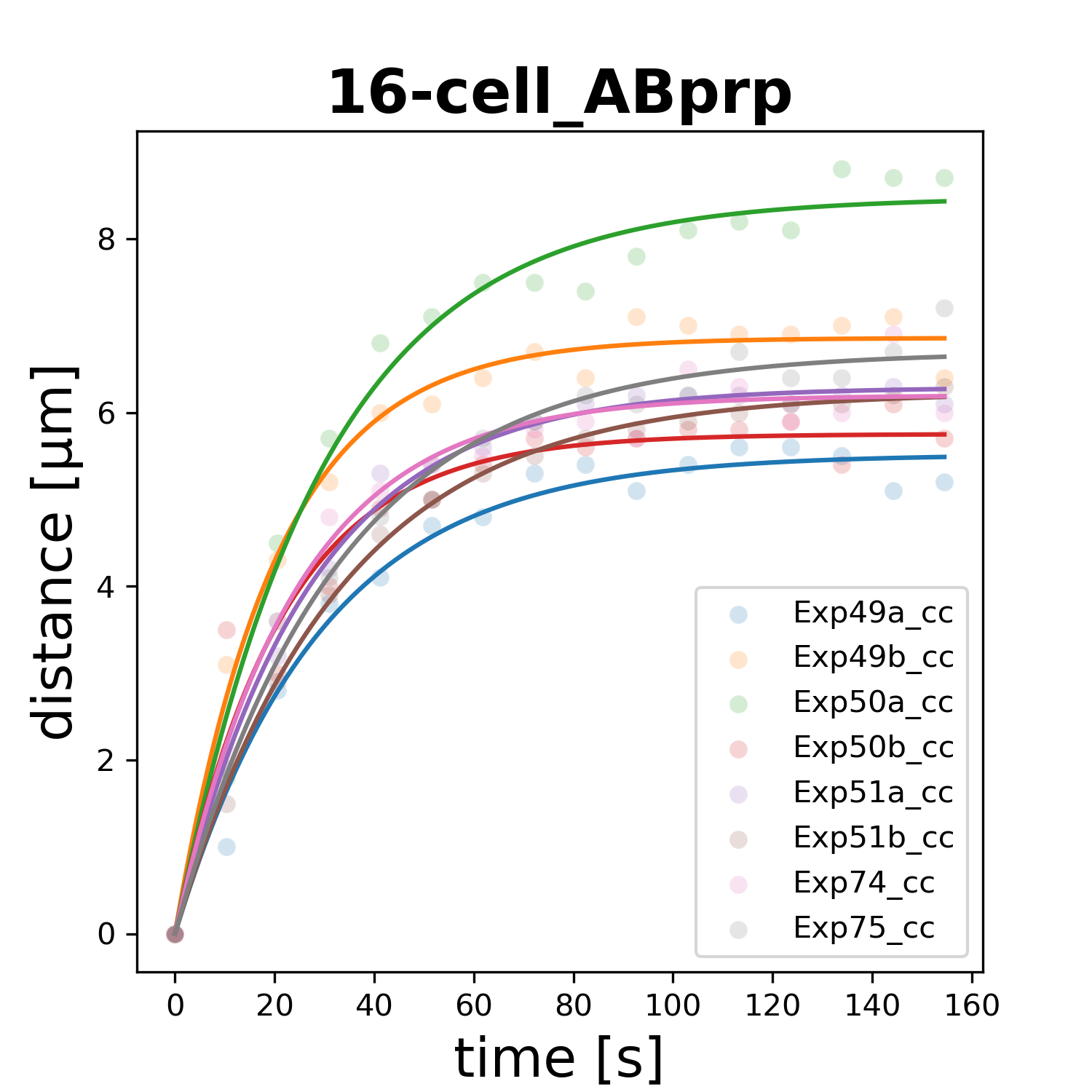

Supplement: Supplement 2 [file media-2.zip › Supplementary Material/ani2(RNAi)_chromosome_to_chromosome_distance/16-cell_ABprp.png]

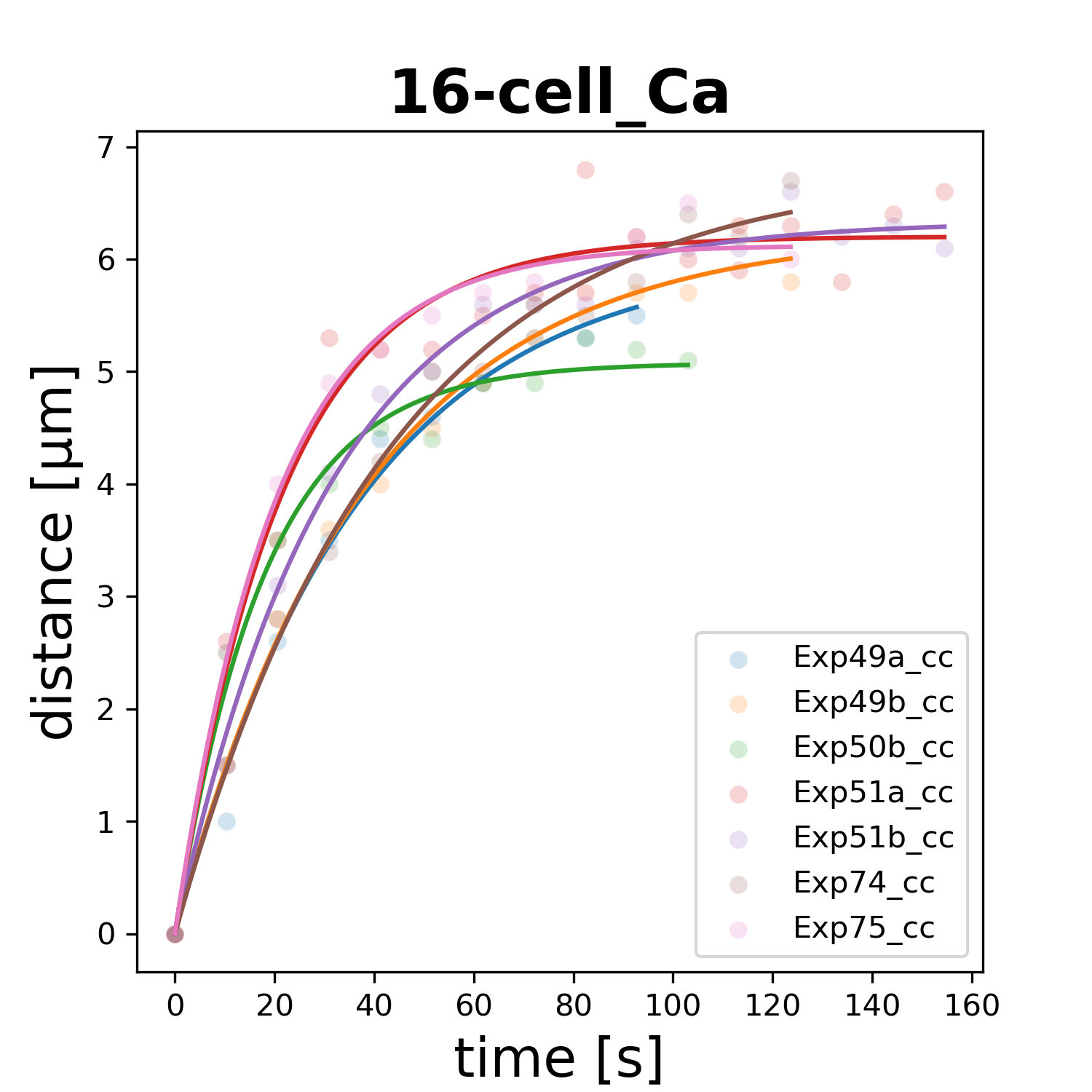

Supplement: Supplement 2 [file media-2.zip › Supplementary Material/ani2(RNAi)_chromosome_to_chromosome_distance/16-cell_Ca.png]

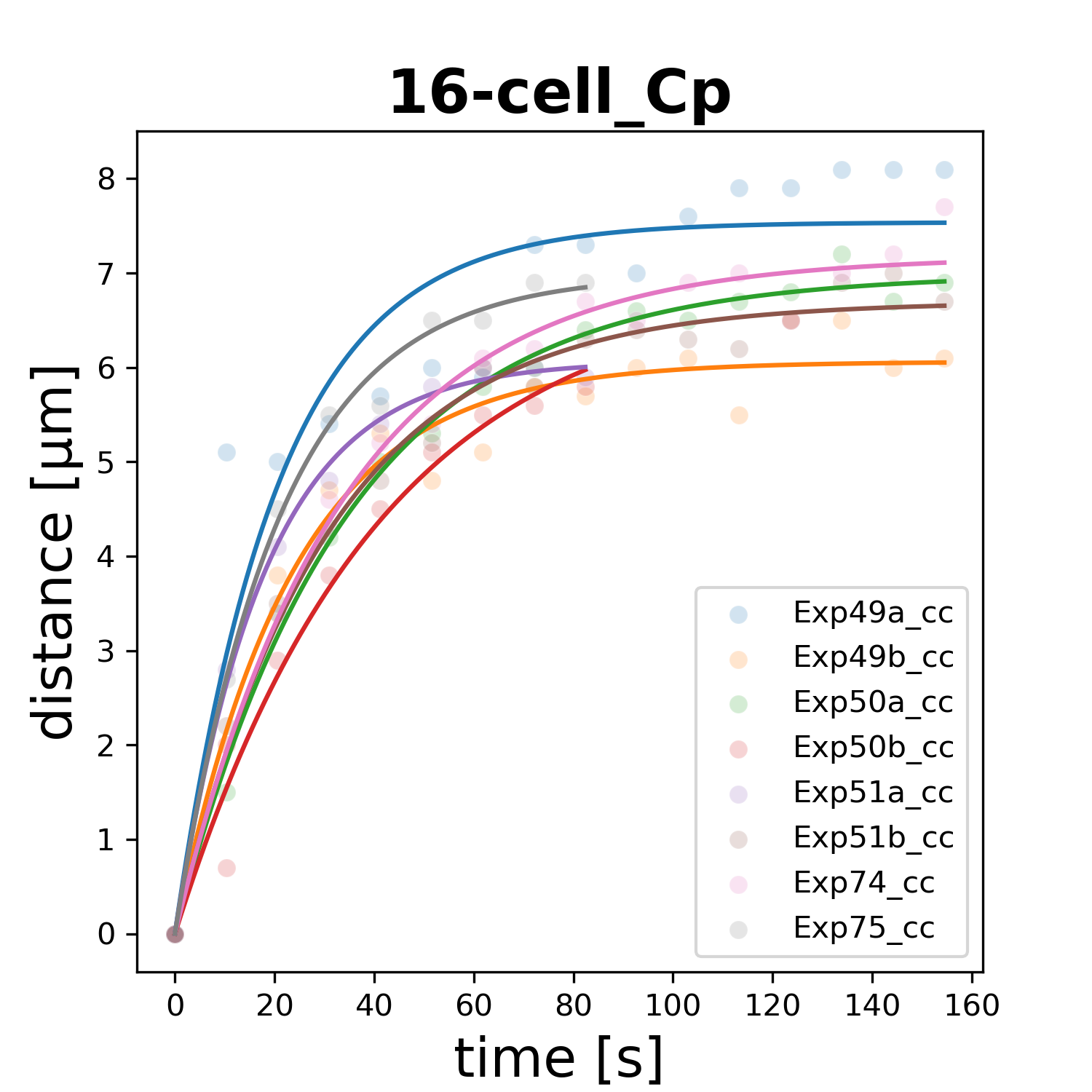

Supplement: Supplement 2 [file media-2.zip › Supplementary Material/ani2(RNAi)_chromosome_to_chromosome_distance/16-cell_Cp.png]

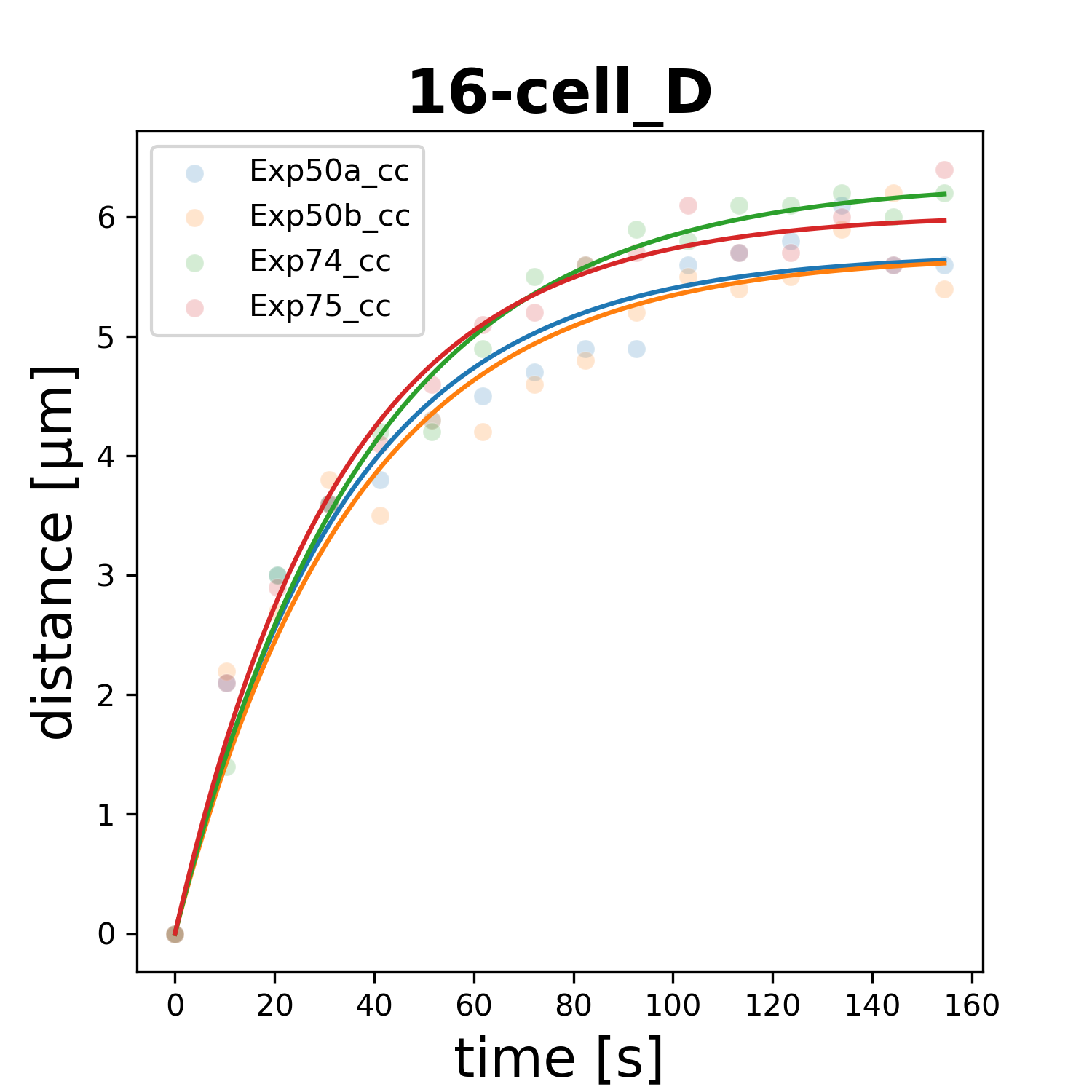

Supplement: Supplement 2 [file media-2.zip › Supplementary Material/ani2(RNAi)_chromosome_to_chromosome_distance/16-cell_D.png]

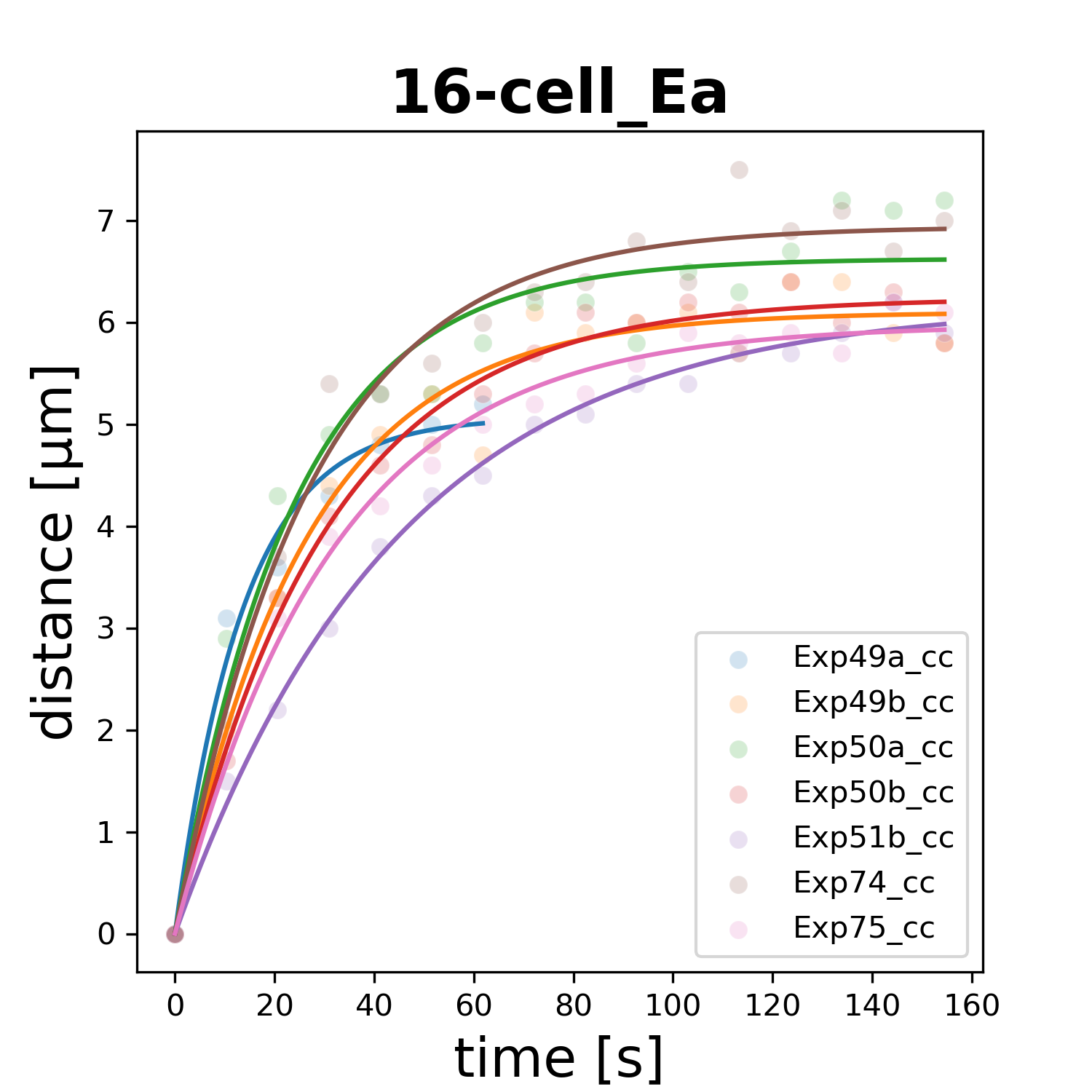

Supplement: Supplement 2 [file media-2.zip › Supplementary Material/ani2(RNAi)_chromosome_to_chromosome_distance/16-cell_Ea.png]

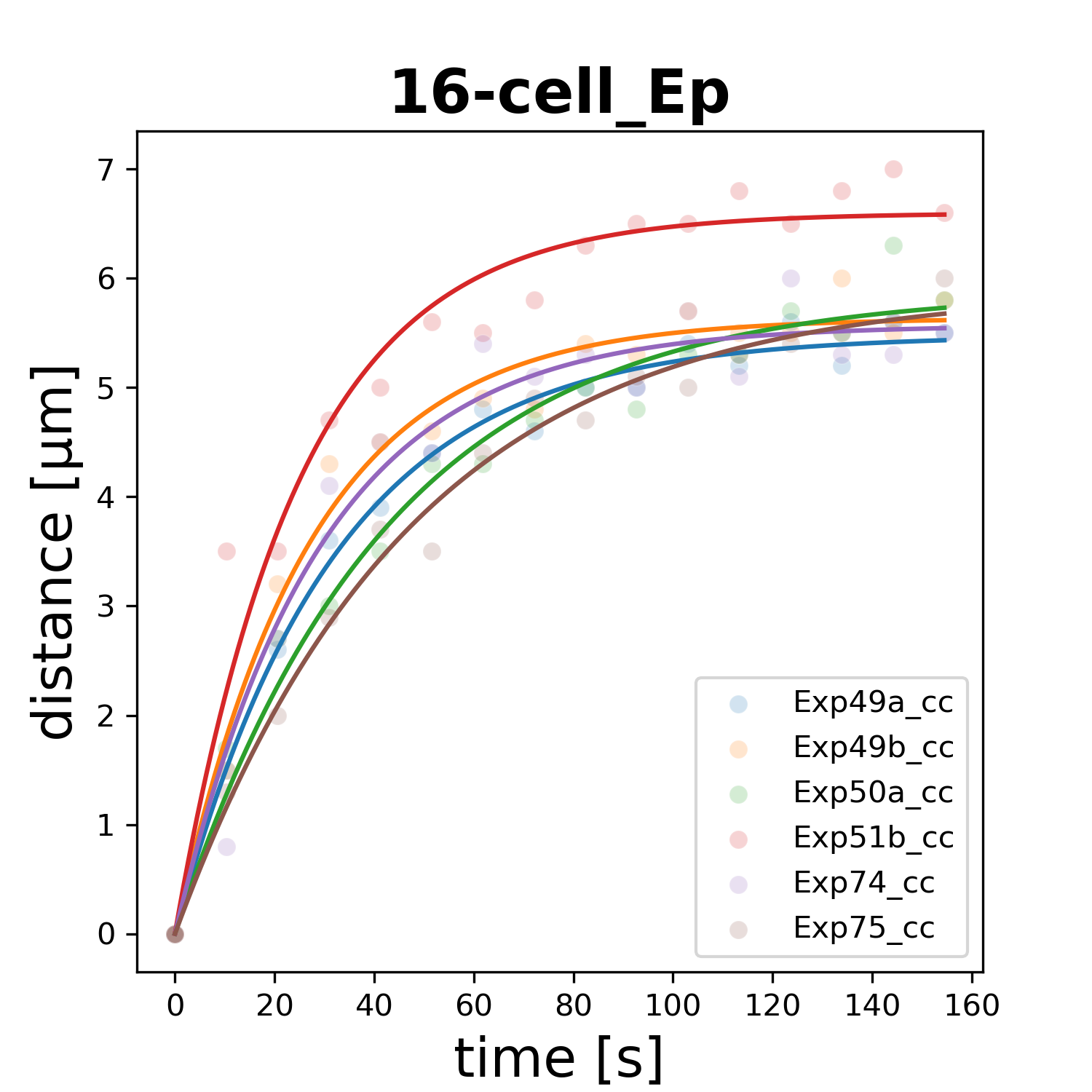

Supplement: Supplement 2 [file media-2.zip › Supplementary Material/ani2(RNAi)_chromosome_to_chromosome_distance/16-cell_Ep.png]

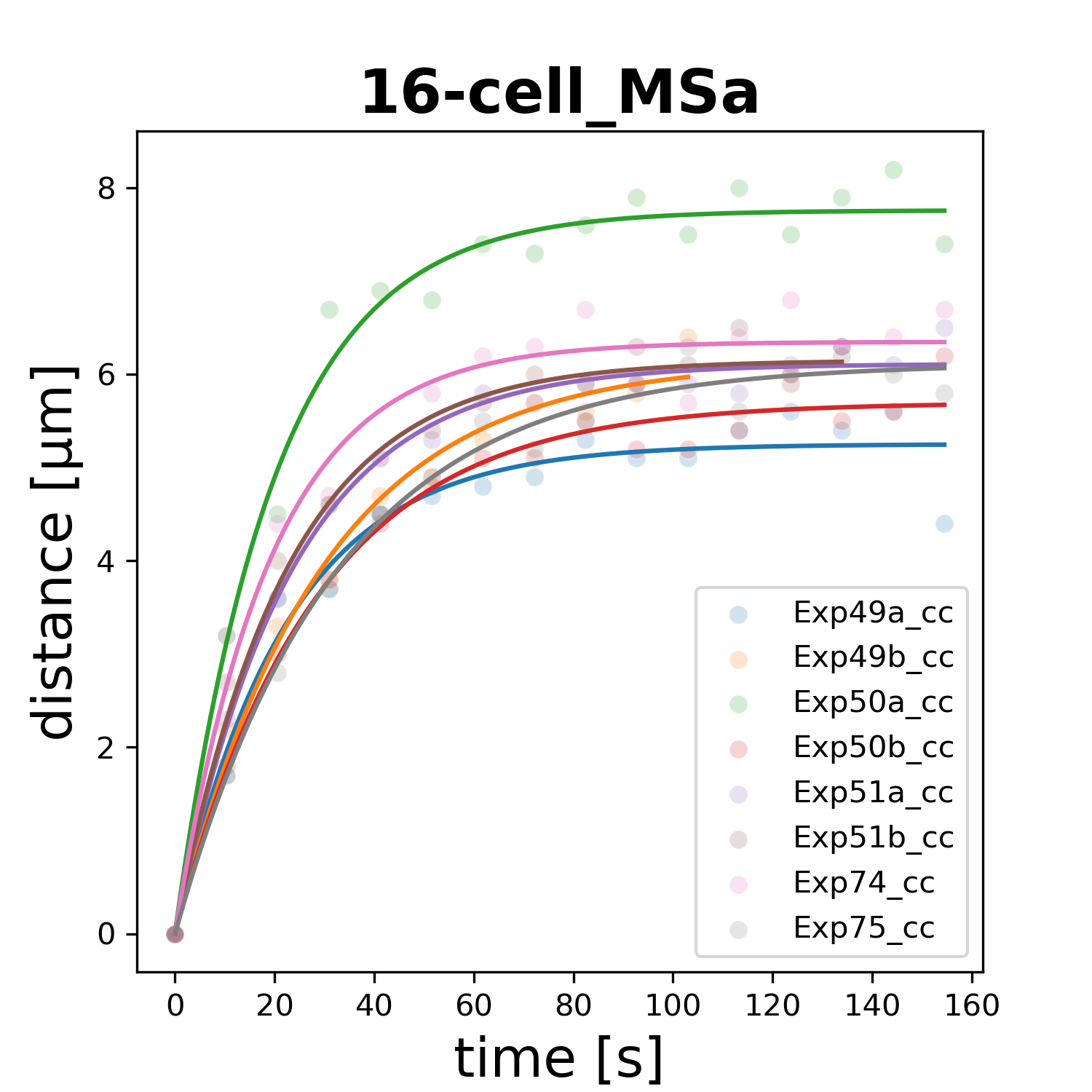

Supplement: Supplement 2 [file media-2.zip › Supplementary Material/ani2(RNAi)_chromosome_to_chromosome_distance/16-cell_MSa.png]

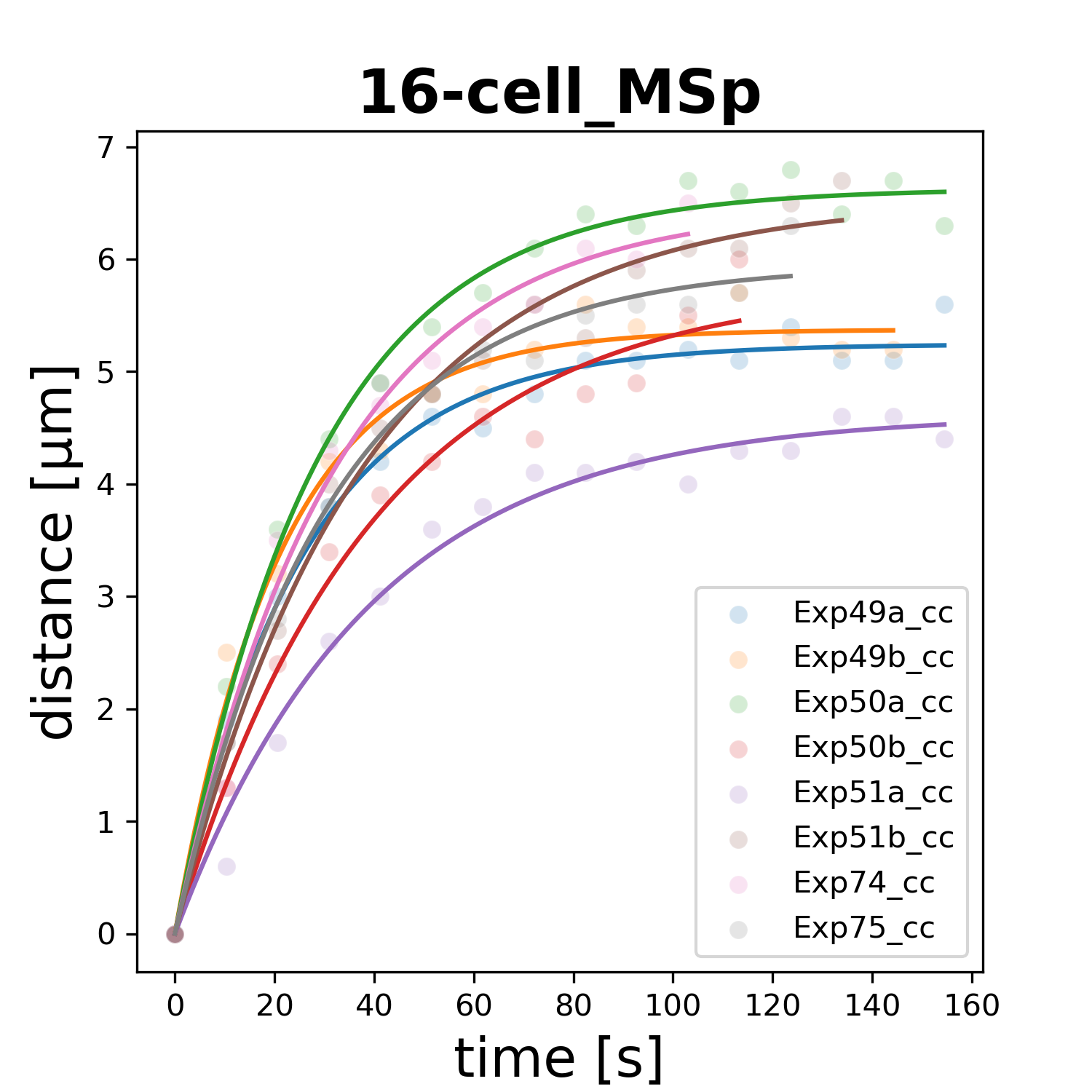

Supplement: Supplement 2 [file media-2.zip › Supplementary Material/ani2(RNAi)_chromosome_to_chromosome_distance/16-cell_MSp.png]

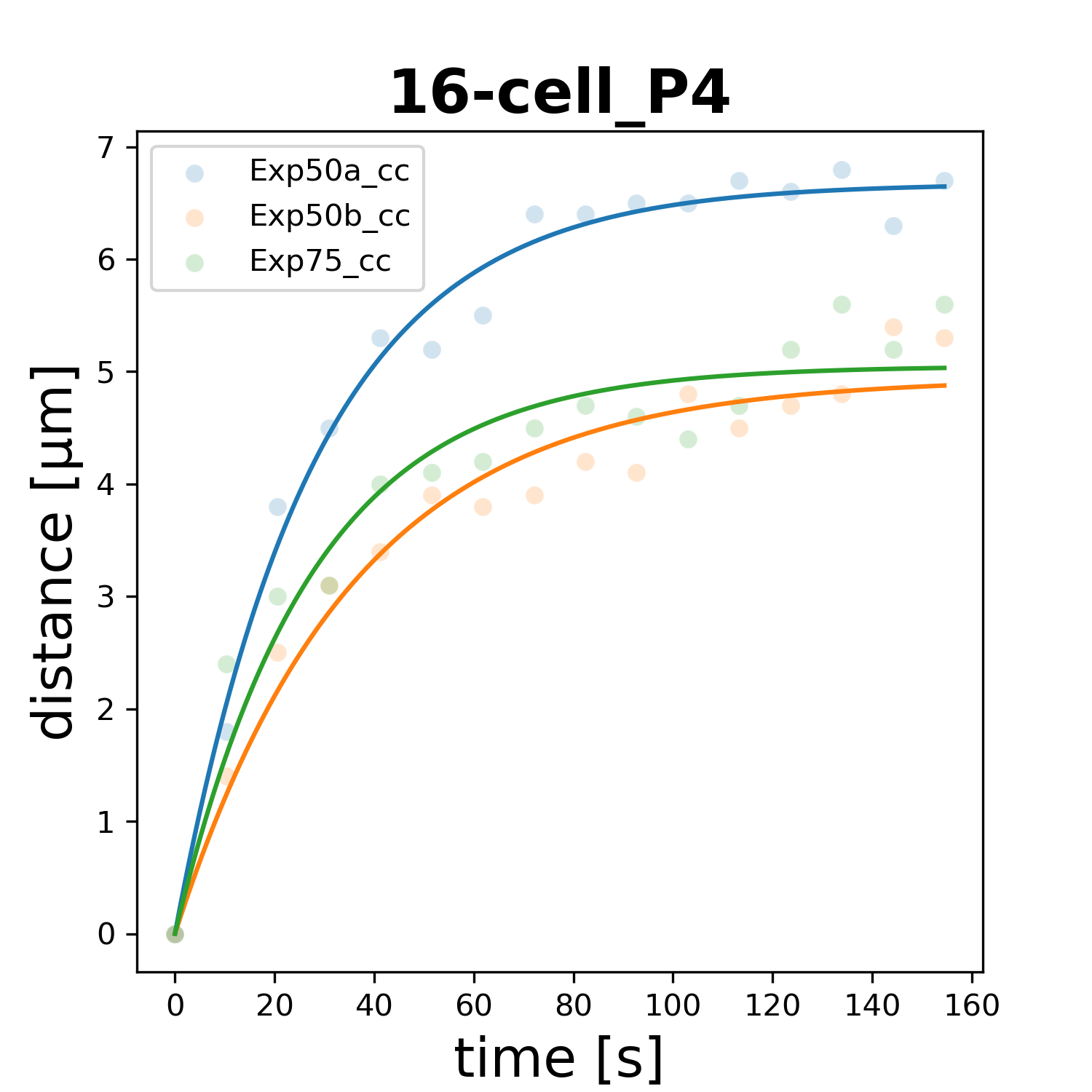

Supplement: Supplement 2 [file media-2.zip › Supplementary Material/ani2(RNAi)_chromosome_to_chromosome_distance/16-cell_P4.png]

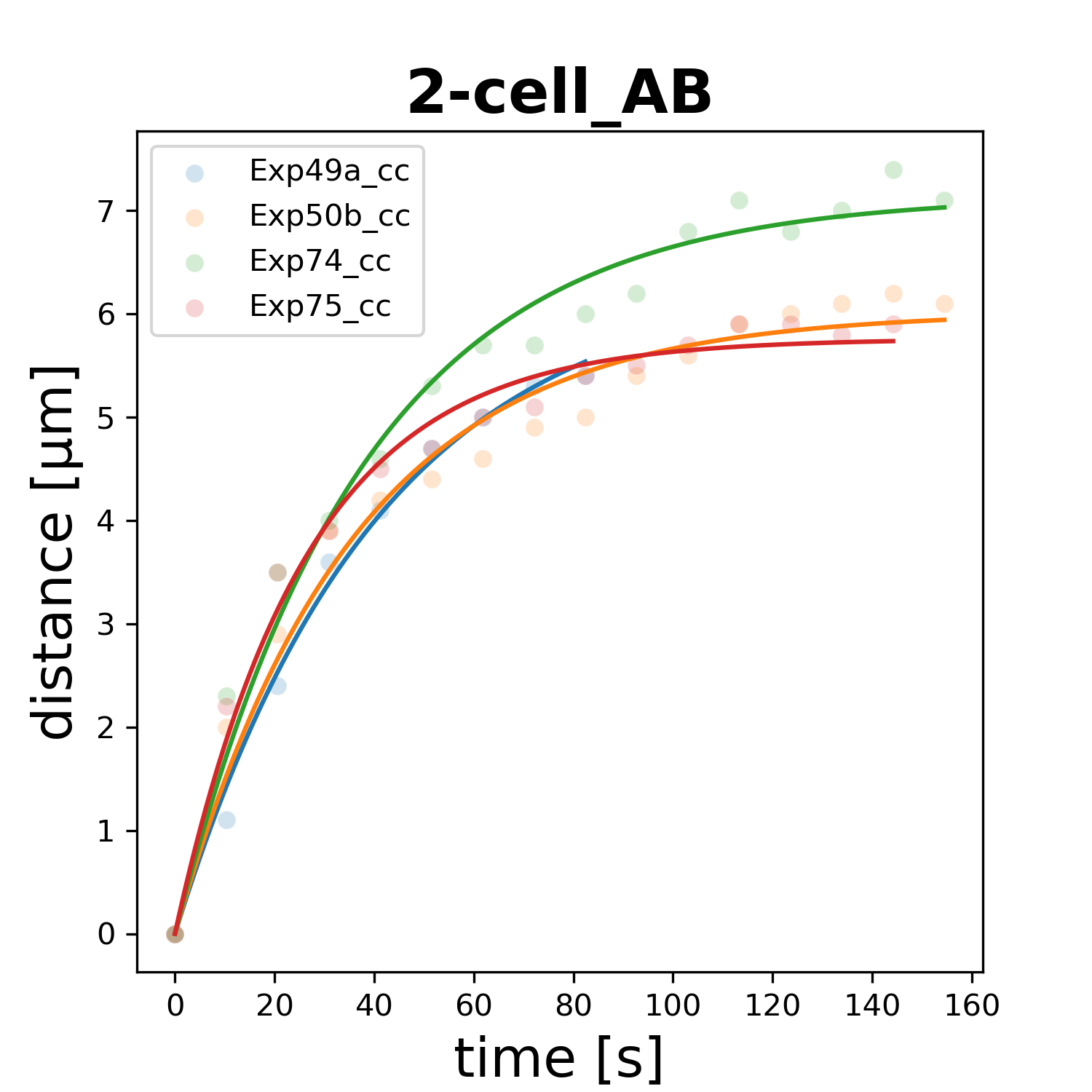

Supplement: Supplement 2 [file media-2.zip › Supplementary Material/ani2(RNAi)_chromosome_to_chromosome_distance/2-cell_AB.png]

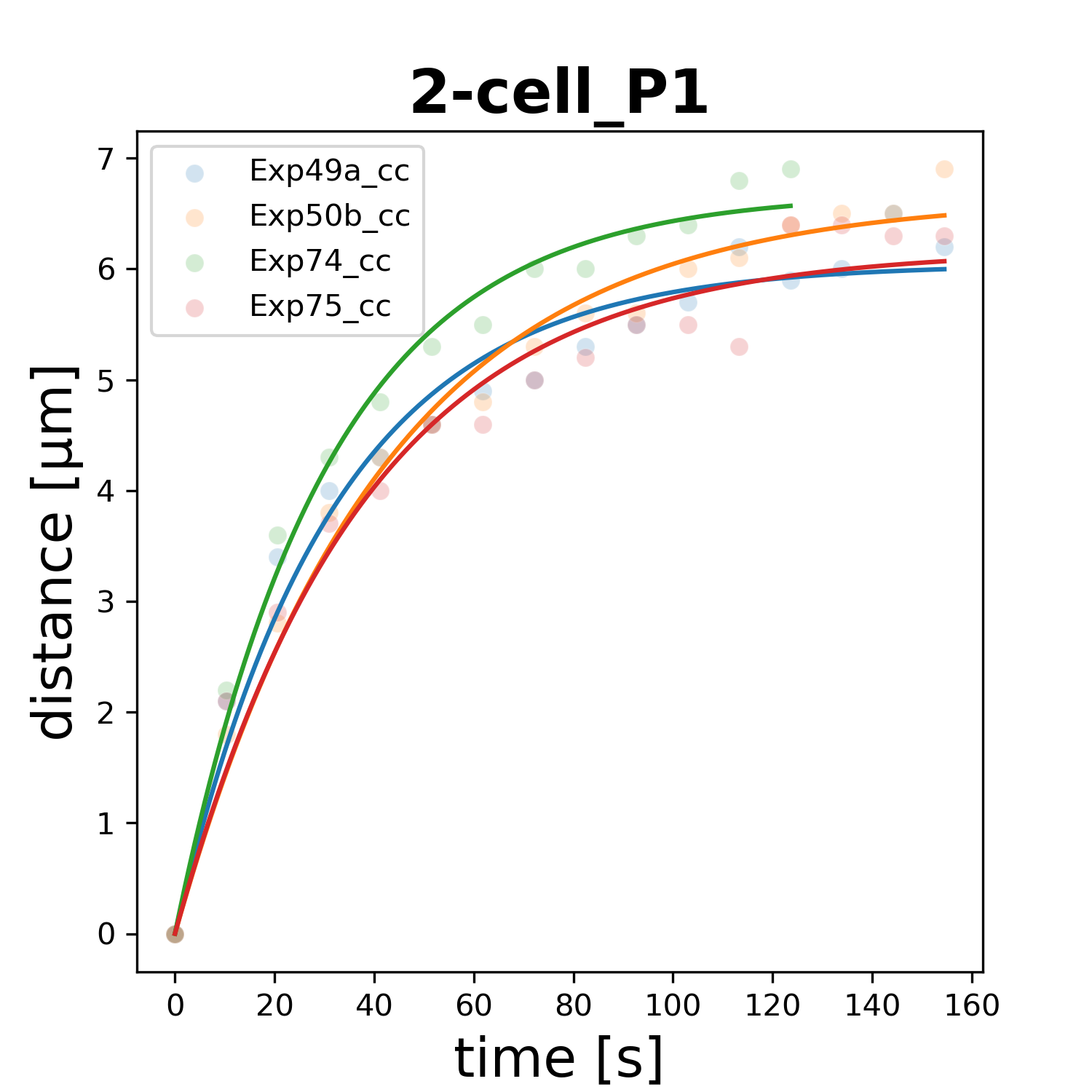

Supplement: Supplement 2 [file media-2.zip › Supplementary Material/ani2(RNAi)_chromosome_to_chromosome_distance/2-cell_P1.png]

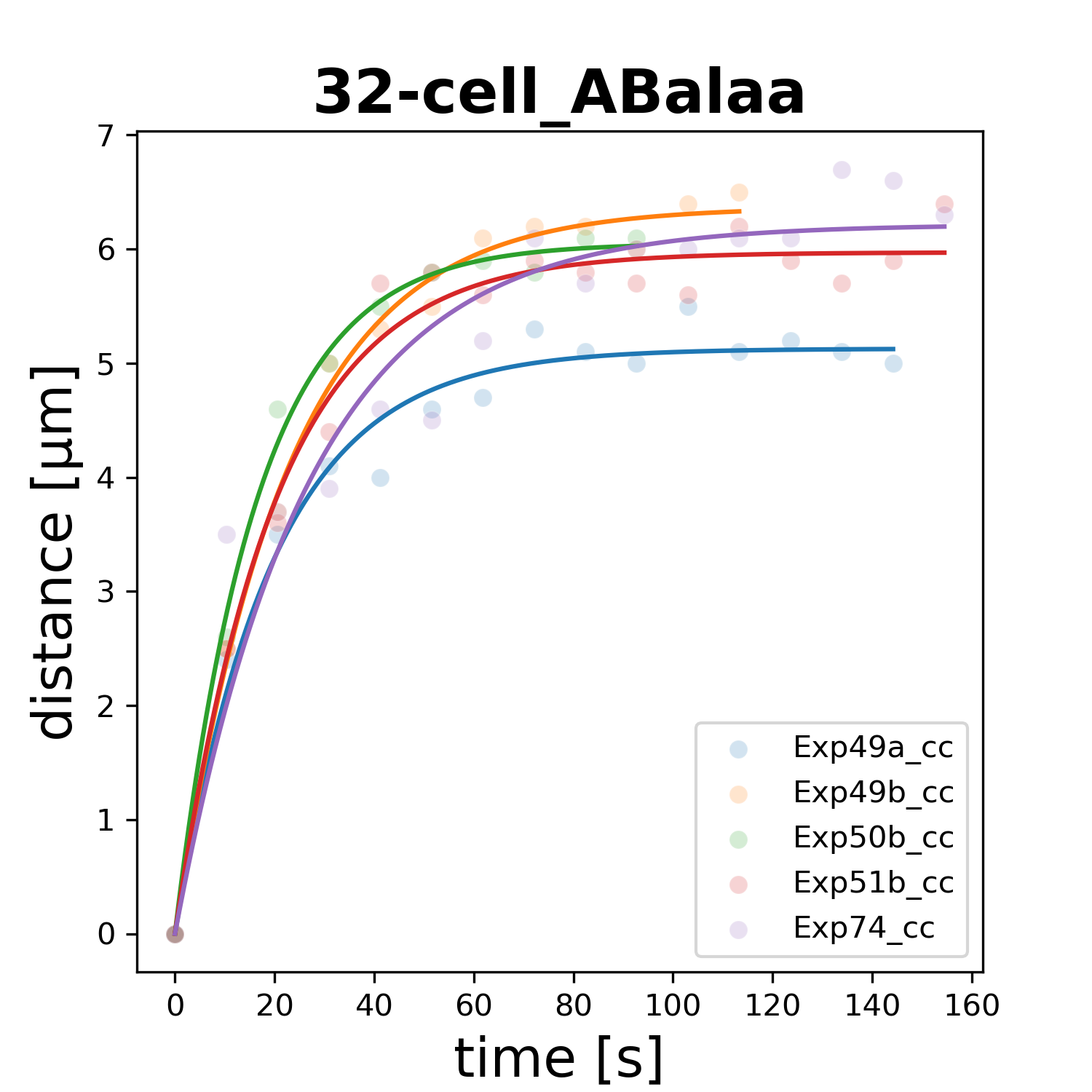

Supplement: Supplement 2 [file media-2.zip › Supplementary Material/ani2(RNAi)_chromosome_to_chromosome_distance/32-cell_ABalaa.png]

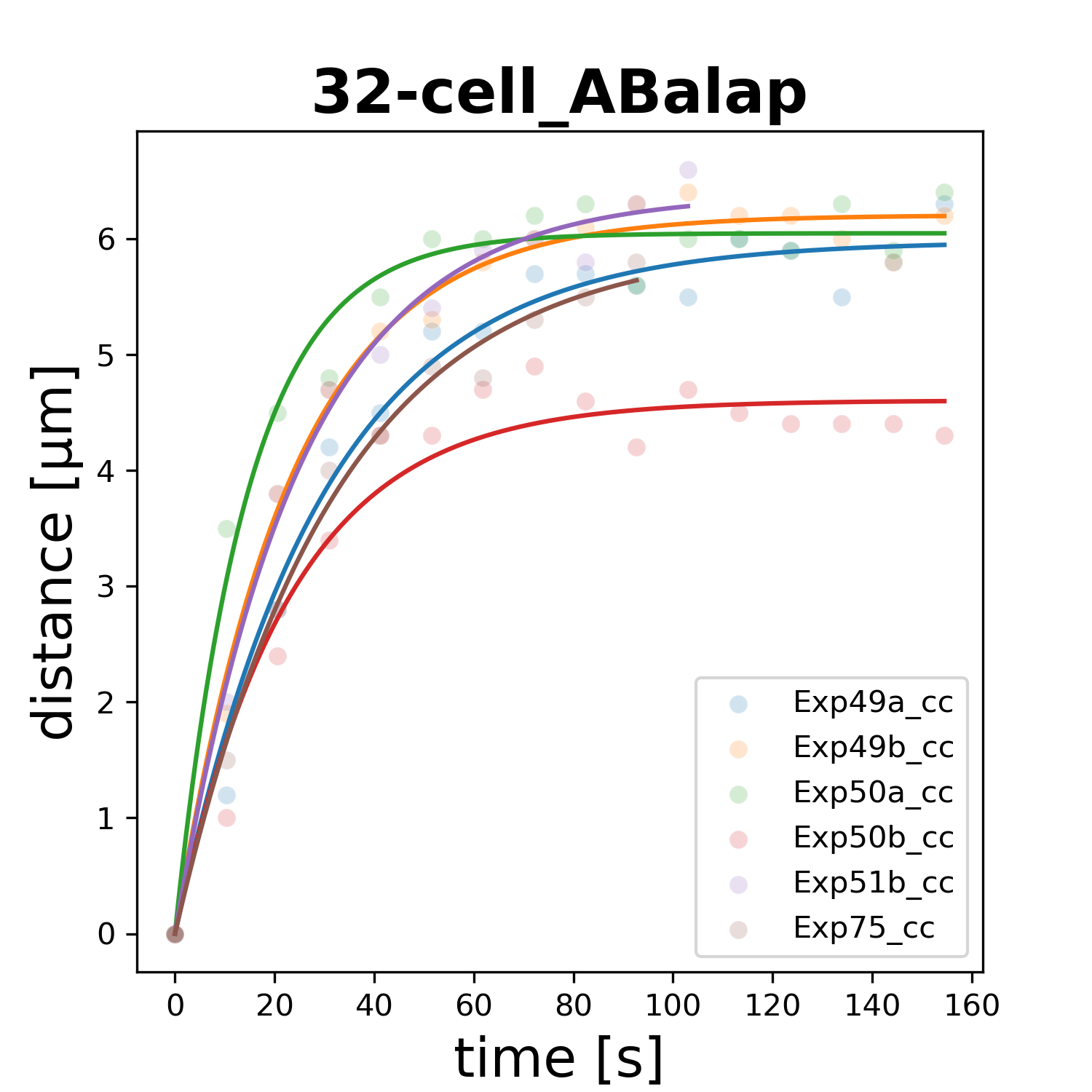

Supplement: Supplement 2 [file media-2.zip › Supplementary Material/ani2(RNAi)_chromosome_to_chromosome_distance/32-cell_ABalap.png]

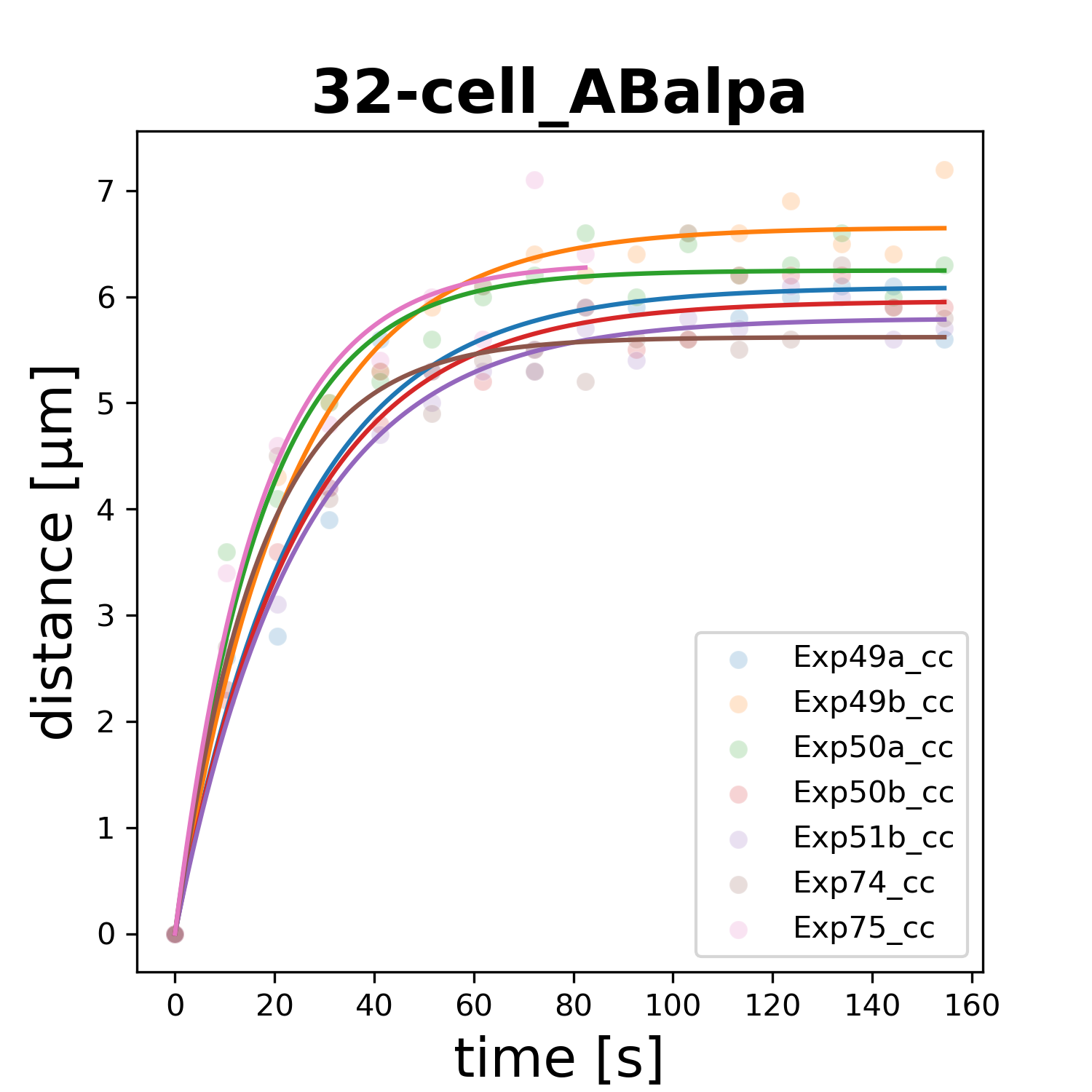

Supplement: Supplement 2 [file media-2.zip › Supplementary Material/ani2(RNAi)_chromosome_to_chromosome_distance/32-cell_ABalpa.png]

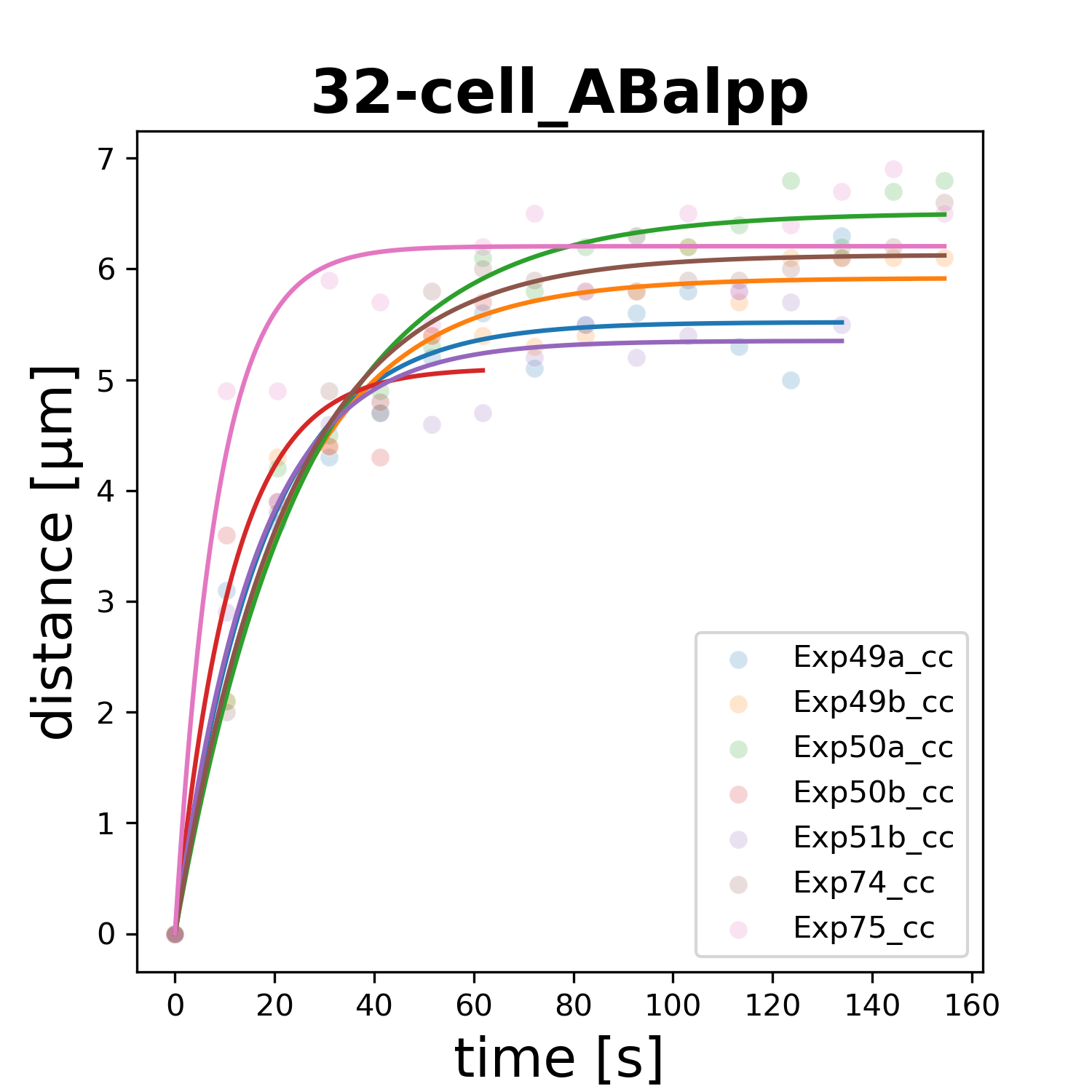

Supplement: Supplement 2 [file media-2.zip › Supplementary Material/ani2(RNAi)_chromosome_to_chromosome_distance/32-cell_ABalpp.png]

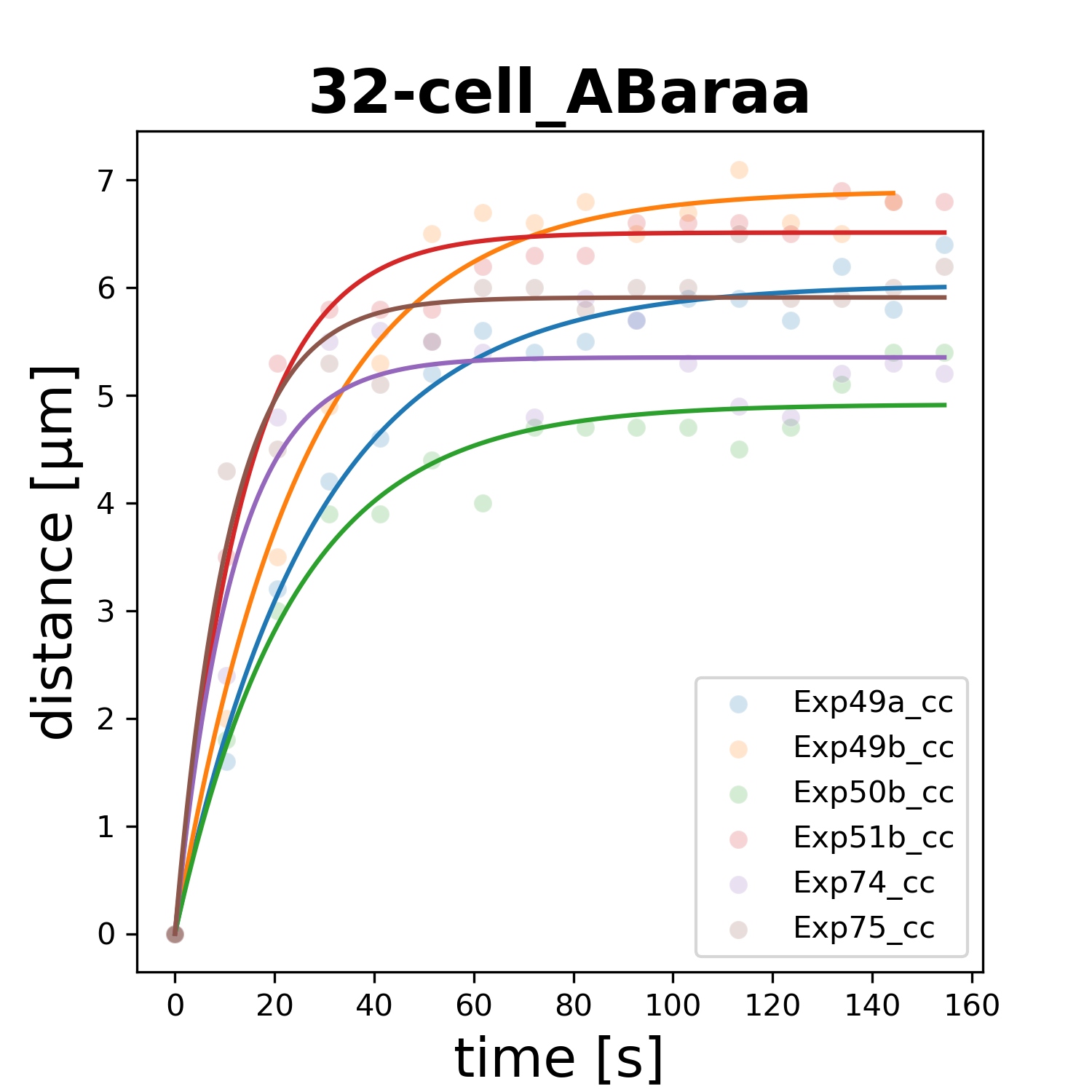

Supplement: Supplement 2 [file media-2.zip › Supplementary Material/ani2(RNAi)_chromosome_to_chromosome_distance/32-cell_ABaraa.png]

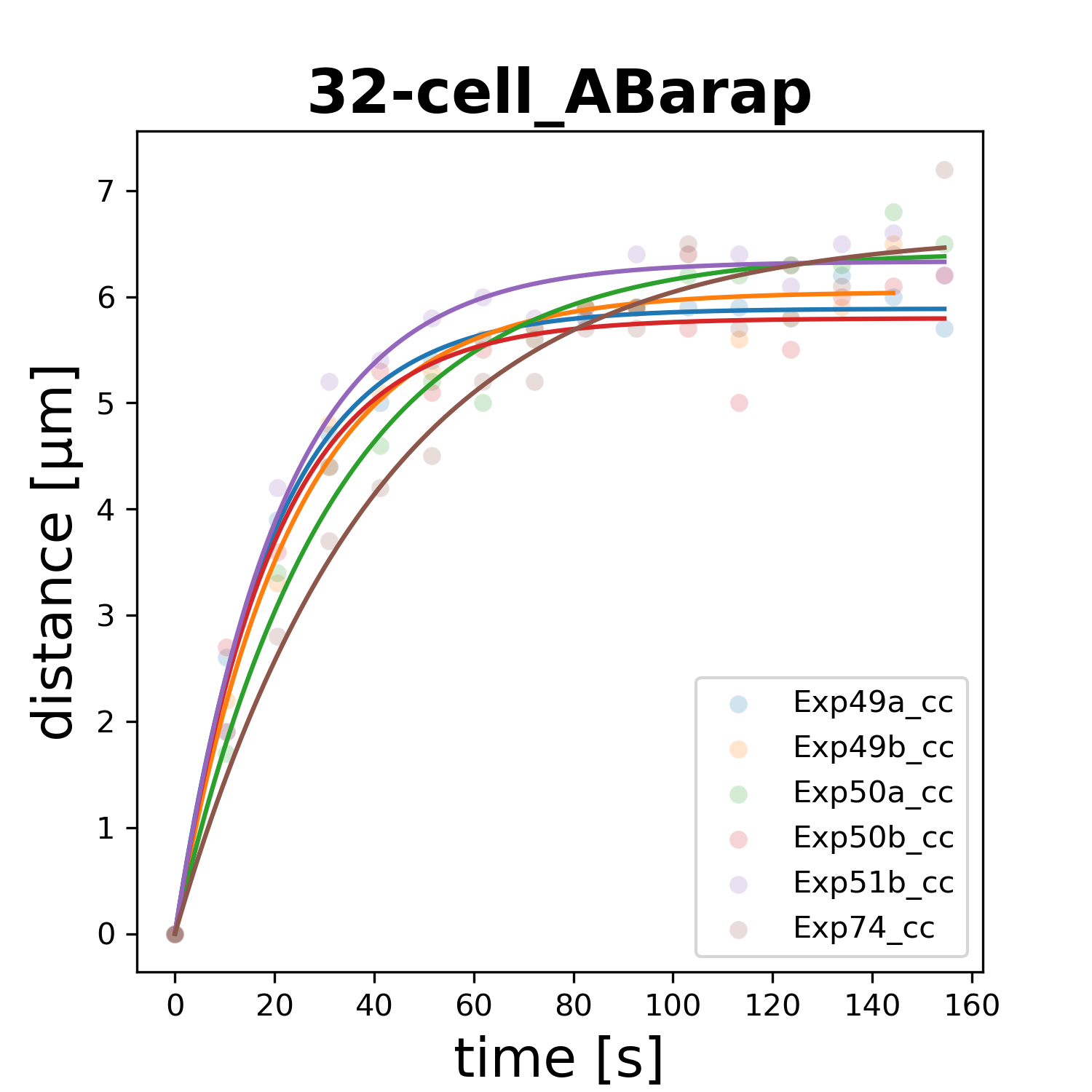

Supplement: Supplement 2 [file media-2.zip › Supplementary Material/ani2(RNAi)_chromosome_to_chromosome_distance/32-cell_ABarap.png]

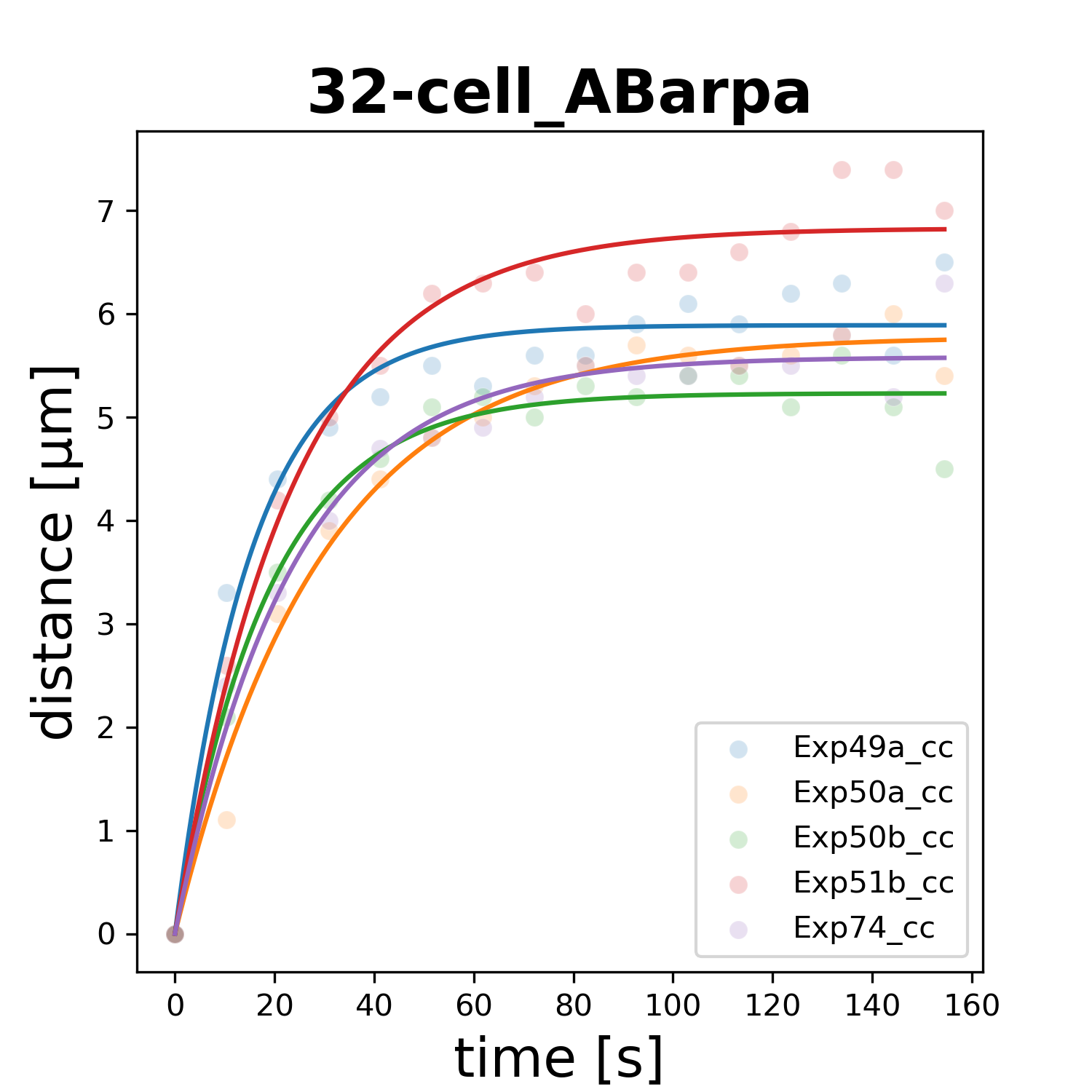

Supplement: Supplement 2 [file media-2.zip › Supplementary Material/ani2(RNAi)_chromosome_to_chromosome_distance/32-cell_ABarpa.png]

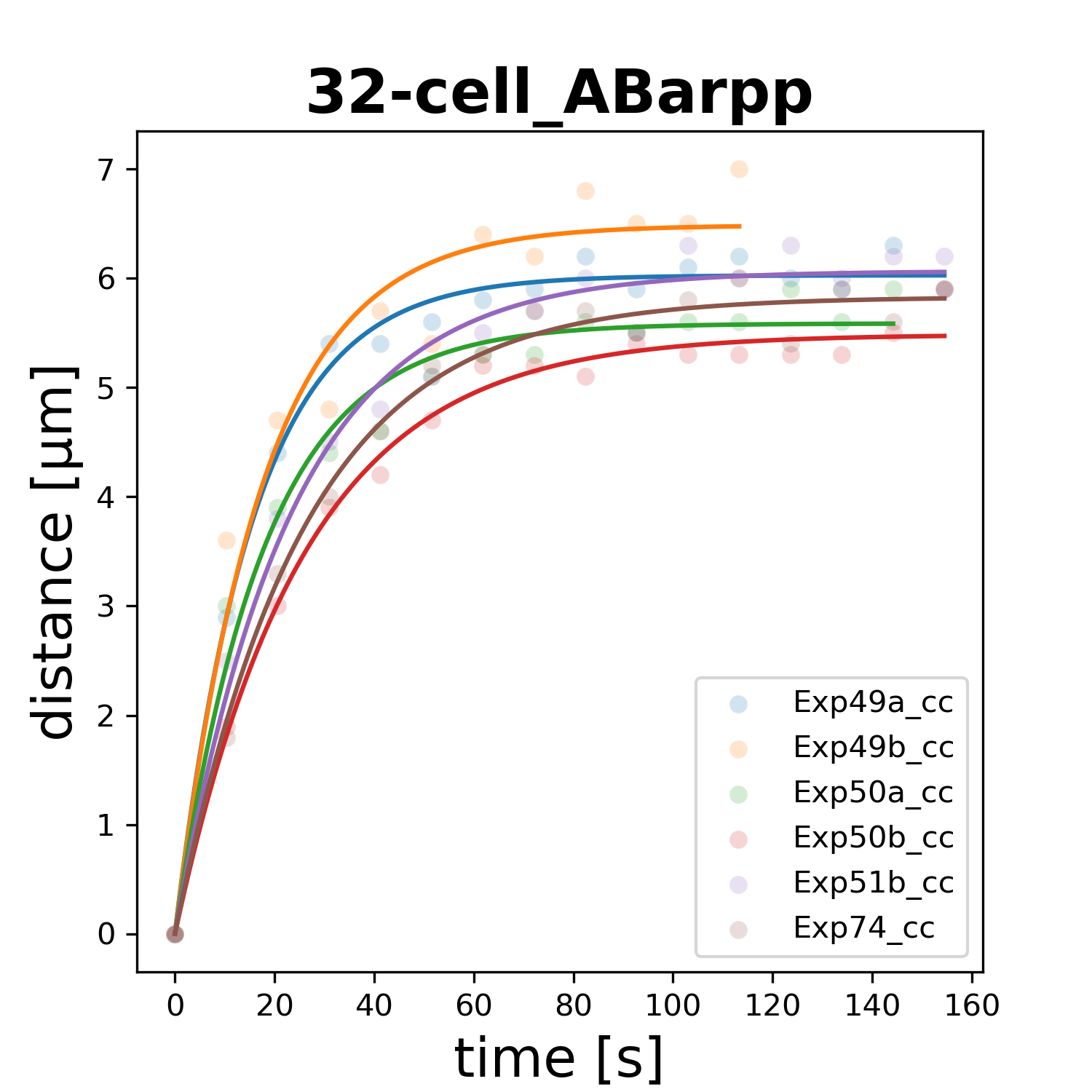

Supplement: Supplement 2 [file media-2.zip › Supplementary Material/ani2(RNAi)_chromosome_to_chromosome_distance/32-cell_ABarpp.png]

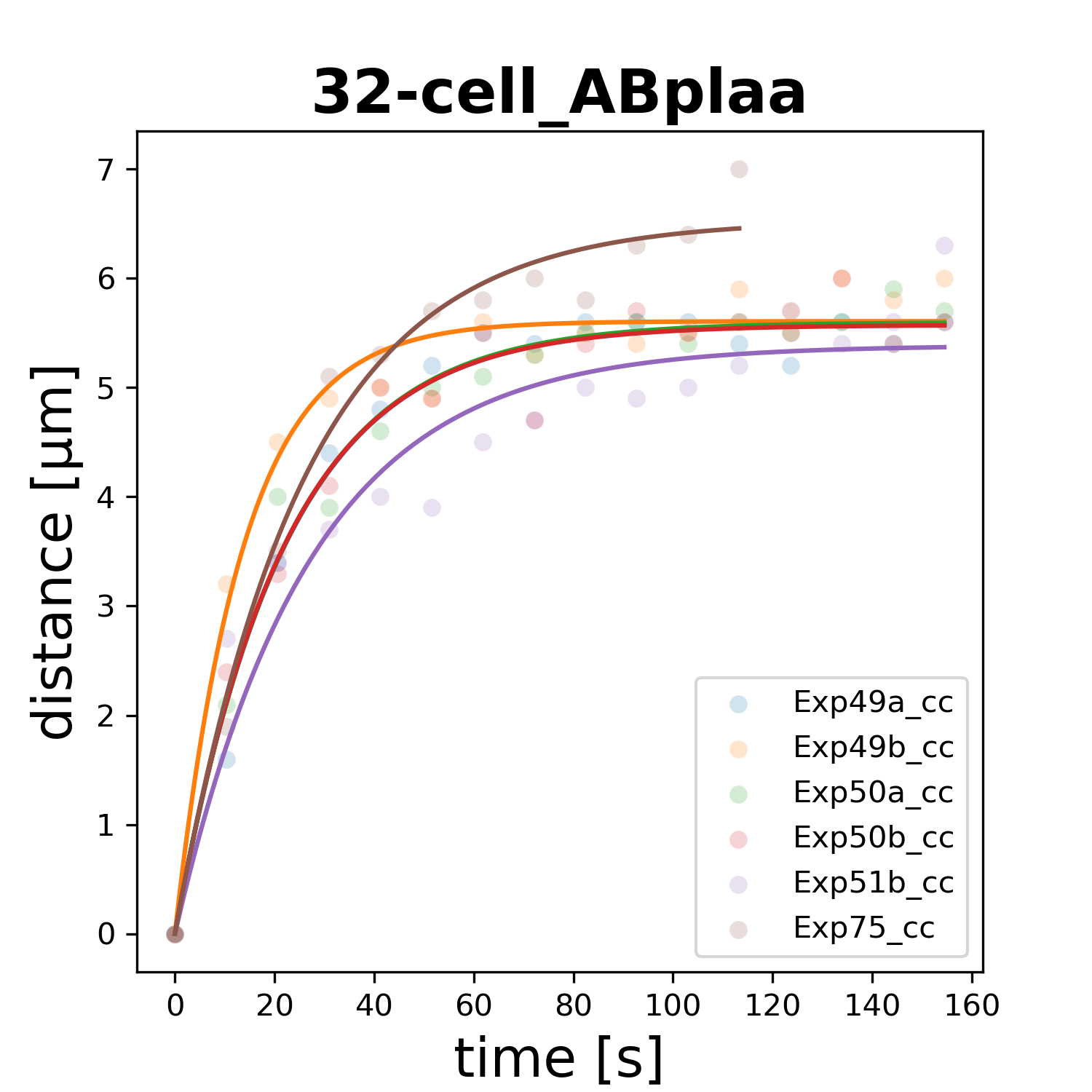

Supplement: Supplement 2 [file media-2.zip › Supplementary Material/ani2(RNAi)_chromosome_to_chromosome_distance/32-cell_ABplaa.png]

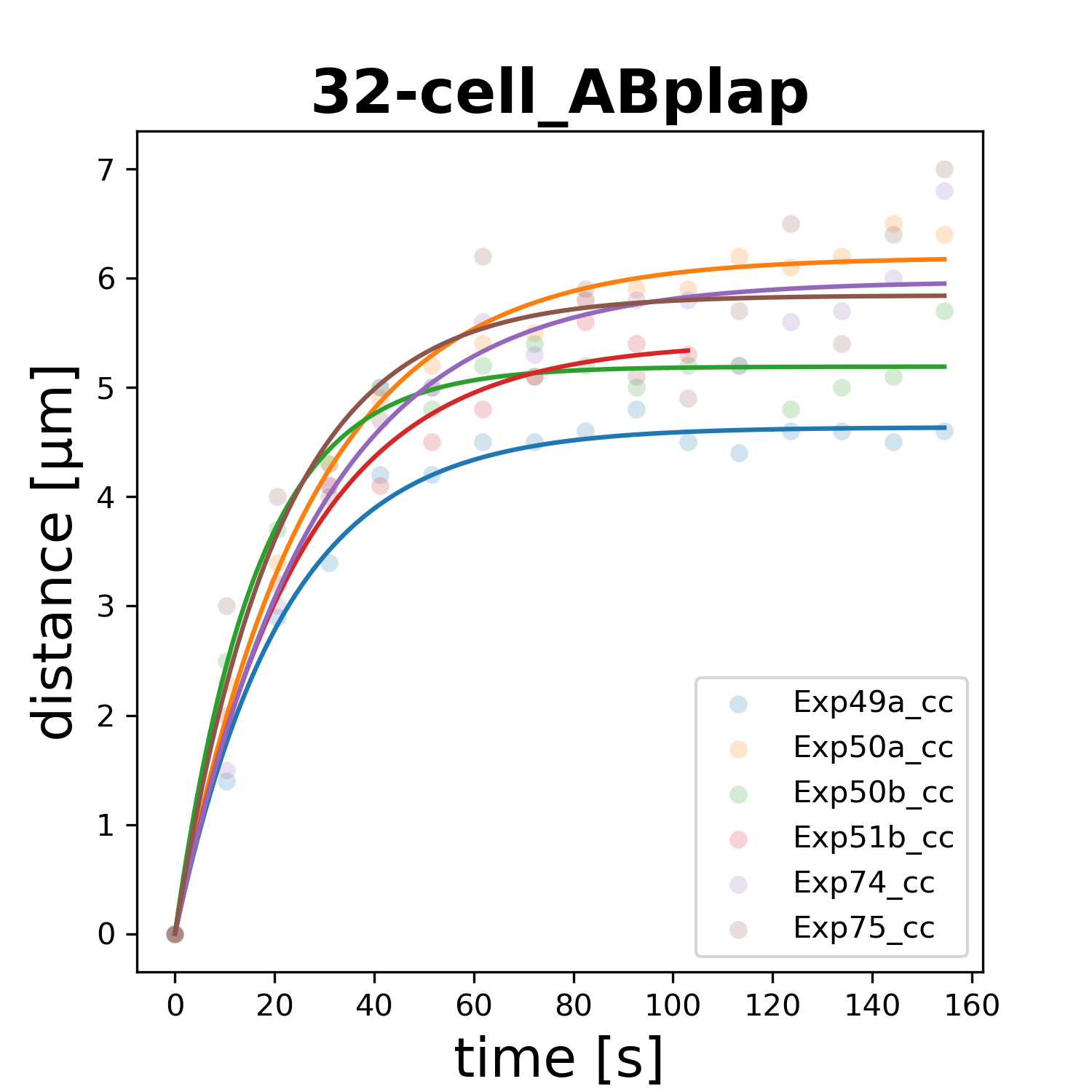

Supplement: Supplement 2 [file media-2.zip › Supplementary Material/ani2(RNAi)_chromosome_to_chromosome_distance/32-cell_ABplap.png]

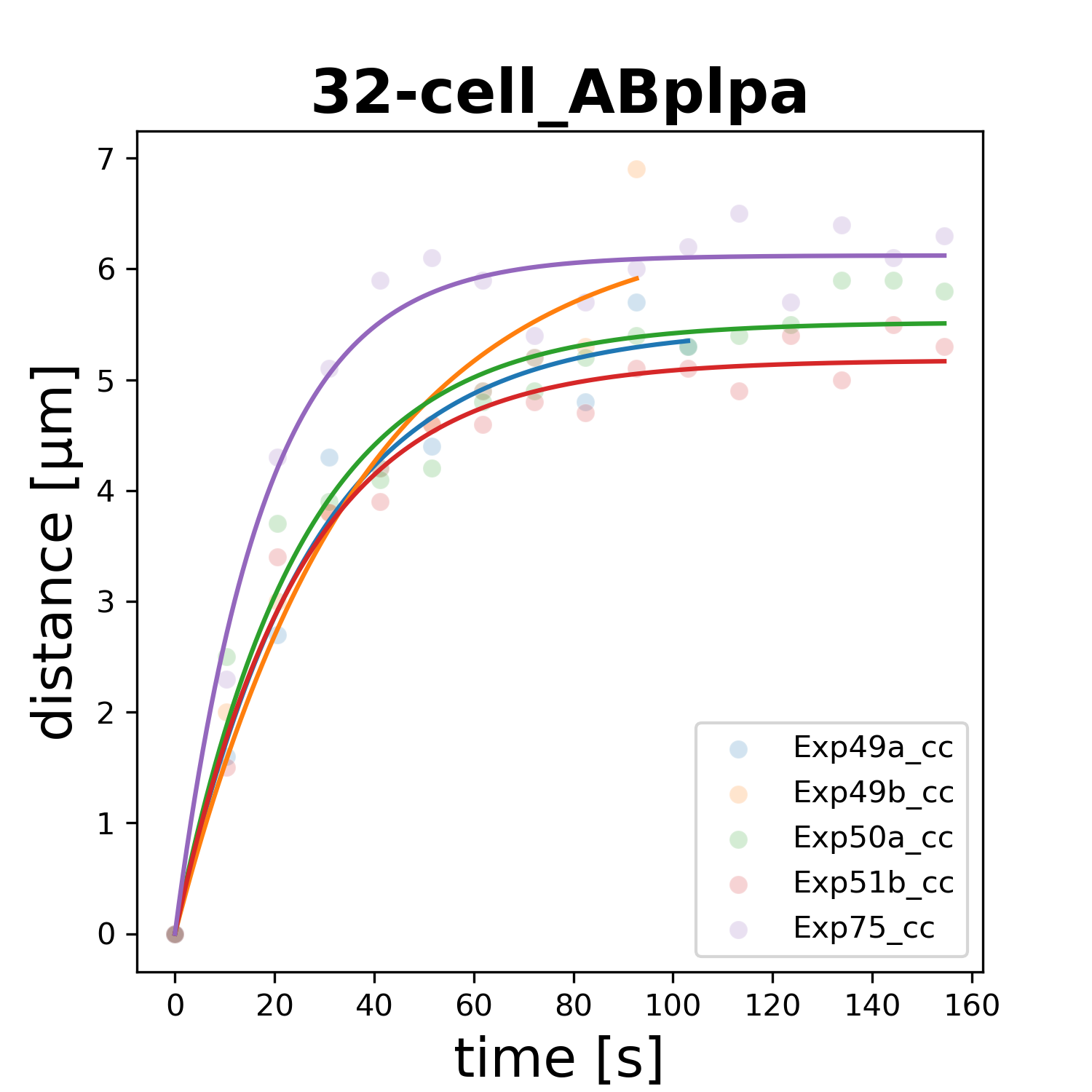

Supplement: Supplement 2 [file media-2.zip › Supplementary Material/ani2(RNAi)_chromosome_to_chromosome_distance/32-cell_ABplpa.png]

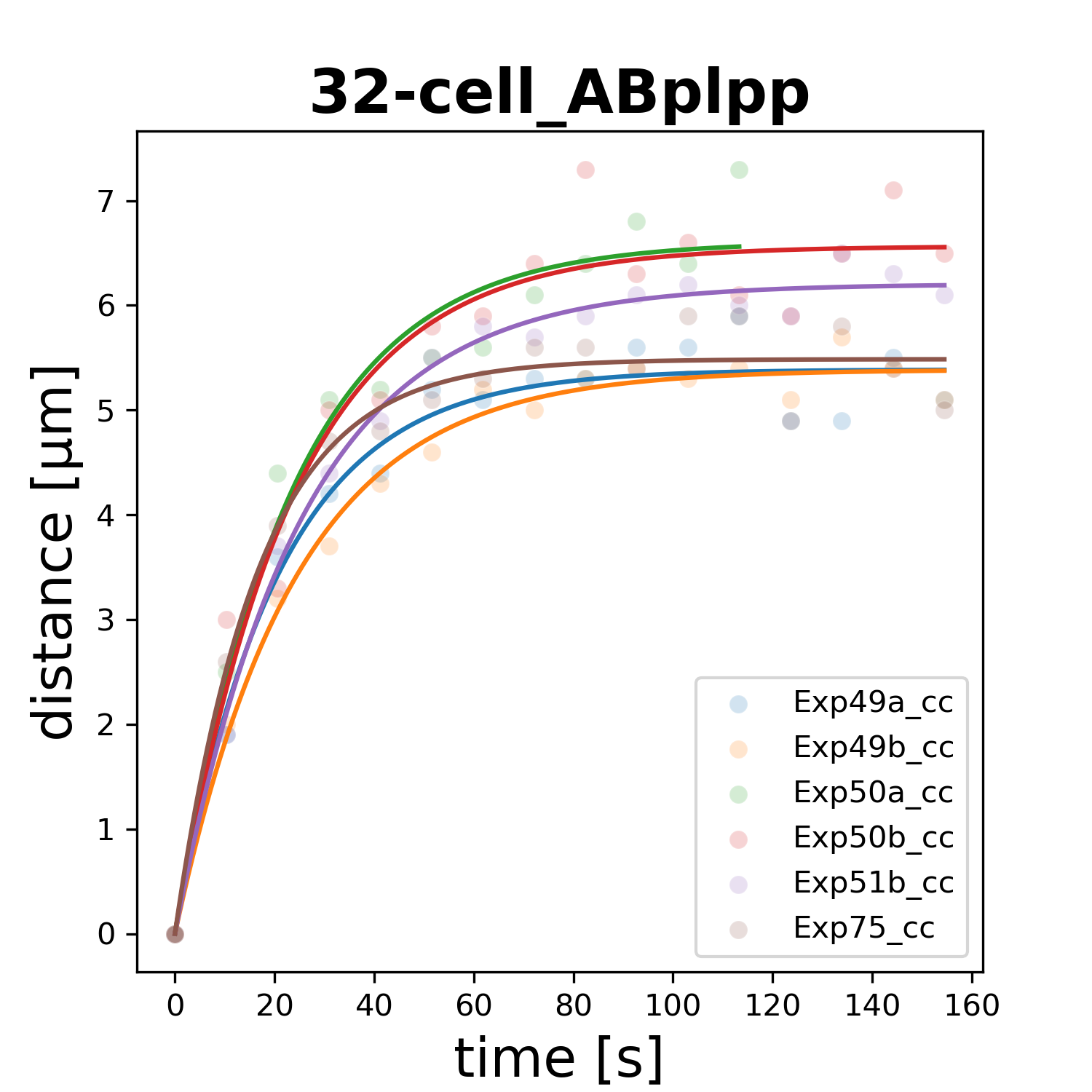

Supplement: Supplement 2 [file media-2.zip › Supplementary Material/ani2(RNAi)_chromosome_to_chromosome_distance/32-cell_ABplpp.png]

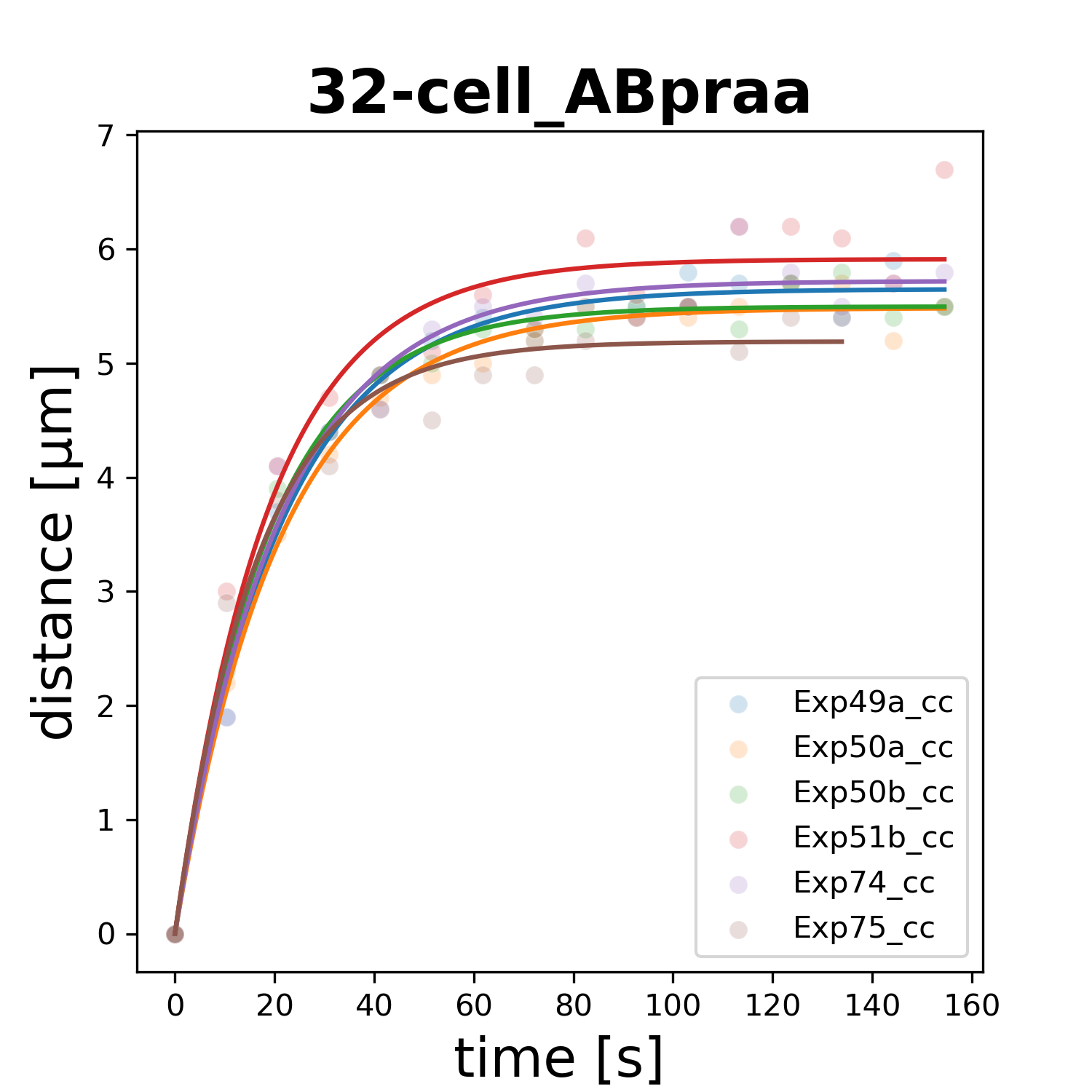

Supplement: Supplement 2 [file media-2.zip › Supplementary Material/ani2(RNAi)_chromosome_to_chromosome_distance/32-cell_ABpraa.png]

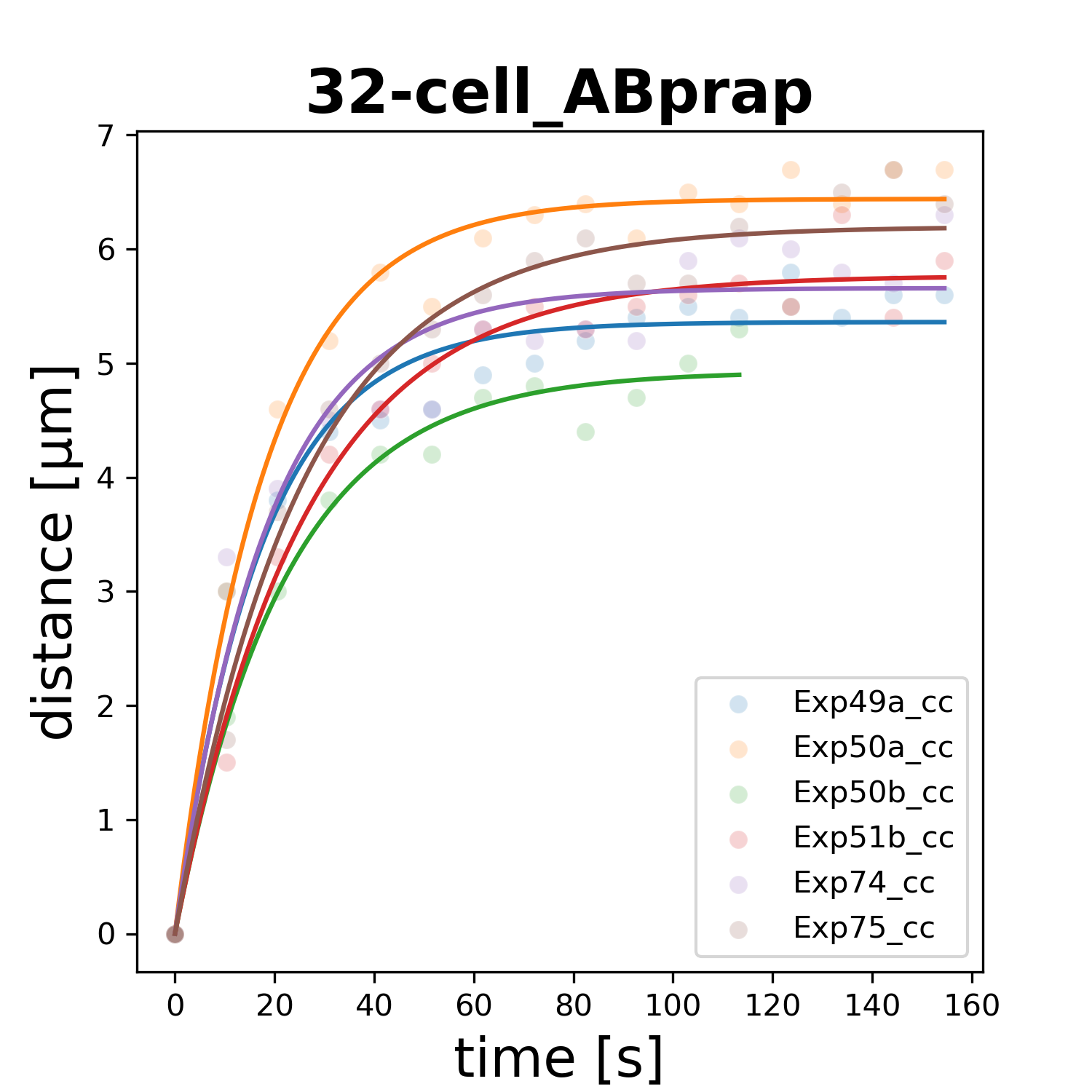

Supplement: Supplement 2 [file media-2.zip › Supplementary Material/ani2(RNAi)_chromosome_to_chromosome_distance/32-cell_ABprap.png]

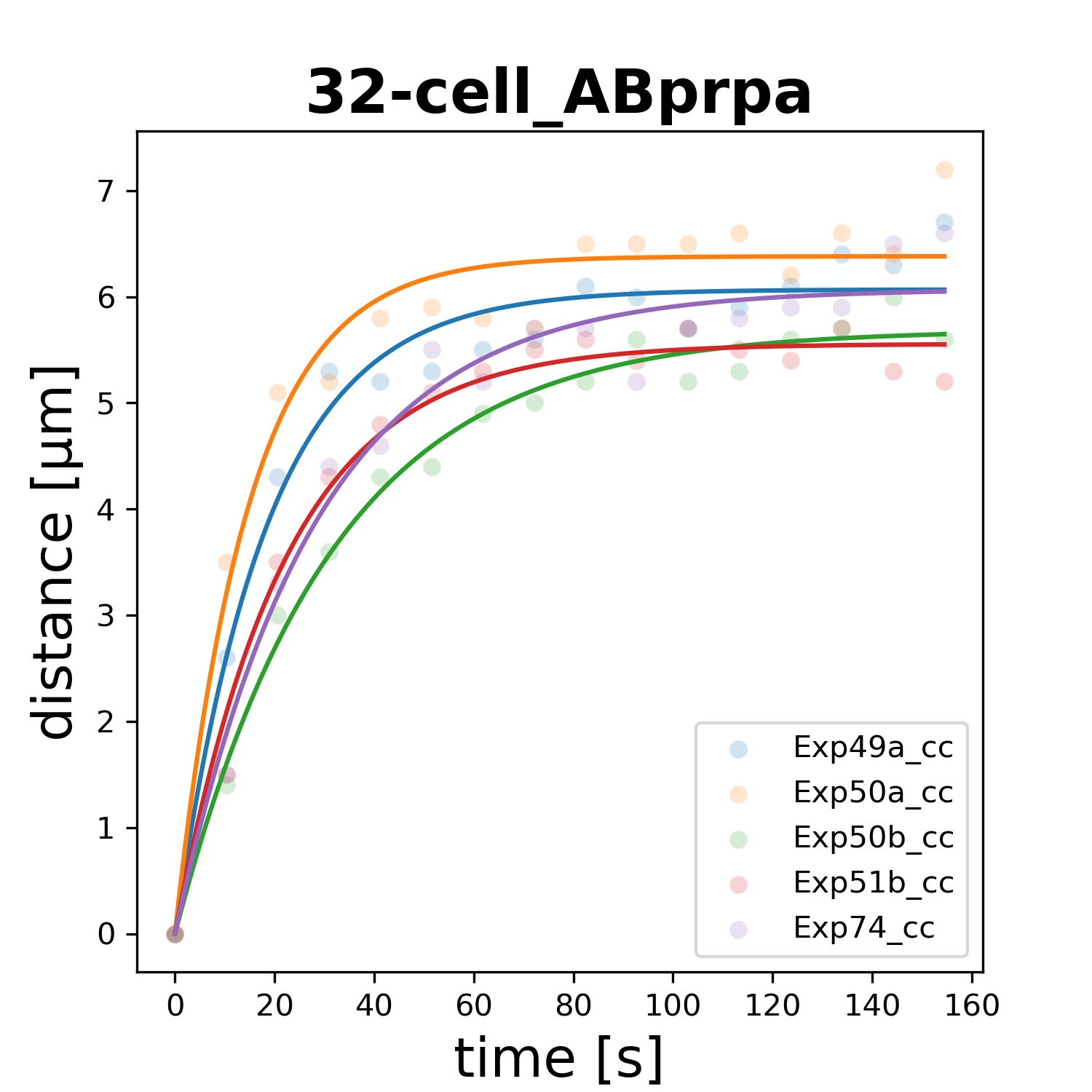

Supplement: Supplement 2 [file media-2.zip › Supplementary Material/ani2(RNAi)_chromosome_to_chromosome_distance/32-cell_ABprpa.png]

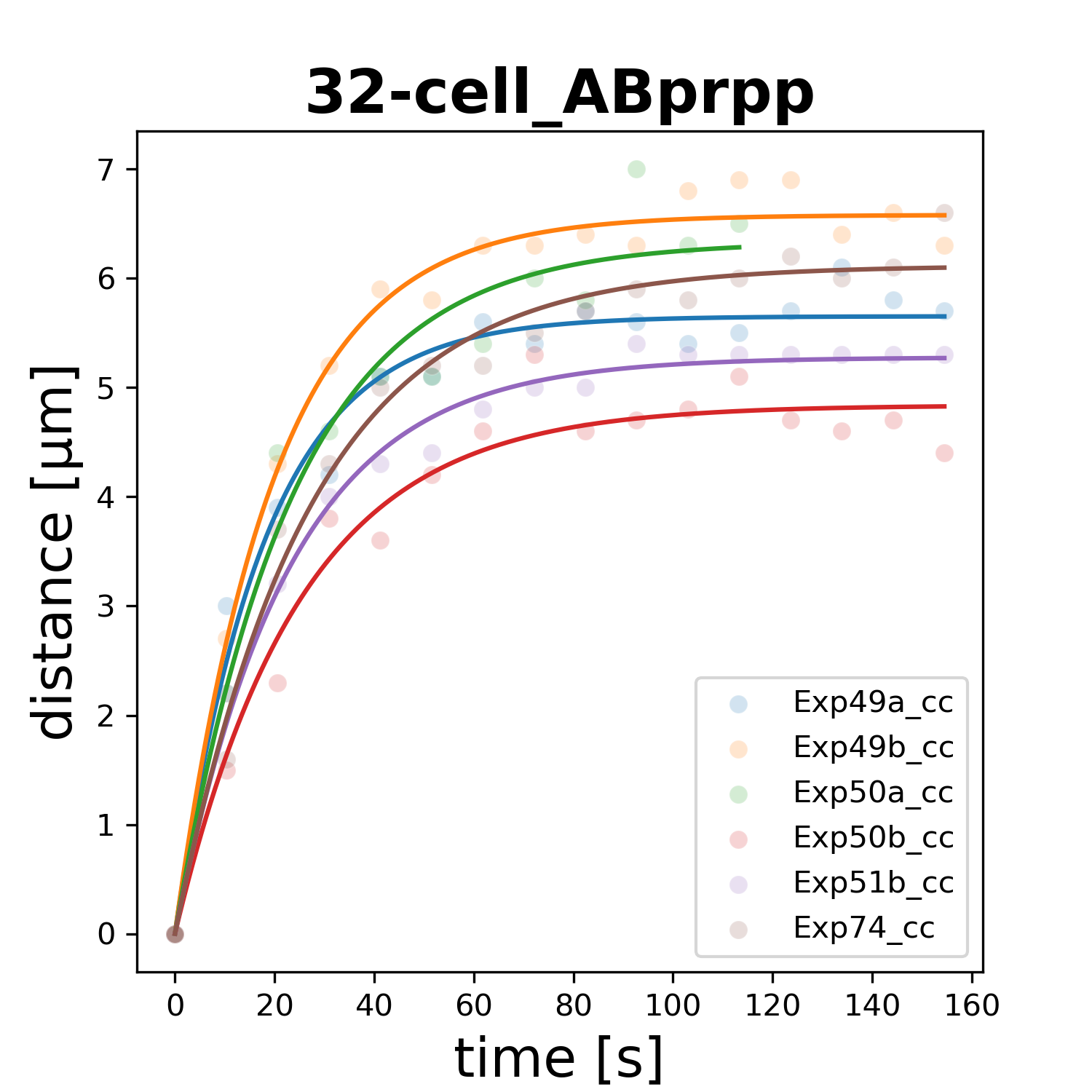

Supplement: Supplement 2 [file media-2.zip › Supplementary Material/ani2(RNAi)_chromosome_to_chromosome_distance/32-cell_ABprpp.png]

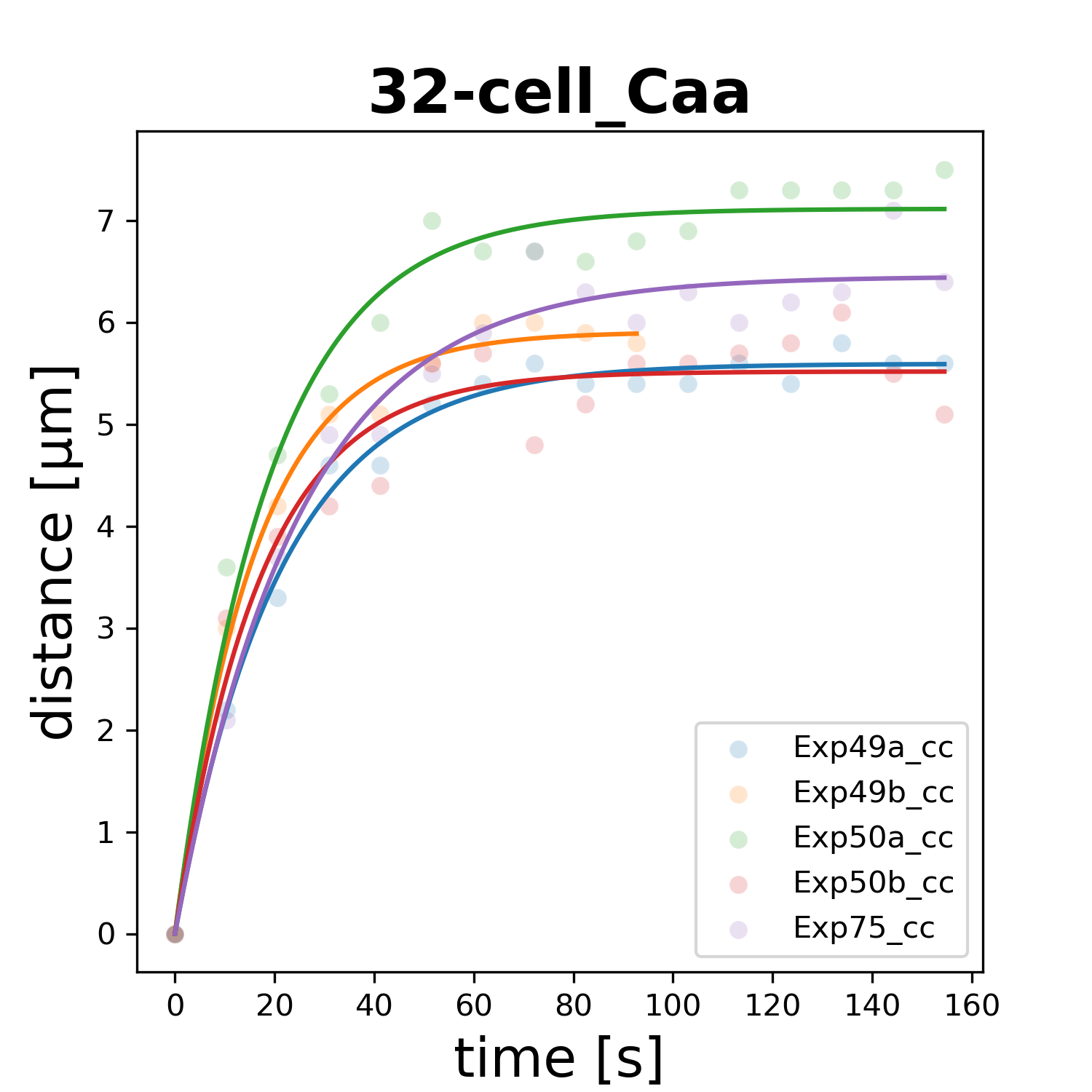

Supplement: Supplement 2 [file media-2.zip › Supplementary Material/ani2(RNAi)_chromosome_to_chromosome_distance/32-cell_Caa.png]

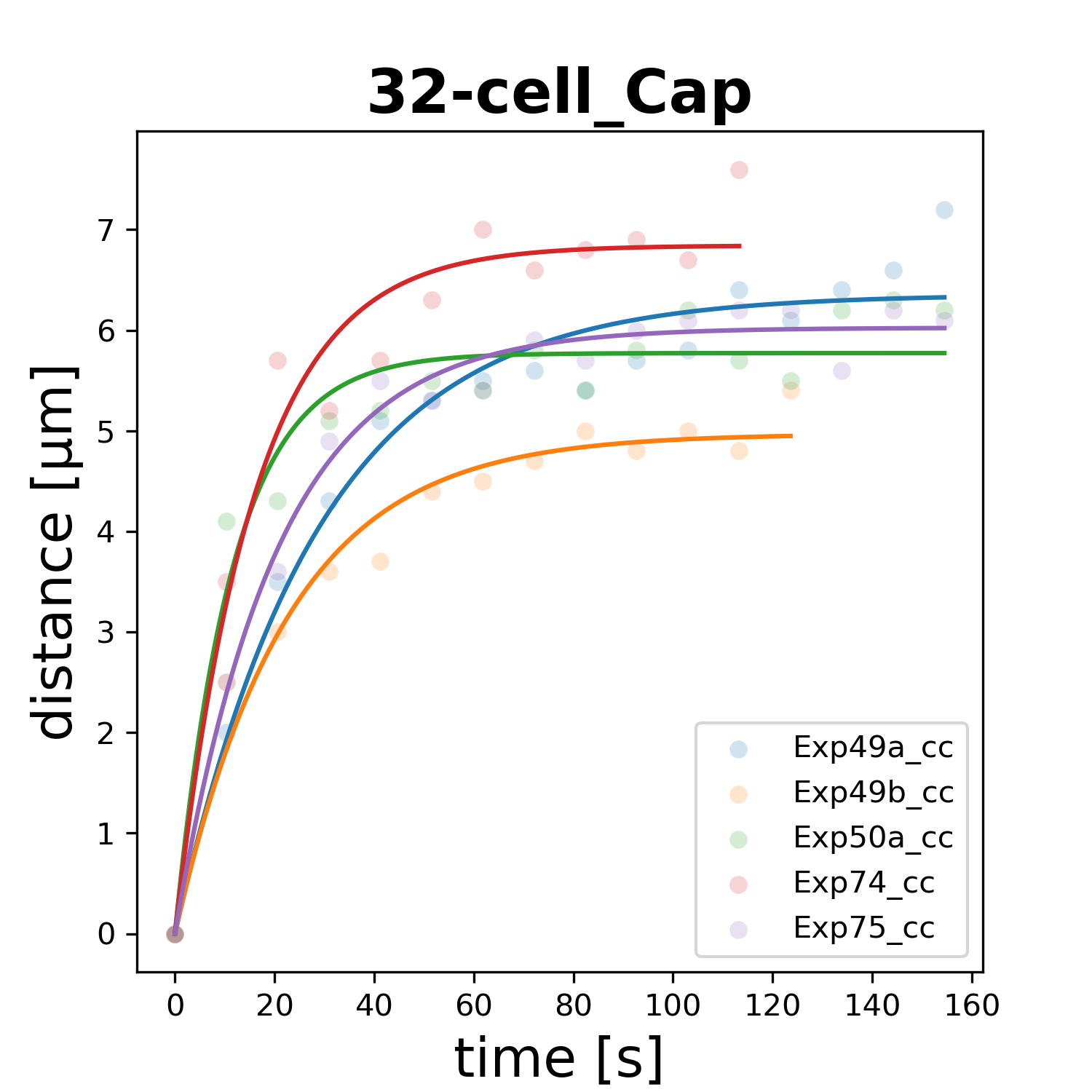

Supplement: Supplement 2 [file media-2.zip › Supplementary Material/ani2(RNAi)_chromosome_to_chromosome_distance/32-cell_Cap.png]

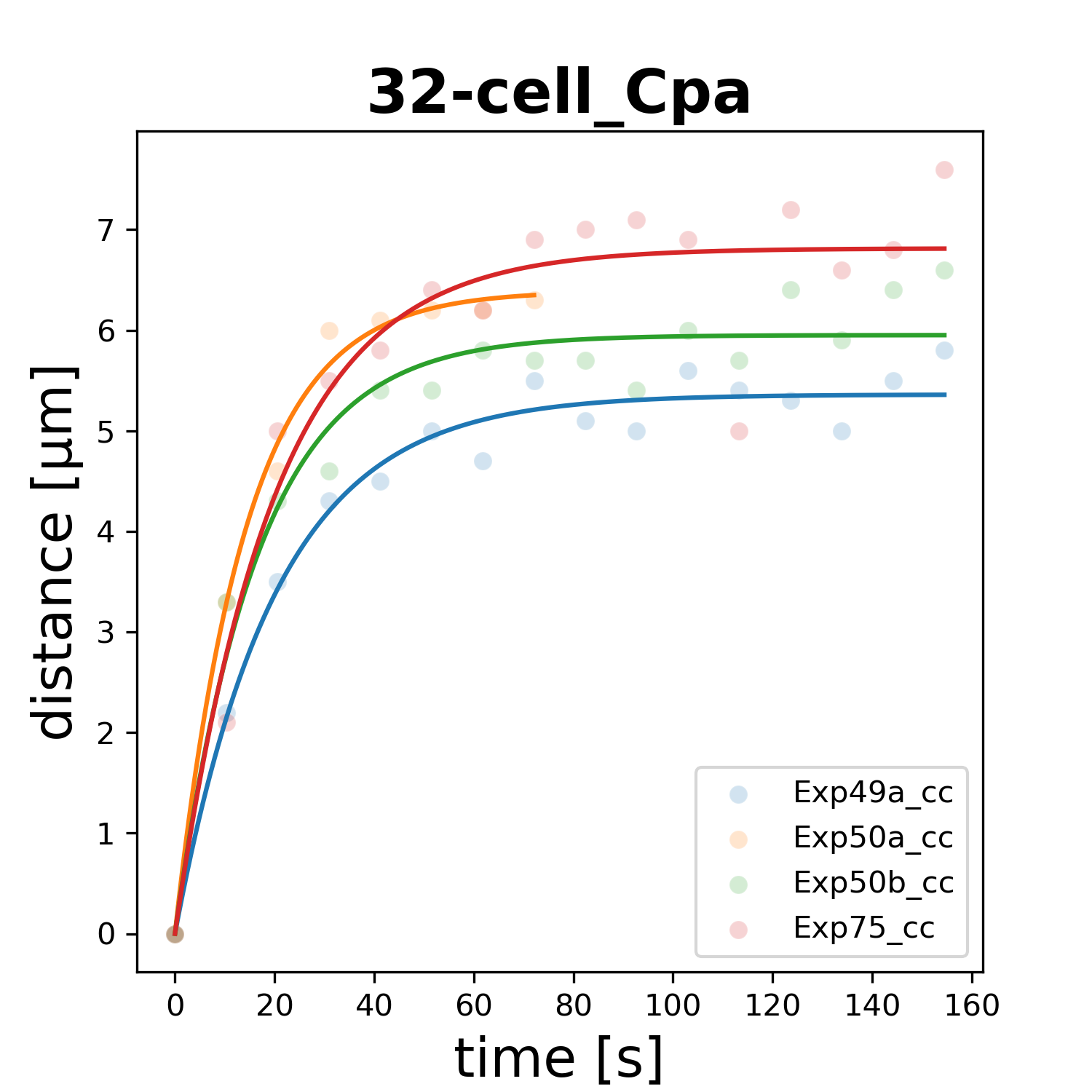

Supplement: Supplement 2 [file media-2.zip › Supplementary Material/ani2(RNAi)_chromosome_to_chromosome_distance/32-cell_Cpa.png]

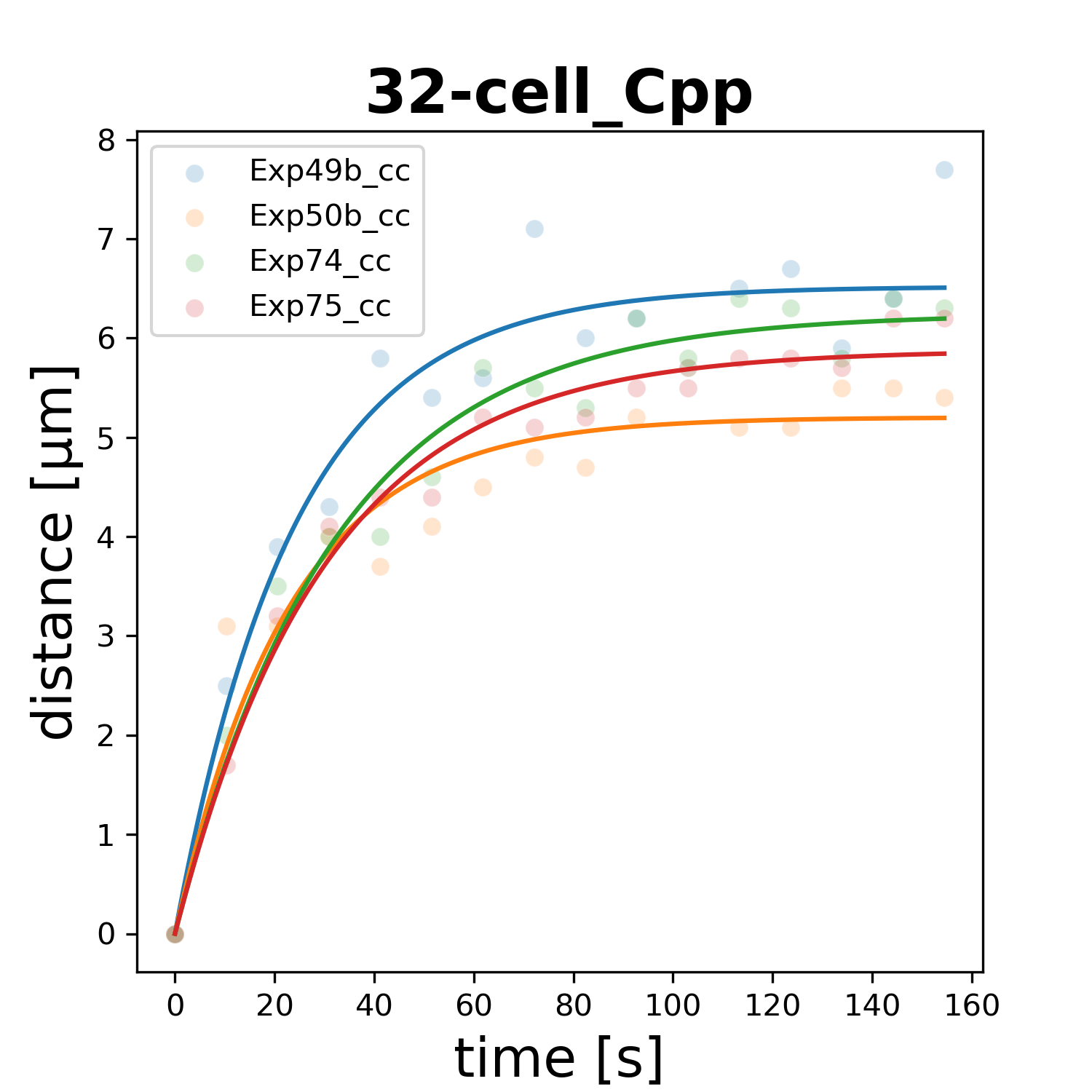

Supplement: Supplement 2 [file media-2.zip › Supplementary Material/ani2(RNAi)_chromosome_to_chromosome_distance/32-cell_Cpp.png]

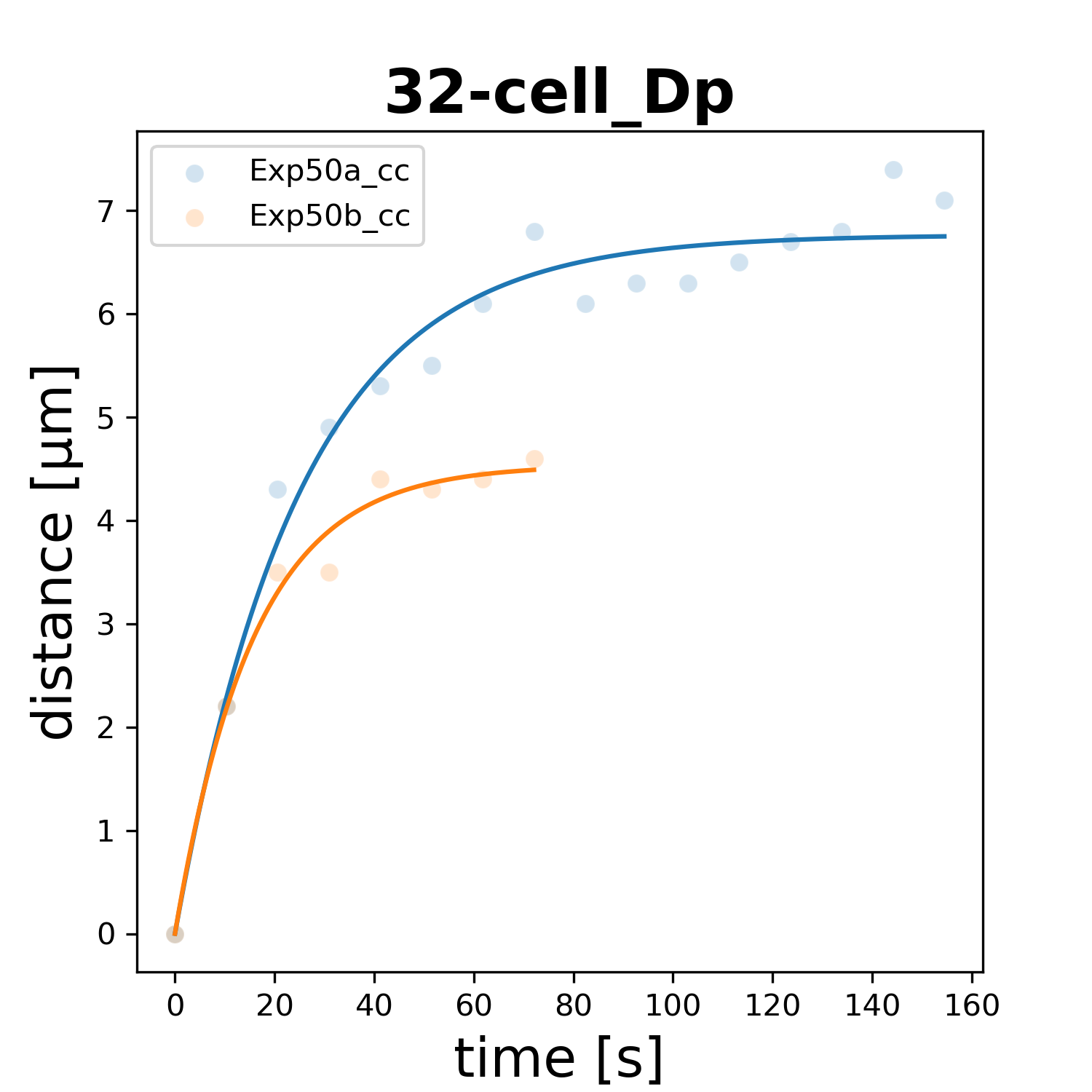

Supplement: Supplement 2 [file media-2.zip › Supplementary Material/ani2(RNAi)_chromosome_to_chromosome_distance/32-cell_Dp.png]

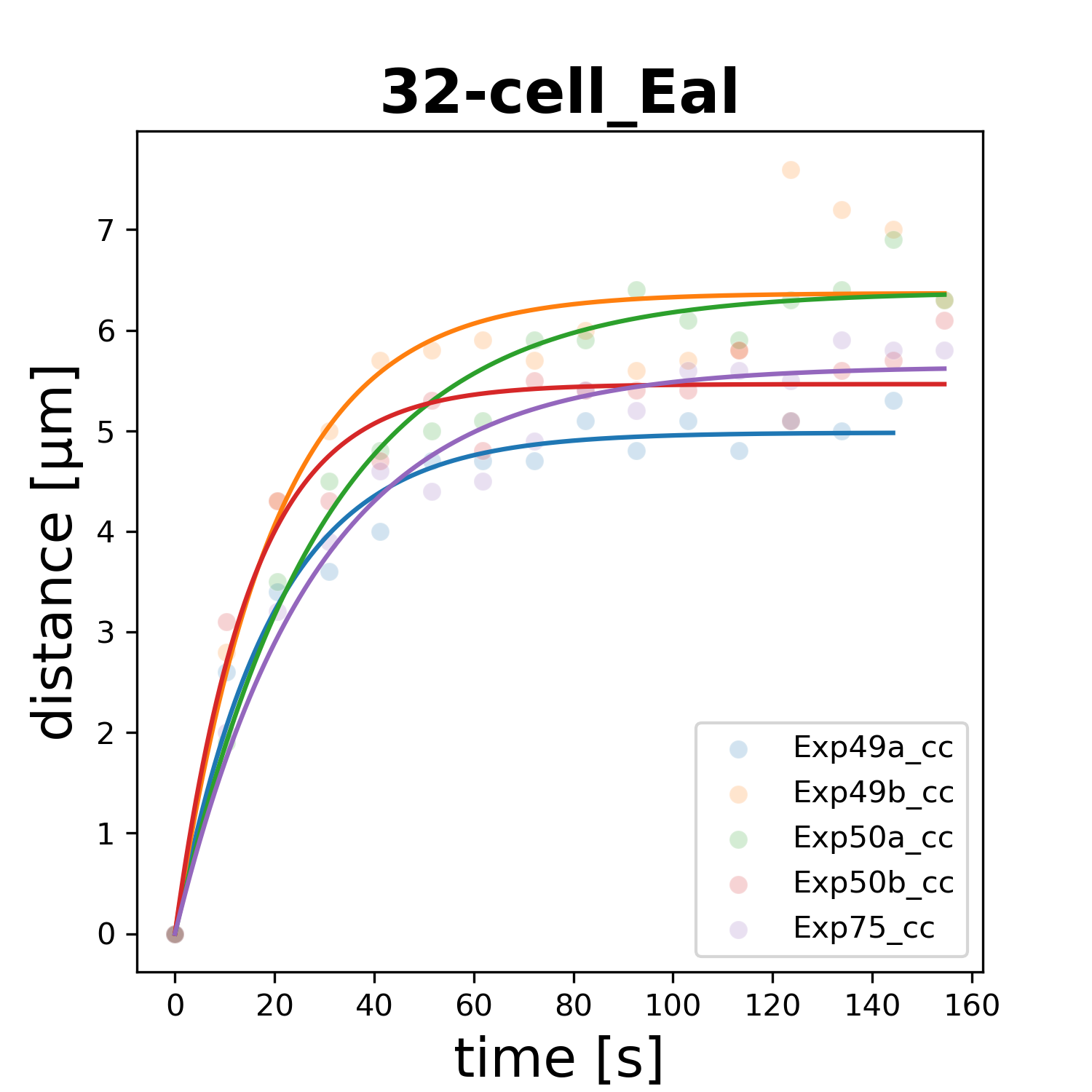

Supplement: Supplement 2 [file media-2.zip › Supplementary Material/ani2(RNAi)_chromosome_to_chromosome_distance/32-cell_Eal.png]

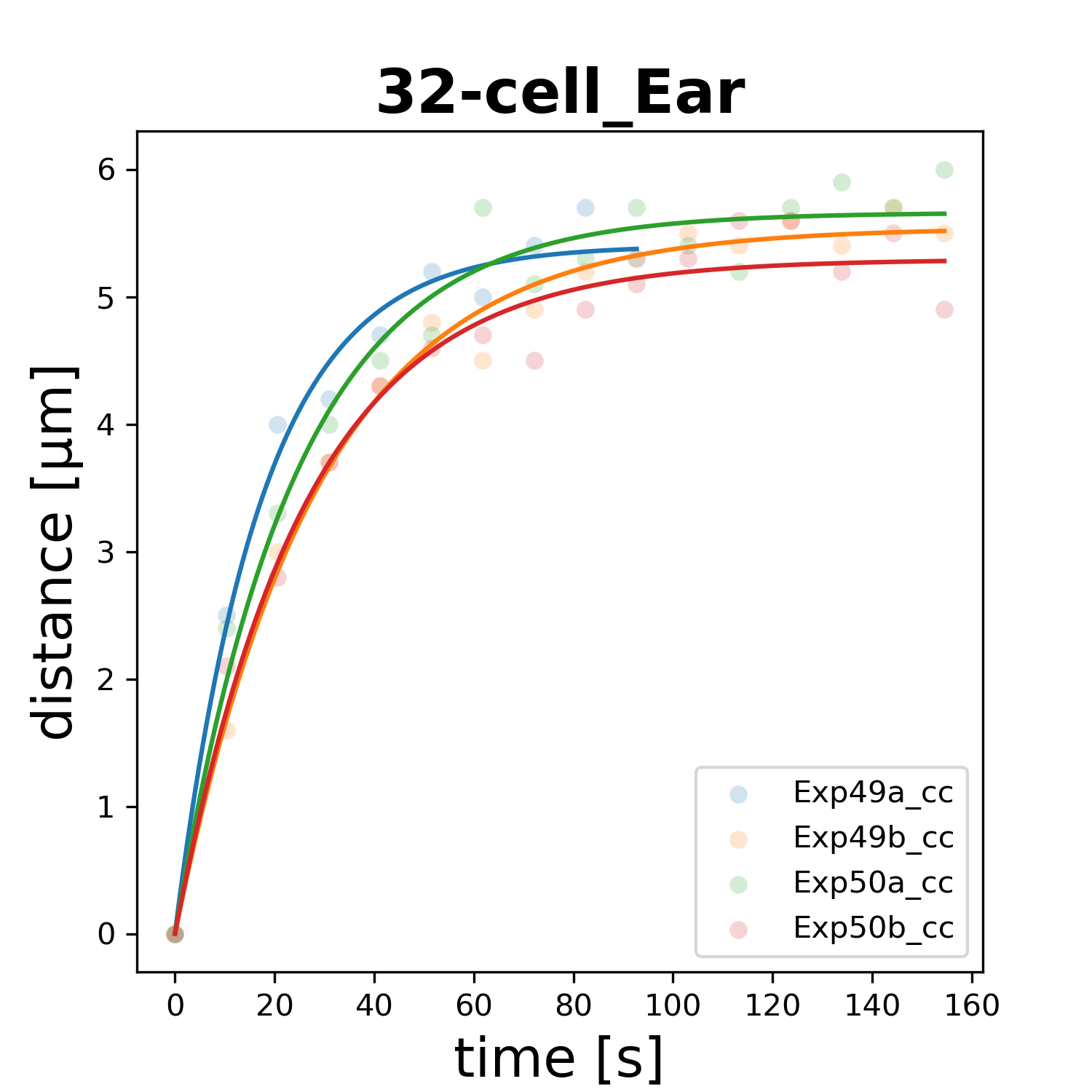

Supplement: Supplement 2 [file media-2.zip › Supplementary Material/ani2(RNAi)_chromosome_to_chromosome_distance/32-cell_Ear.png]

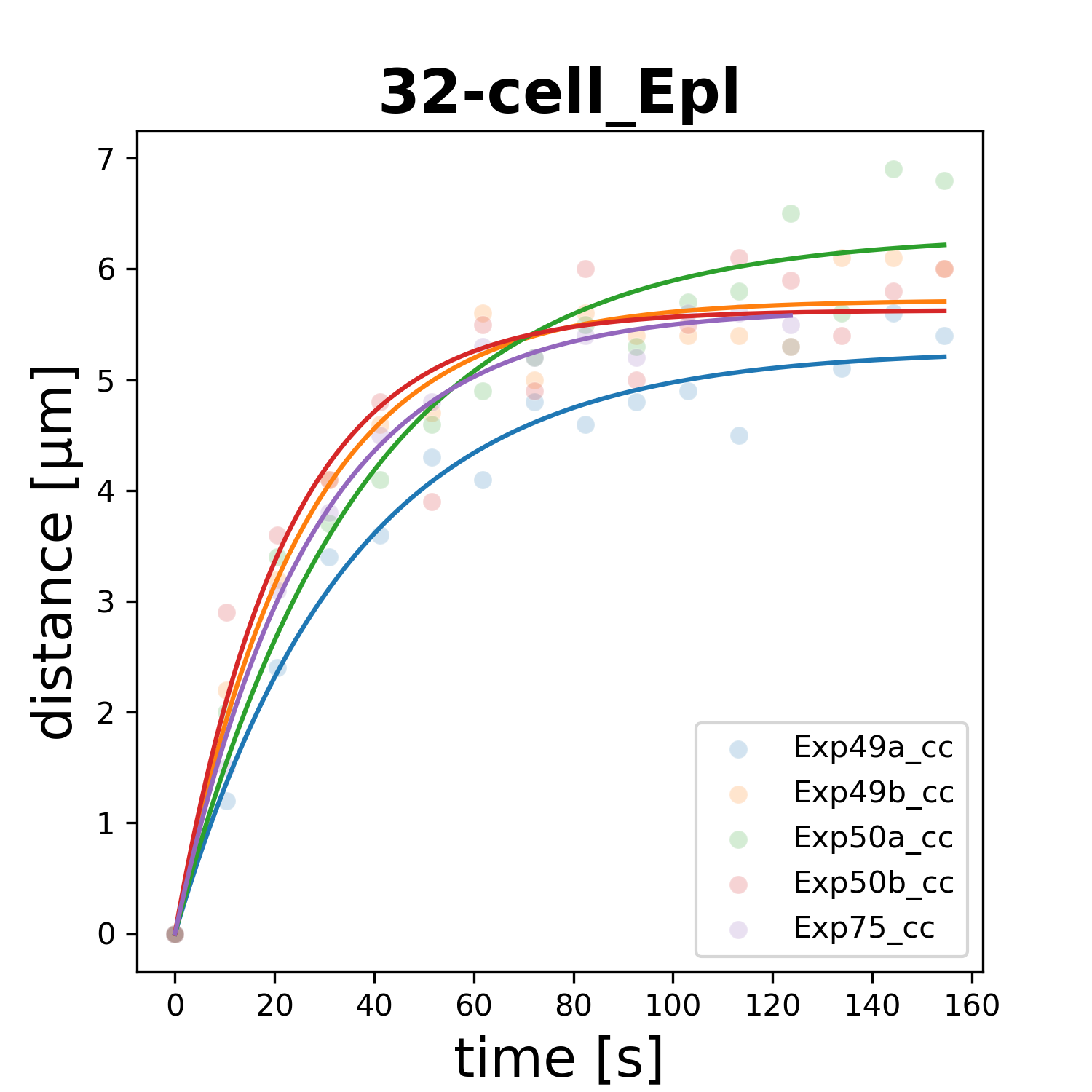

Supplement: Supplement 2 [file media-2.zip › Supplementary Material/ani2(RNAi)_chromosome_to_chromosome_distance/32-cell_Epl.png]

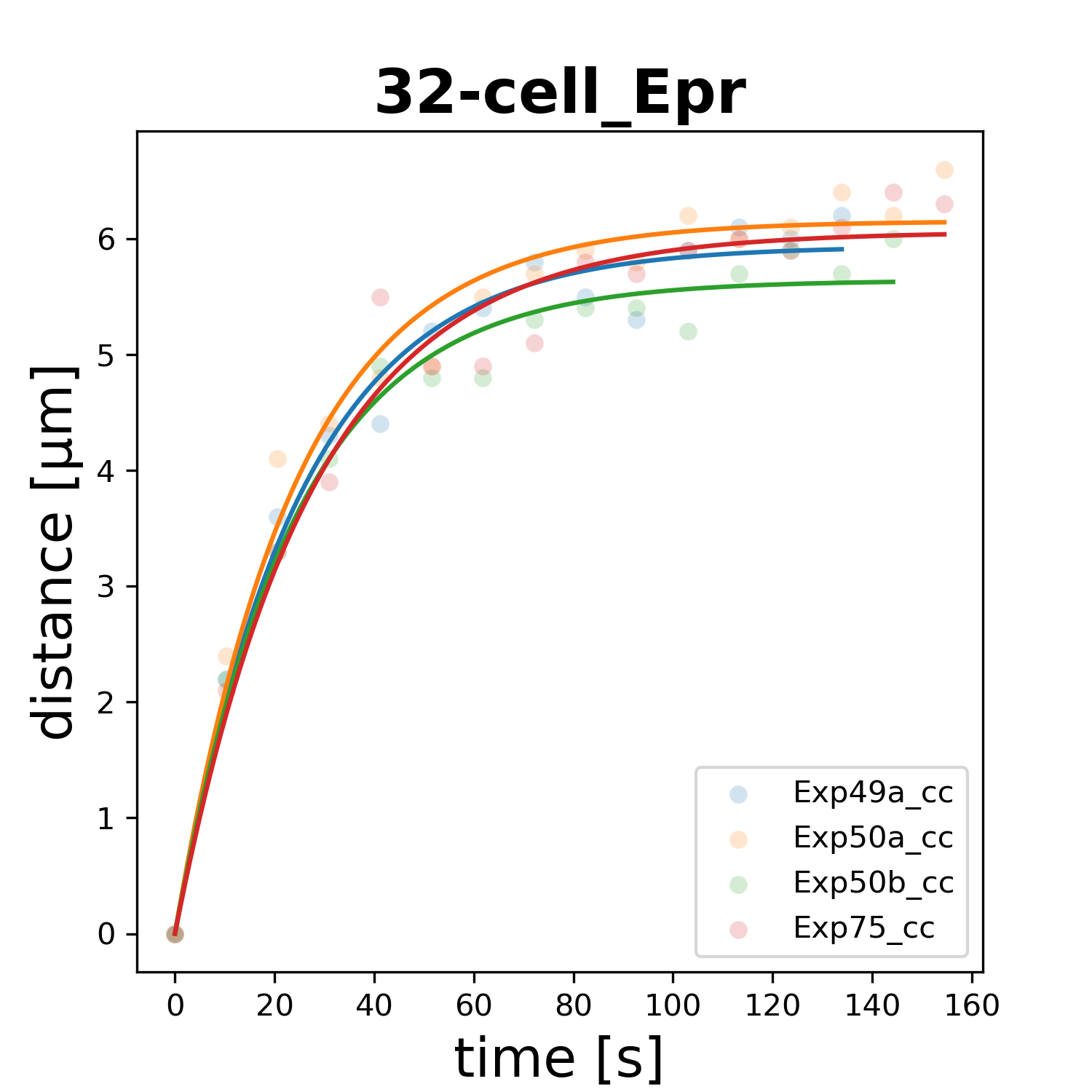

Supplement: Supplement 2 [file media-2.zip › Supplementary Material/ani2(RNAi)_chromosome_to_chromosome_distance/32-cell_Epr.png]

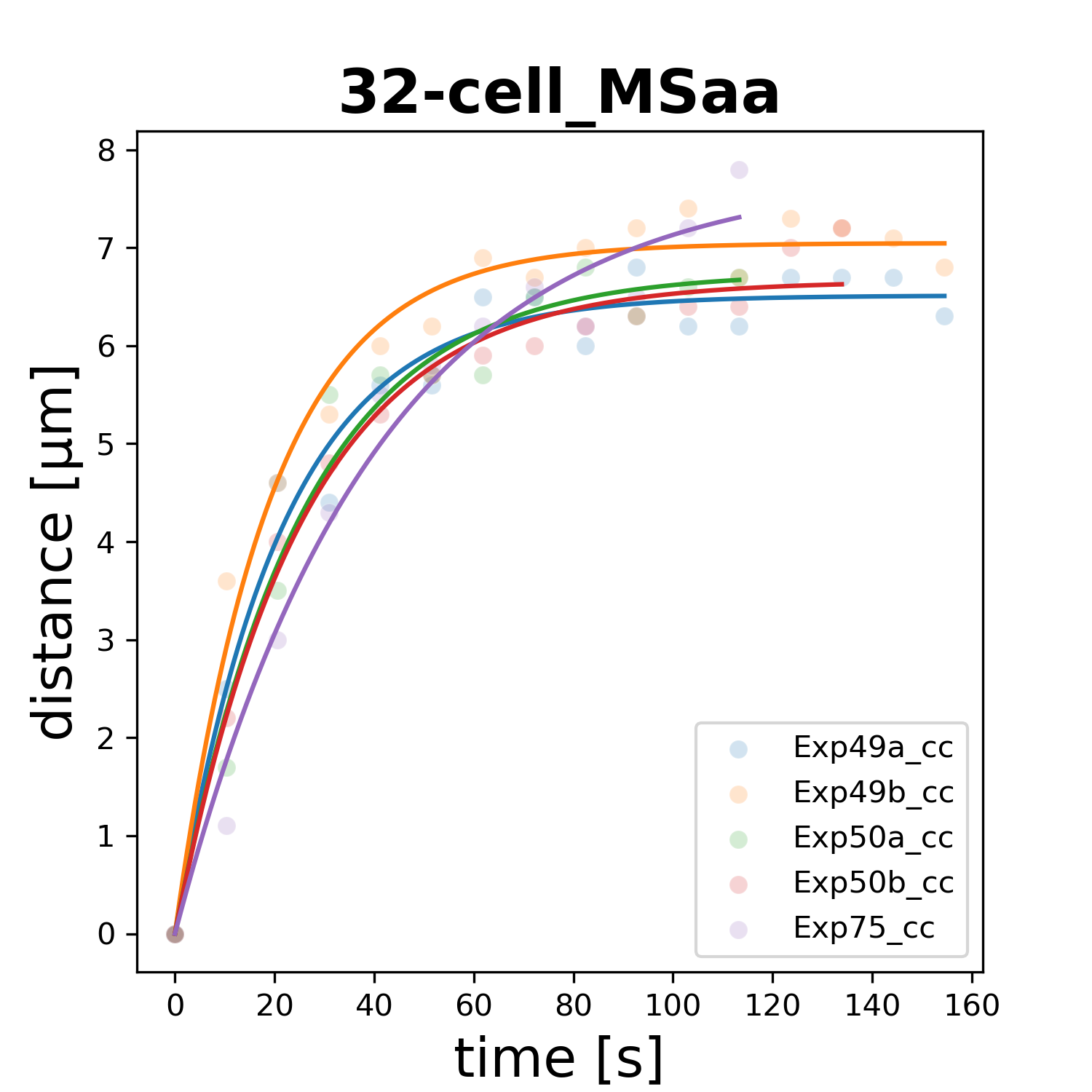

Supplement: Supplement 2 [file media-2.zip › Supplementary Material/ani2(RNAi)_chromosome_to_chromosome_distance/32-cell_MSaa.png]

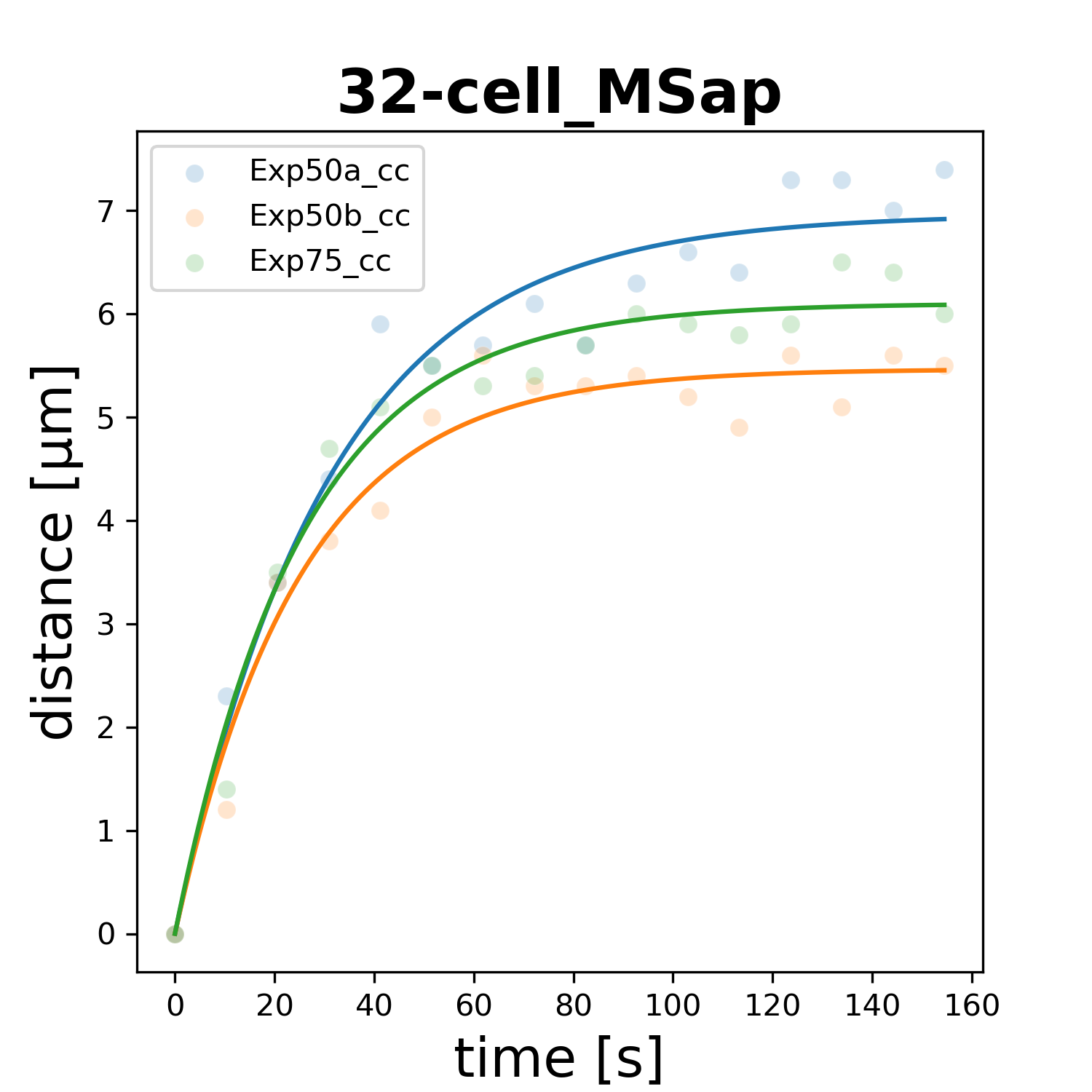

Supplement: Supplement 2 [file media-2.zip › Supplementary Material/ani2(RNAi)_chromosome_to_chromosome_distance/32-cell_MSap.png]

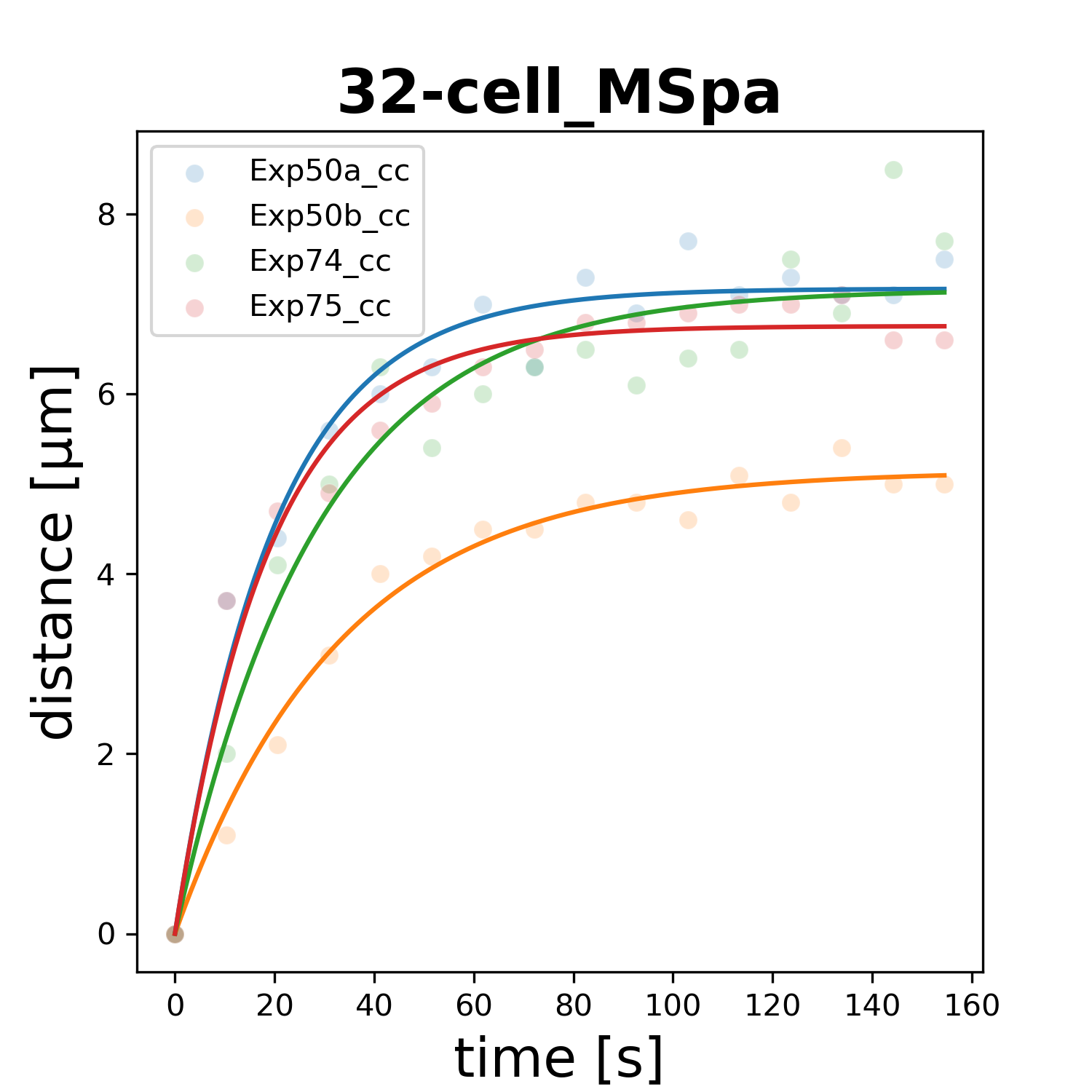

Supplement: Supplement 2 [file media-2.zip › Supplementary Material/ani2(RNAi)_chromosome_to_chromosome_distance/32-cell_MSpa.png]

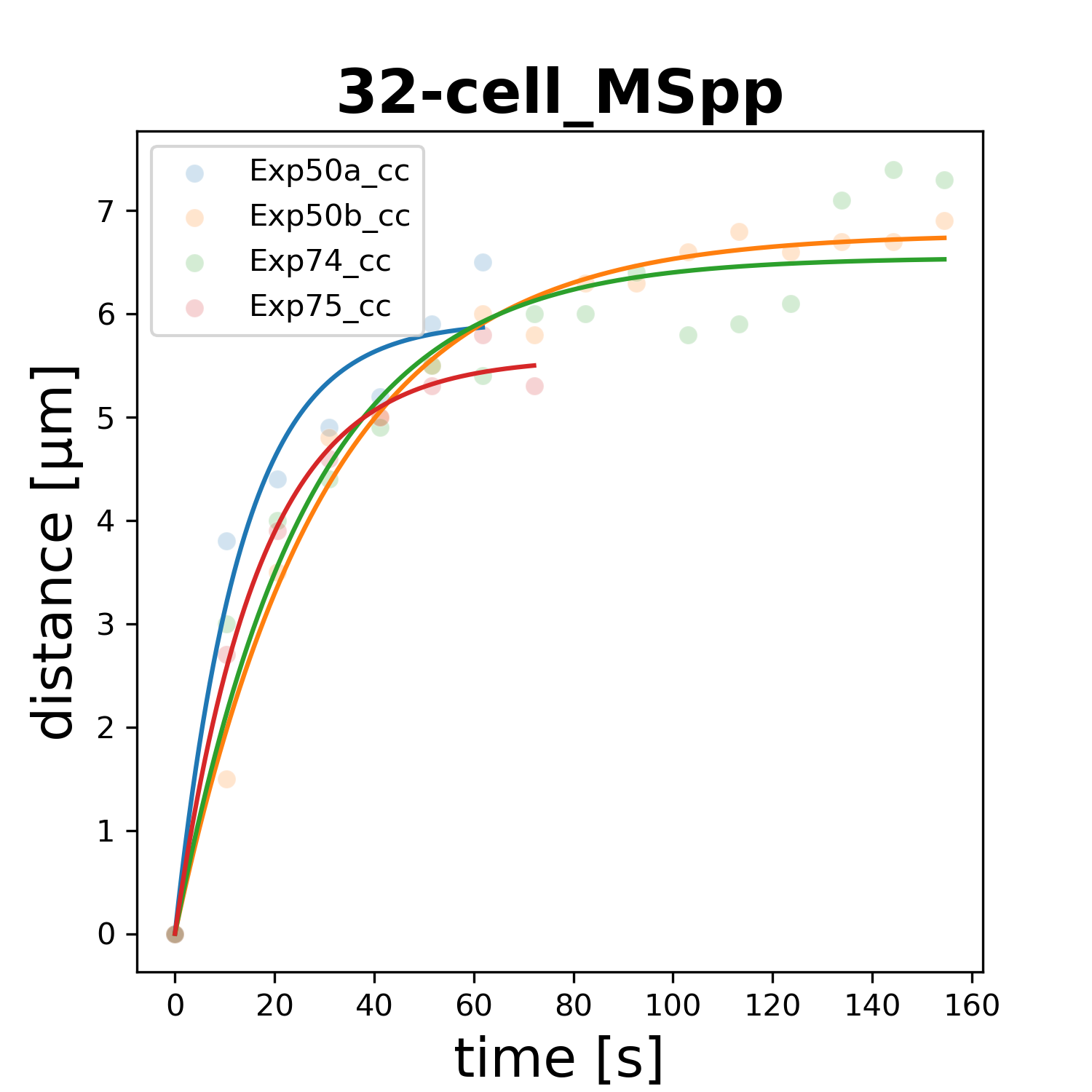

Supplement: Supplement 2 [file media-2.zip › Supplementary Material/ani2(RNAi)_chromosome_to_chromosome_distance/32-cell_MSpp.png]

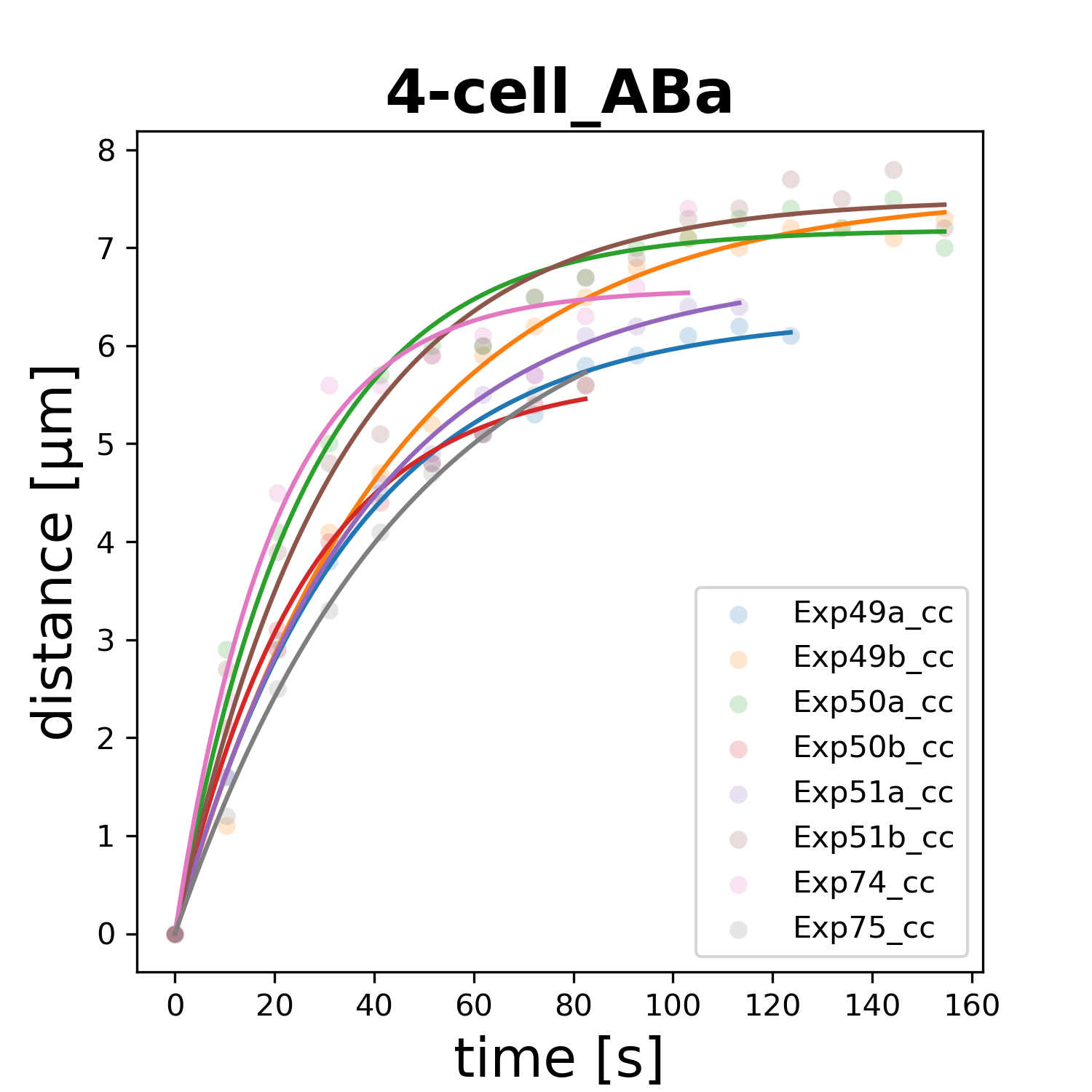

Supplement: Supplement 2 [file media-2.zip › Supplementary Material/ani2(RNAi)_chromosome_to_chromosome_distance/4-cell_ABa.png]

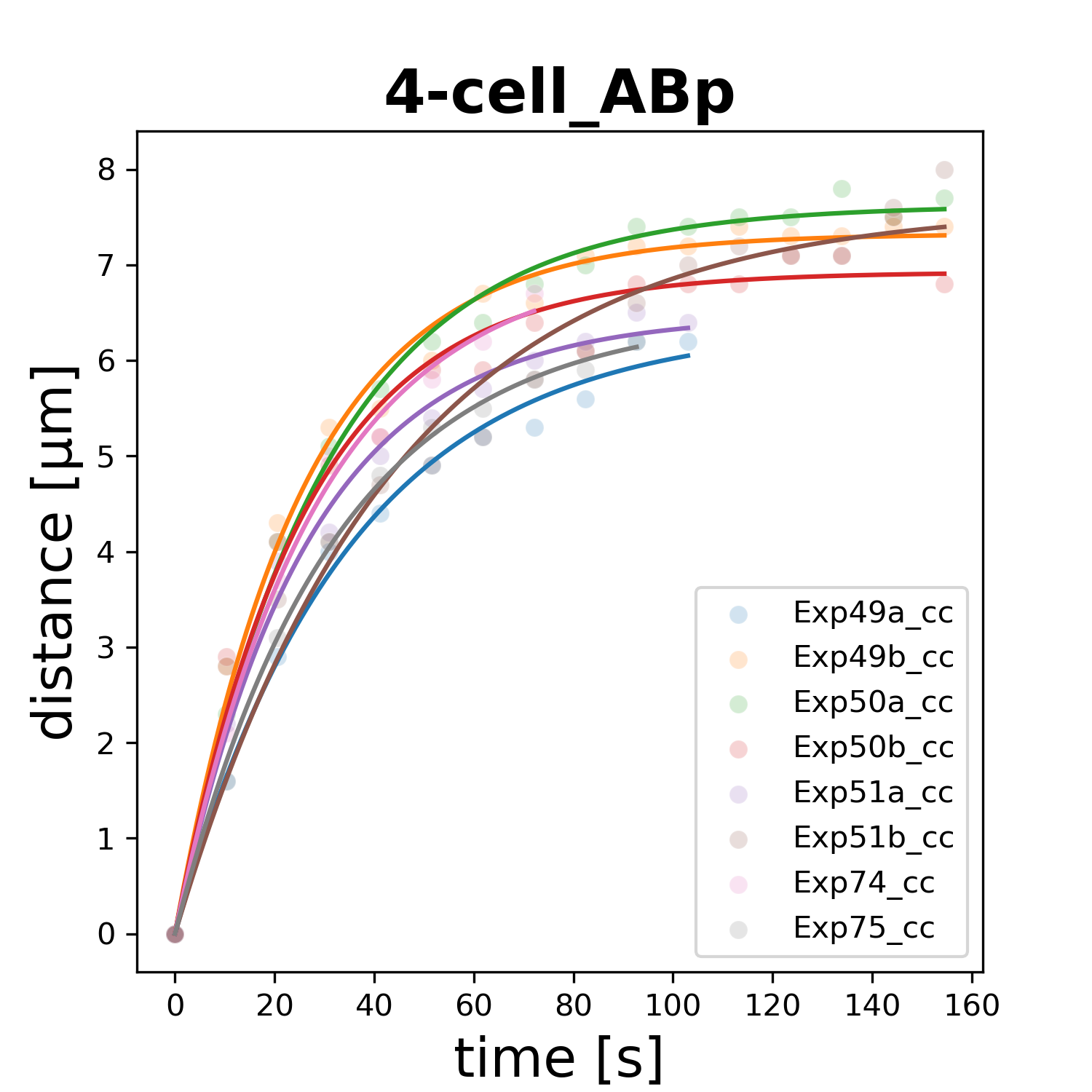

Supplement: Supplement 2 [file media-2.zip › Supplementary Material/ani2(RNAi)_chromosome_to_chromosome_distance/4-cell_ABp.png]

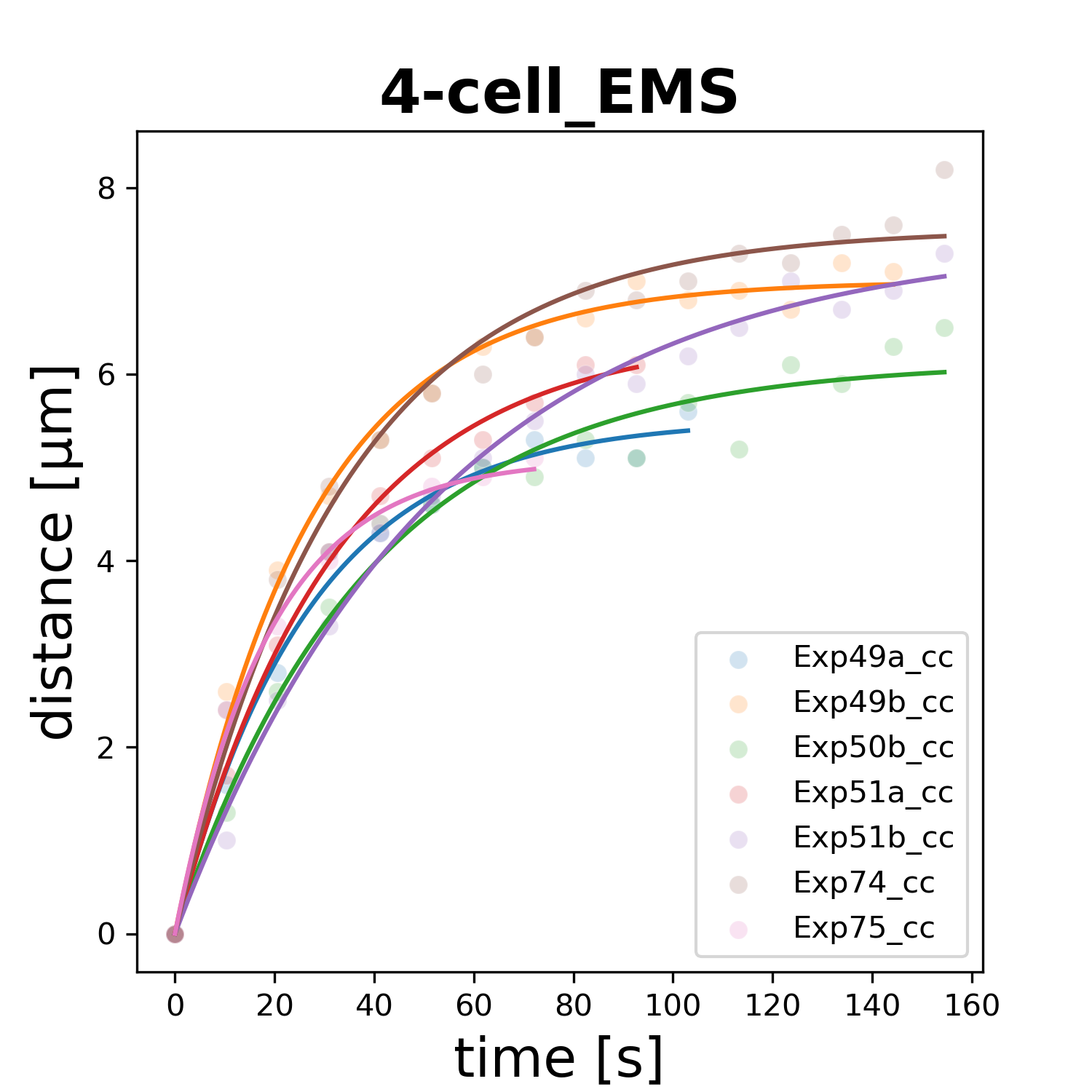

Supplement: Supplement 2 [file media-2.zip › Supplementary Material/ani2(RNAi)_chromosome_to_chromosome_distance/4-cell_EMS.png]

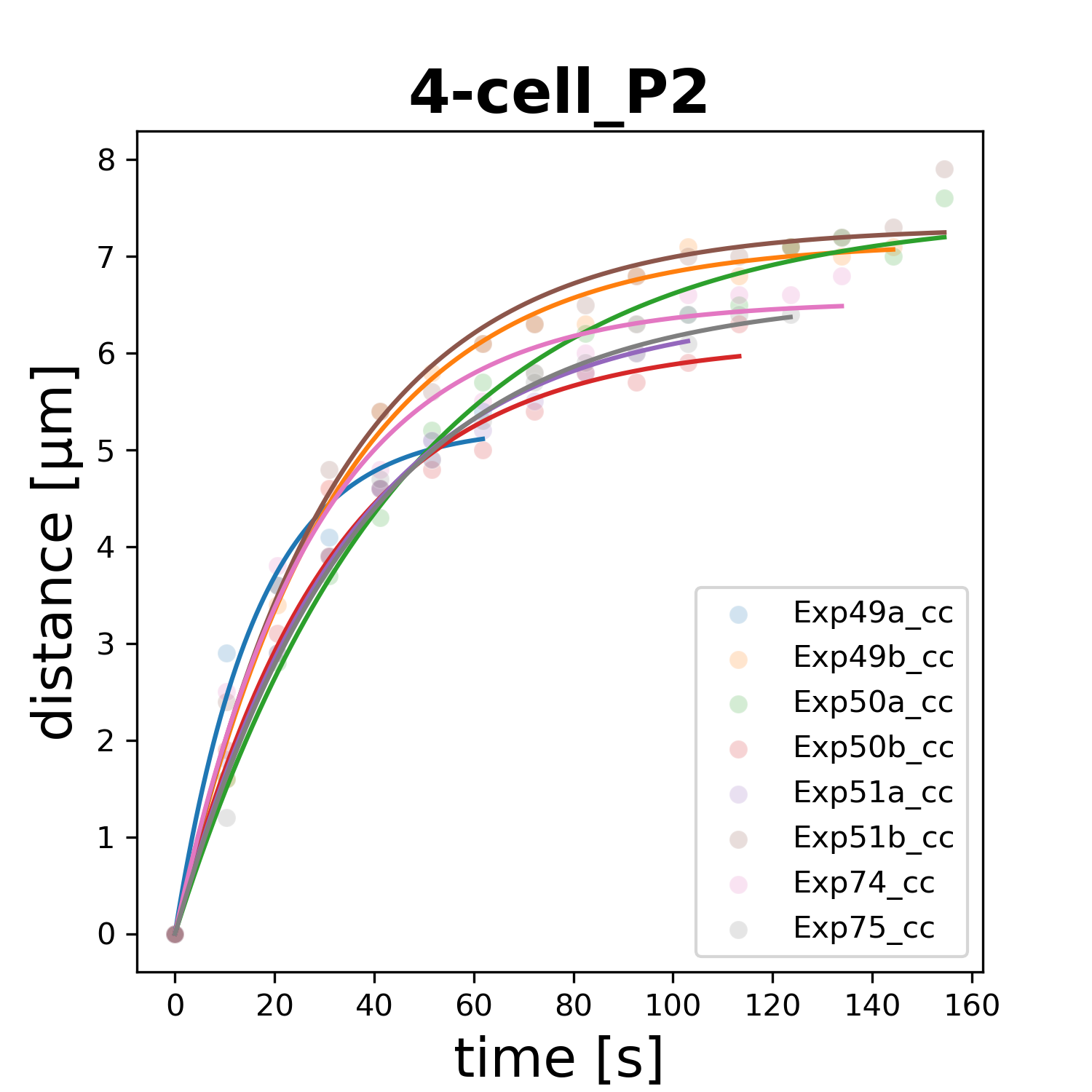

Supplement: Supplement 2 [file media-2.zip › Supplementary Material/ani2(RNAi)_chromosome_to_chromosome_distance/4-cell_P2.png]

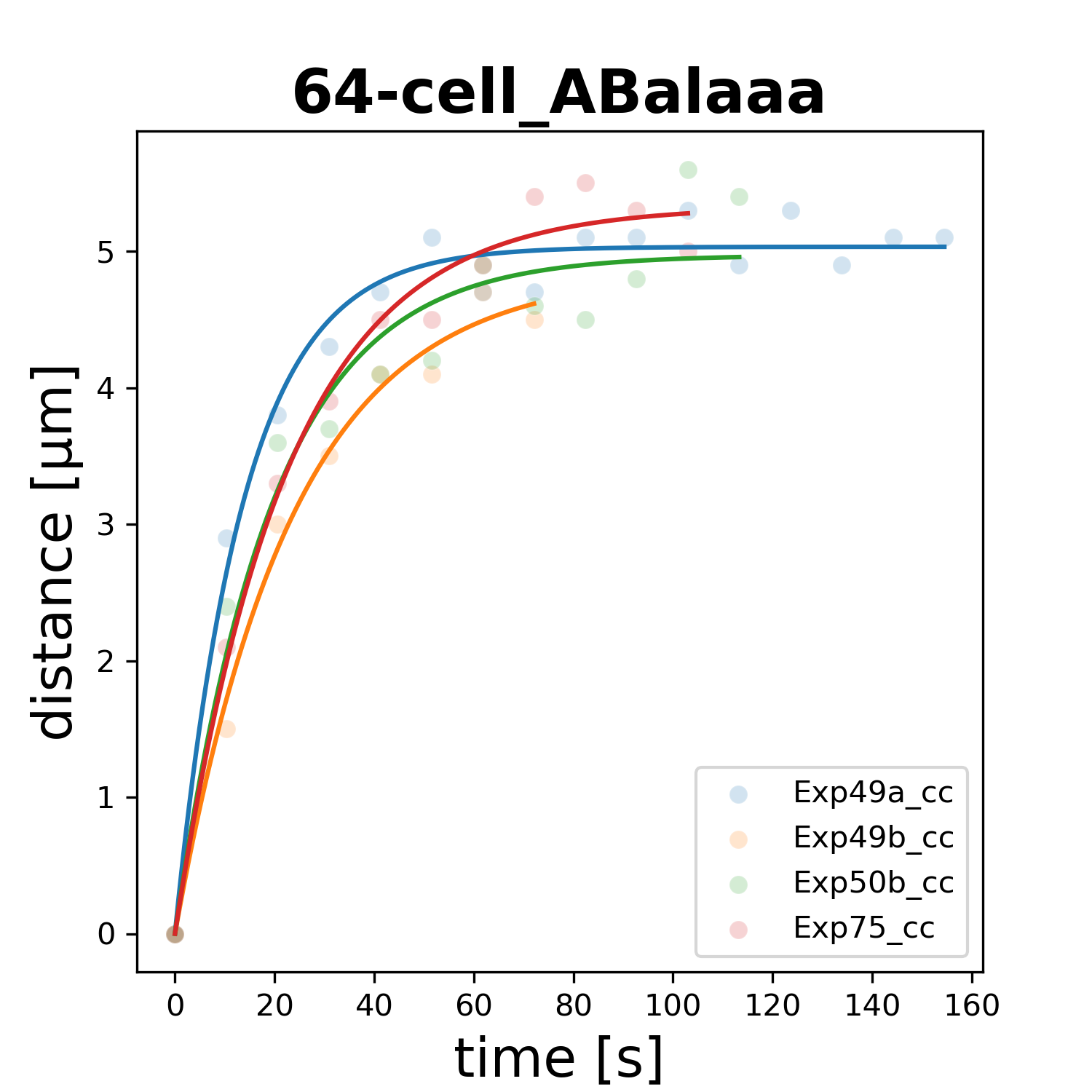

Supplement: Supplement 2 [file media-2.zip › Supplementary Material/ani2(RNAi)_chromosome_to_chromosome_distance/64-cell_ABalaaa.png]

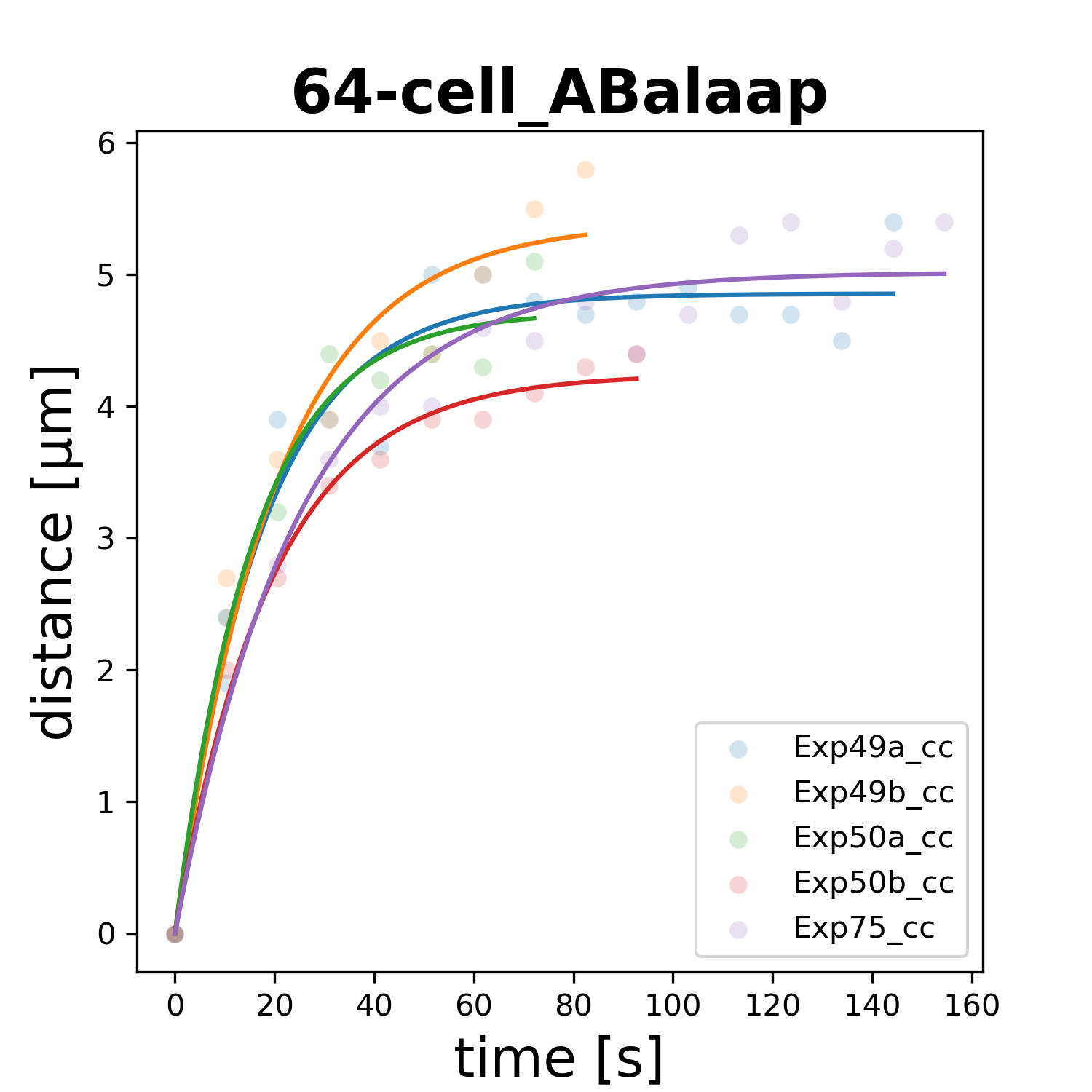

Supplement: Supplement 2 [file media-2.zip › Supplementary Material/ani2(RNAi)_chromosome_to_chromosome_distance/64-cell_ABalaap.png]

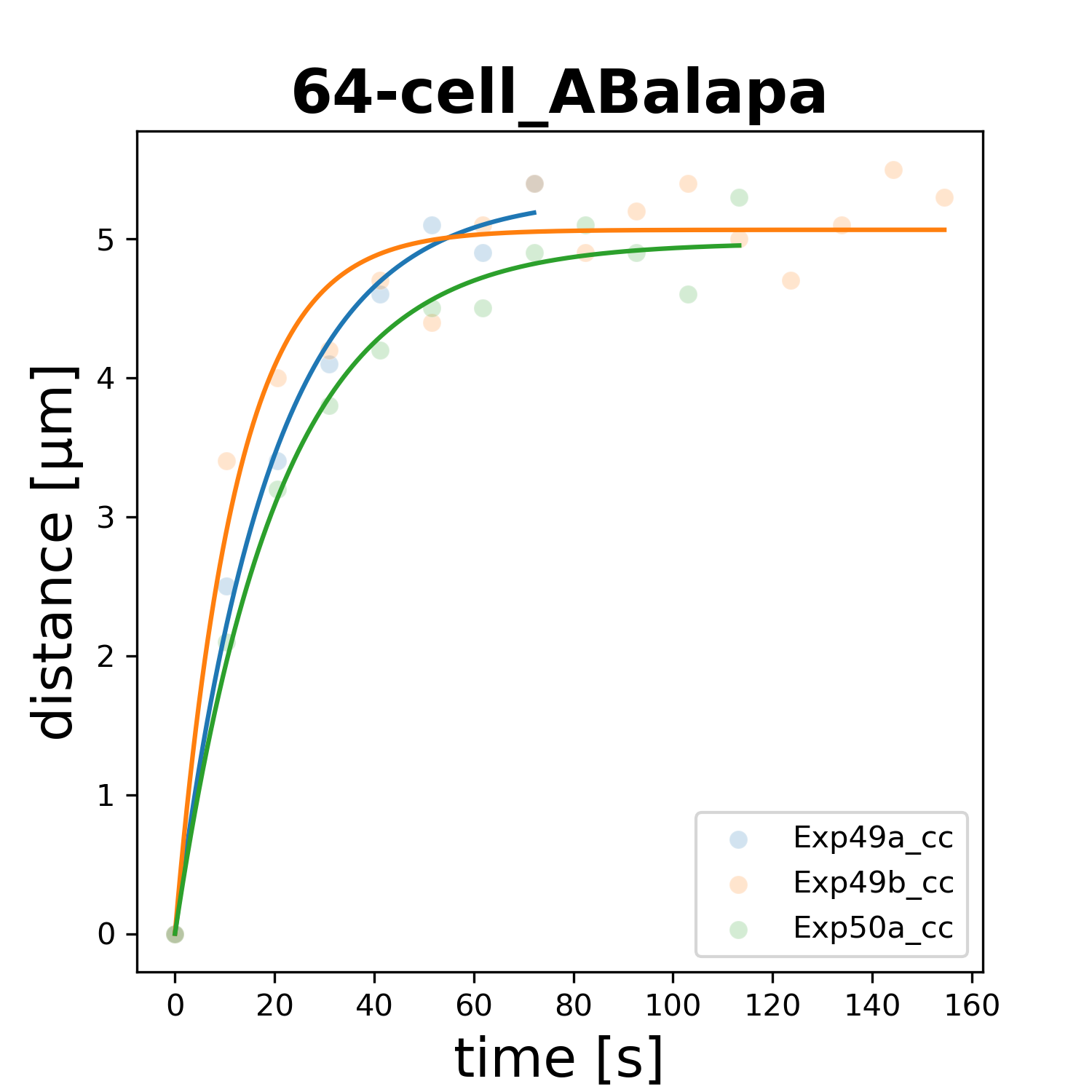

Supplement: Supplement 2 [file media-2.zip › Supplementary Material/ani2(RNAi)_chromosome_to_chromosome_distance/64-cell_ABalapa.png]

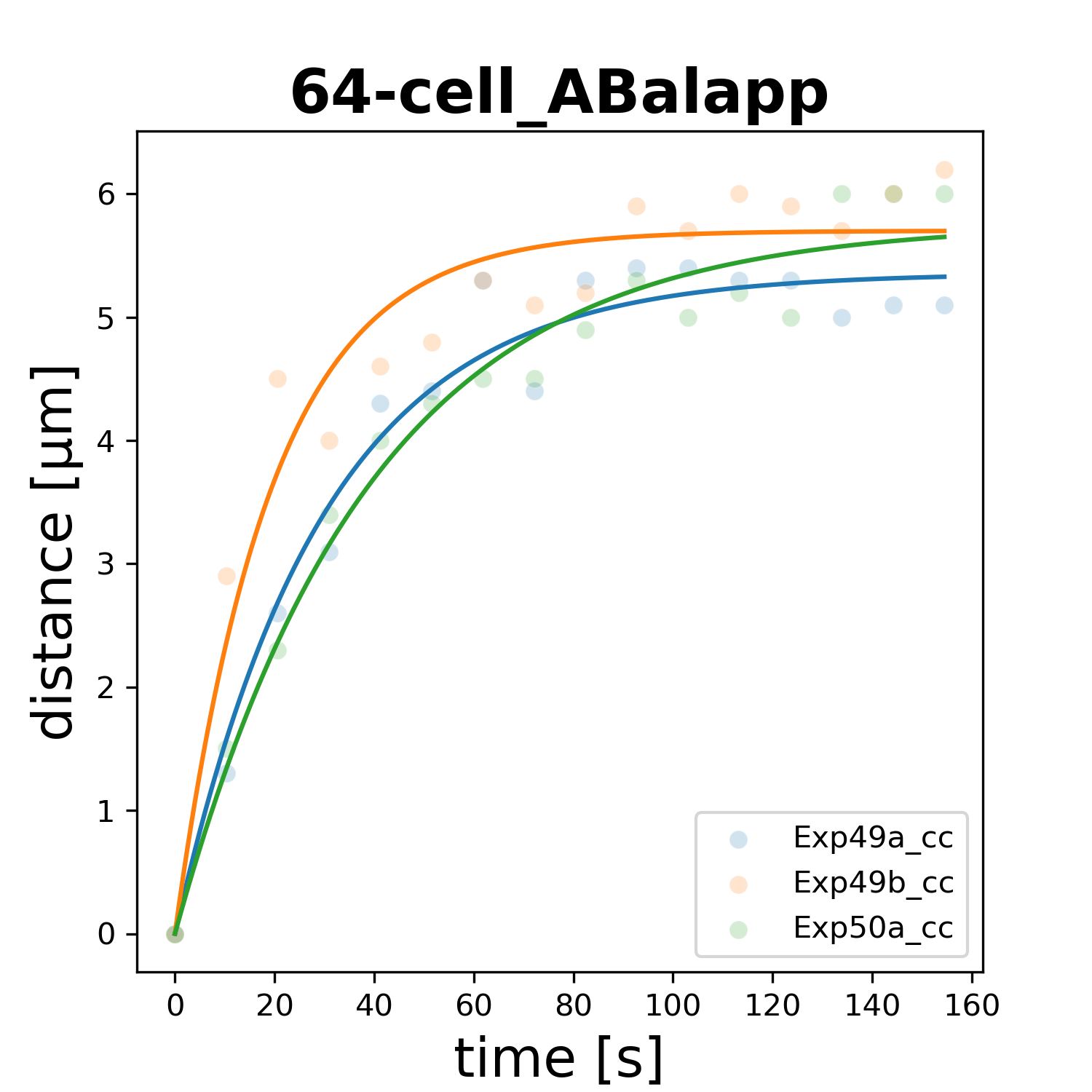

Supplement: Supplement 2 [file media-2.zip › Supplementary Material/ani2(RNAi)_chromosome_to_chromosome_distance/64-cell_ABalapp.png]

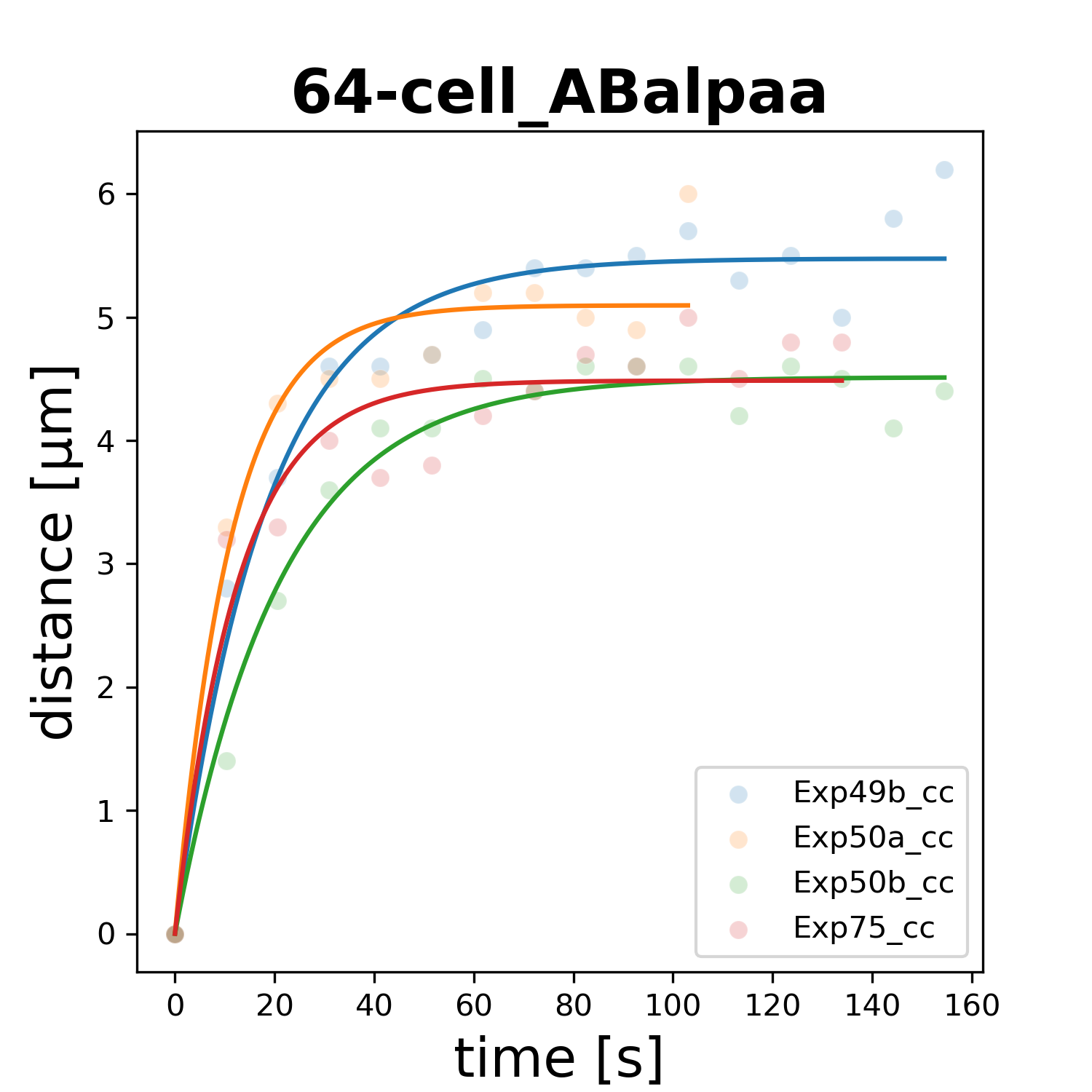

Supplement: Supplement 2 [file media-2.zip › Supplementary Material/ani2(RNAi)_chromosome_to_chromosome_distance/64-cell_ABalpaa.png]

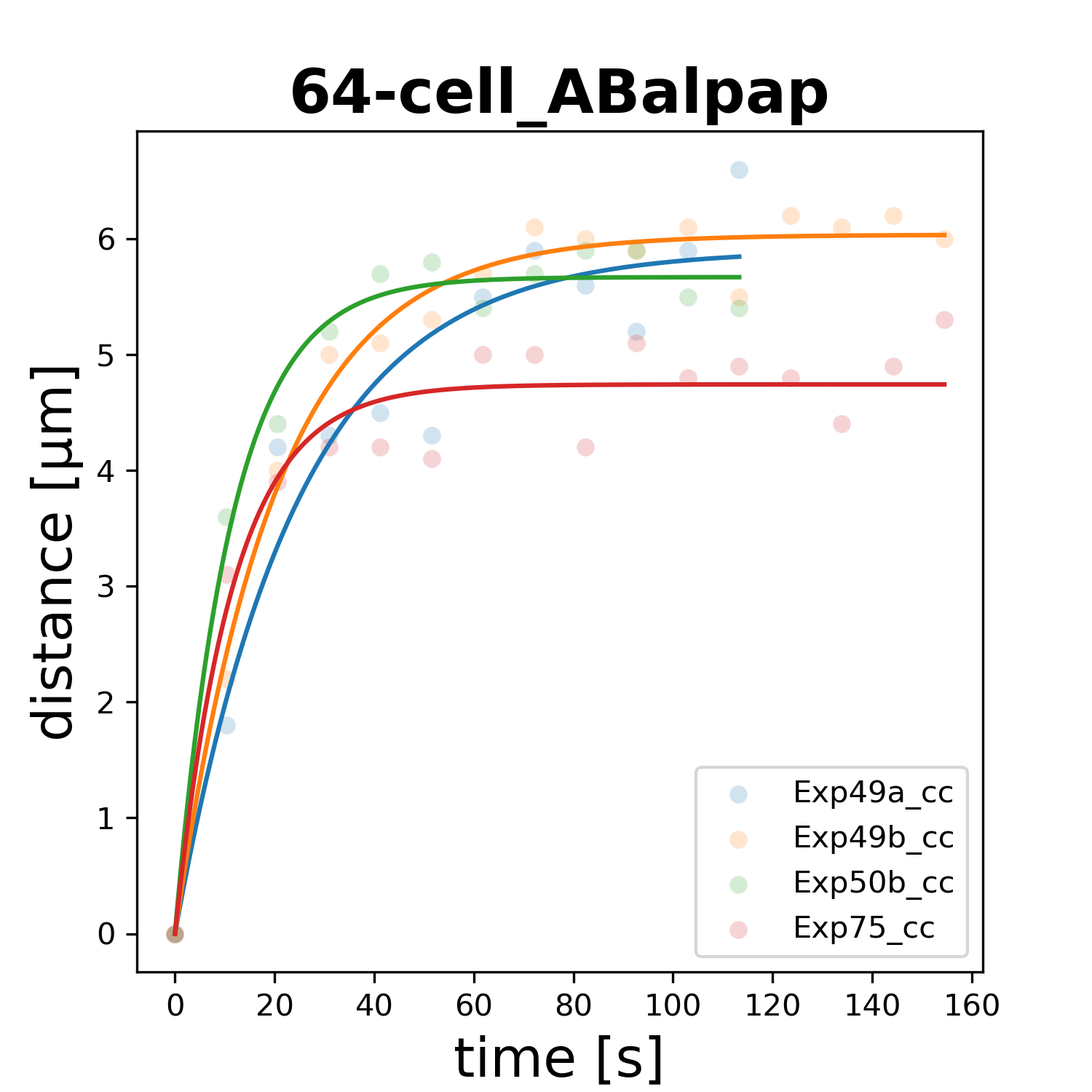

Supplement: Supplement 2 [file media-2.zip › Supplementary Material/ani2(RNAi)_chromosome_to_chromosome_distance/64-cell_ABalpap.png]

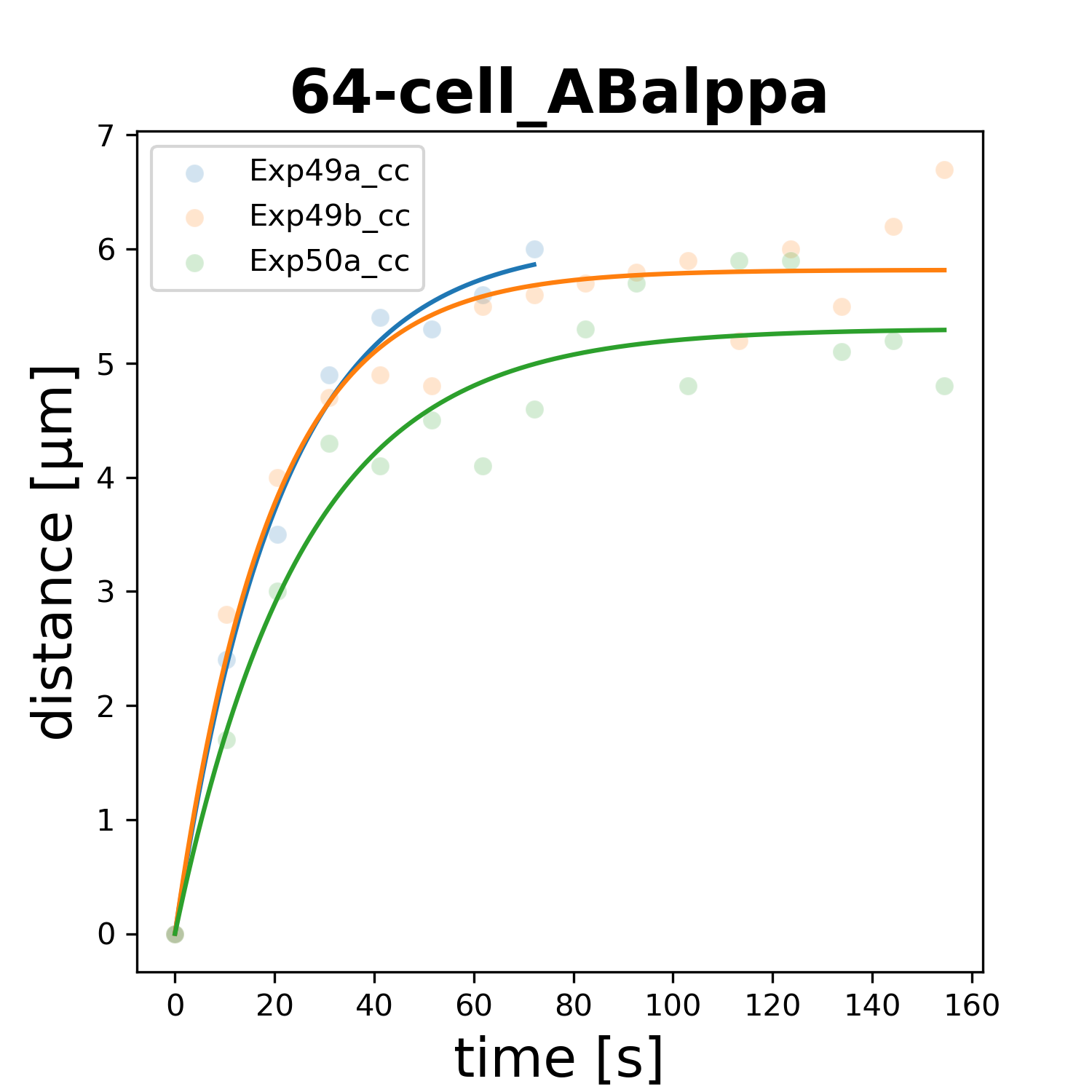

Supplement: Supplement 2 [file media-2.zip › Supplementary Material/ani2(RNAi)_chromosome_to_chromosome_distance/64-cell_ABalppa.png]

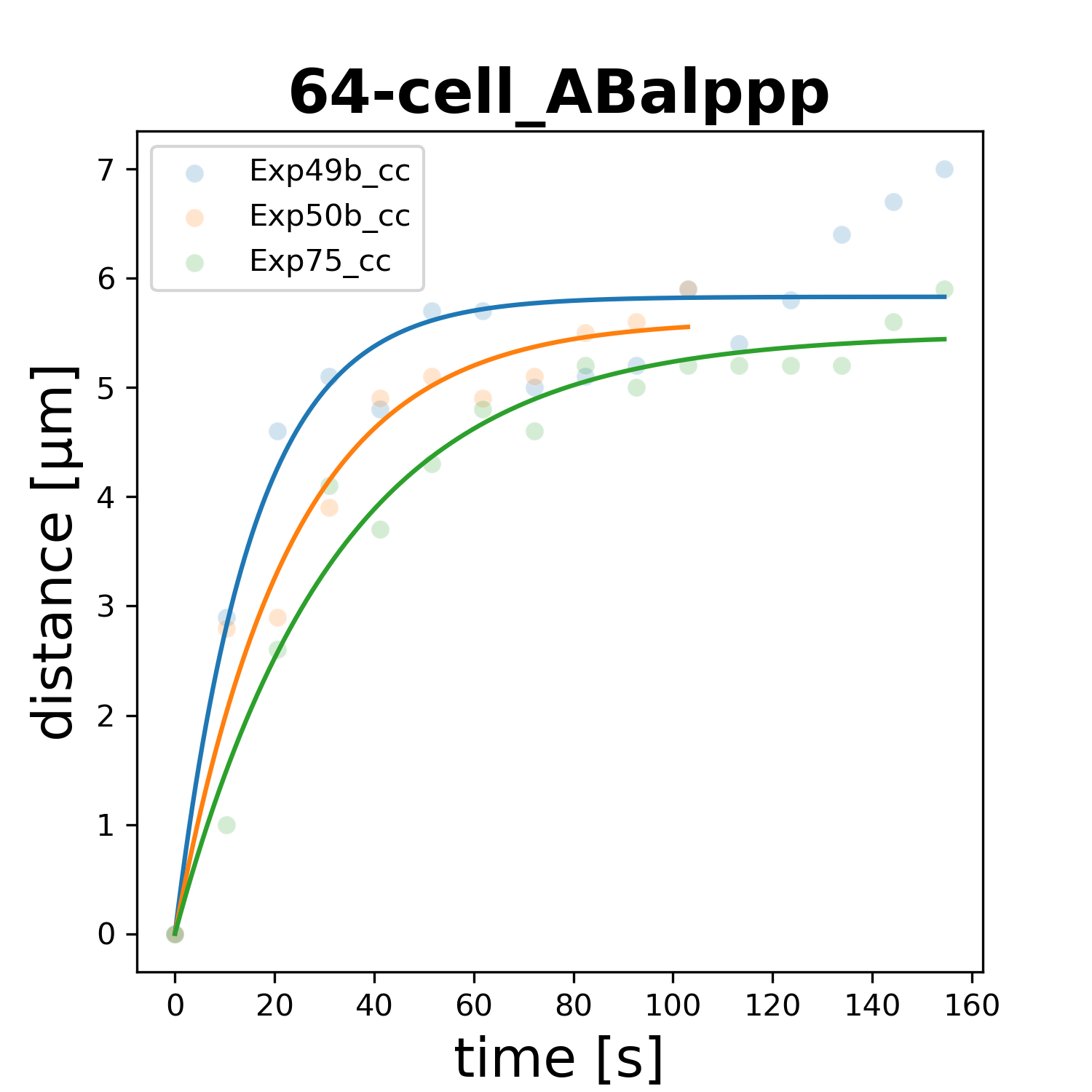

Supplement: Supplement 2 [file media-2.zip › Supplementary Material/ani2(RNAi)_chromosome_to_chromosome_distance/64-cell_ABalppp.png]

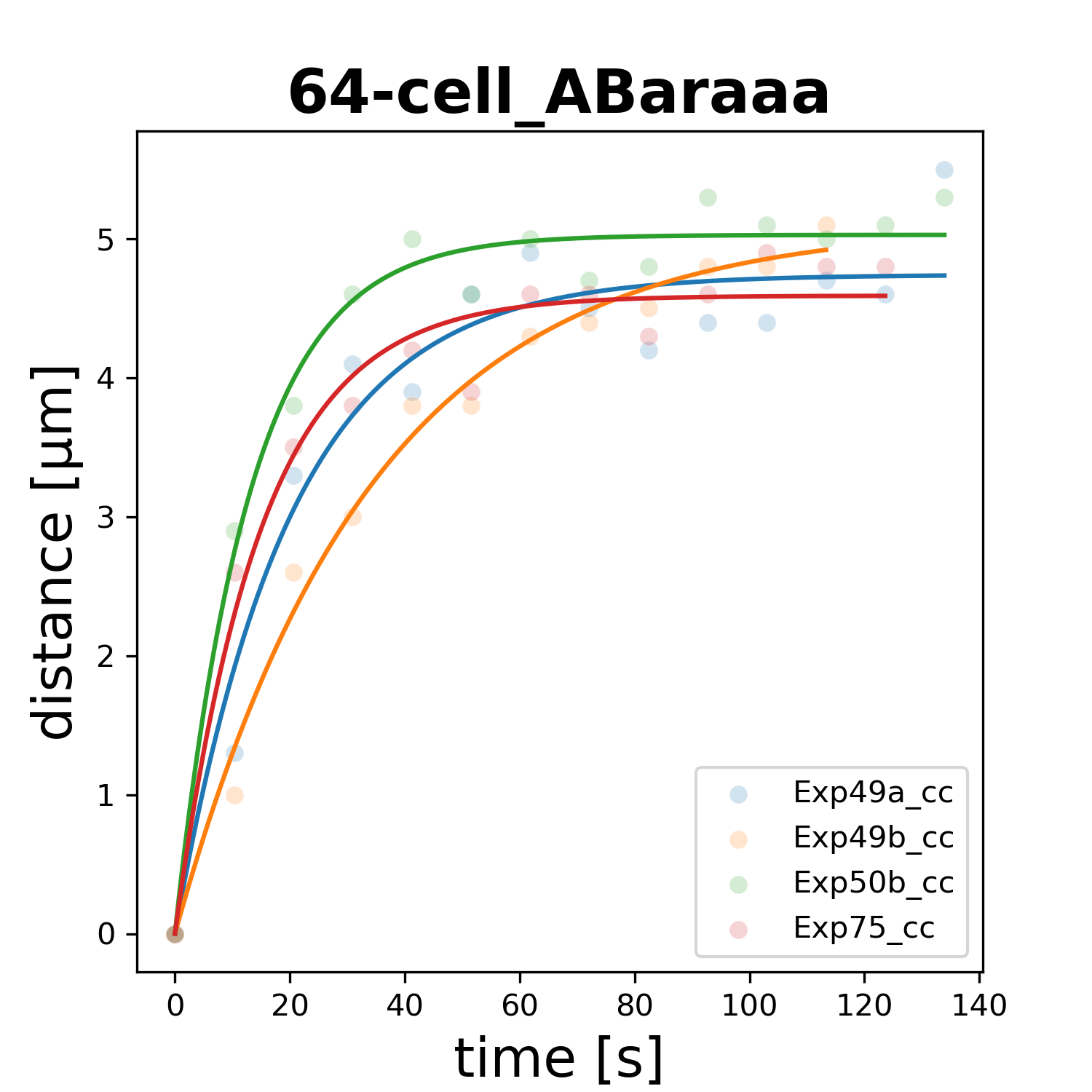

Supplement: Supplement 2 [file media-2.zip › Supplementary Material/ani2(RNAi)_chromosome_to_chromosome_distance/64-cell_ABaraaa.png]

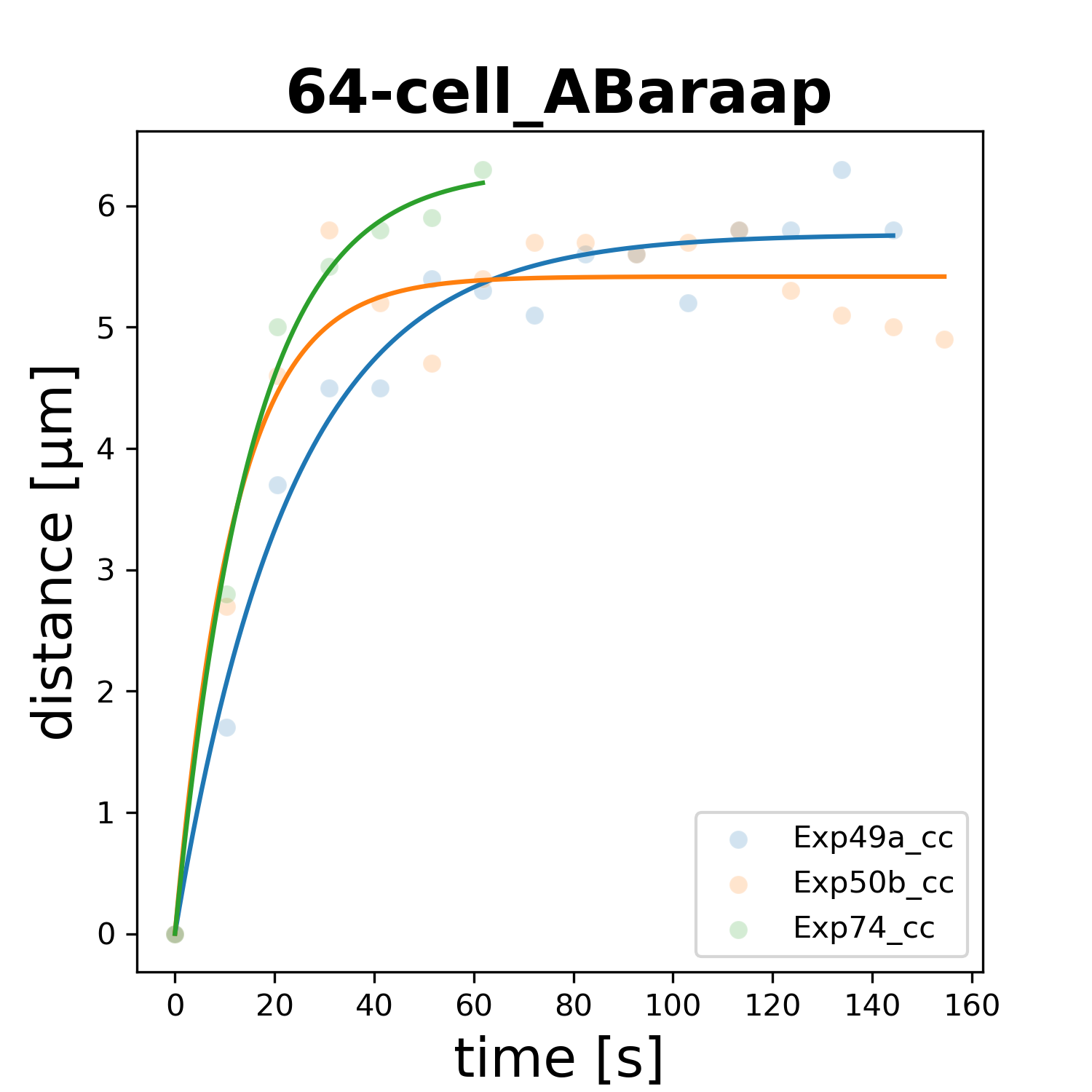

Supplement: Supplement 2 [file media-2.zip › Supplementary Material/ani2(RNAi)_chromosome_to_chromosome_distance/64-cell_ABaraap.png]

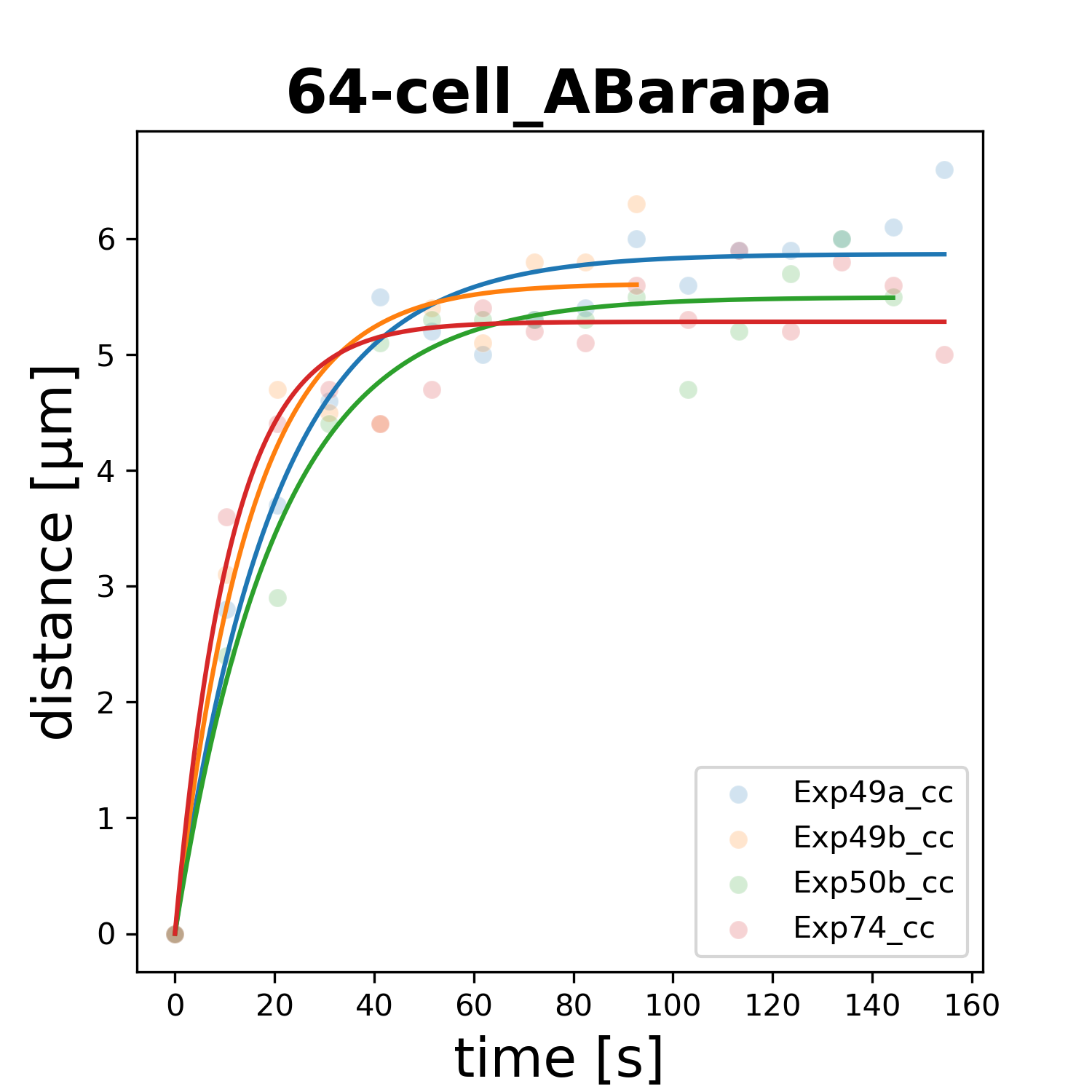

Supplement: Supplement 2 [file media-2.zip › Supplementary Material/ani2(RNAi)_chromosome_to_chromosome_distance/64-cell_ABarapa.png]

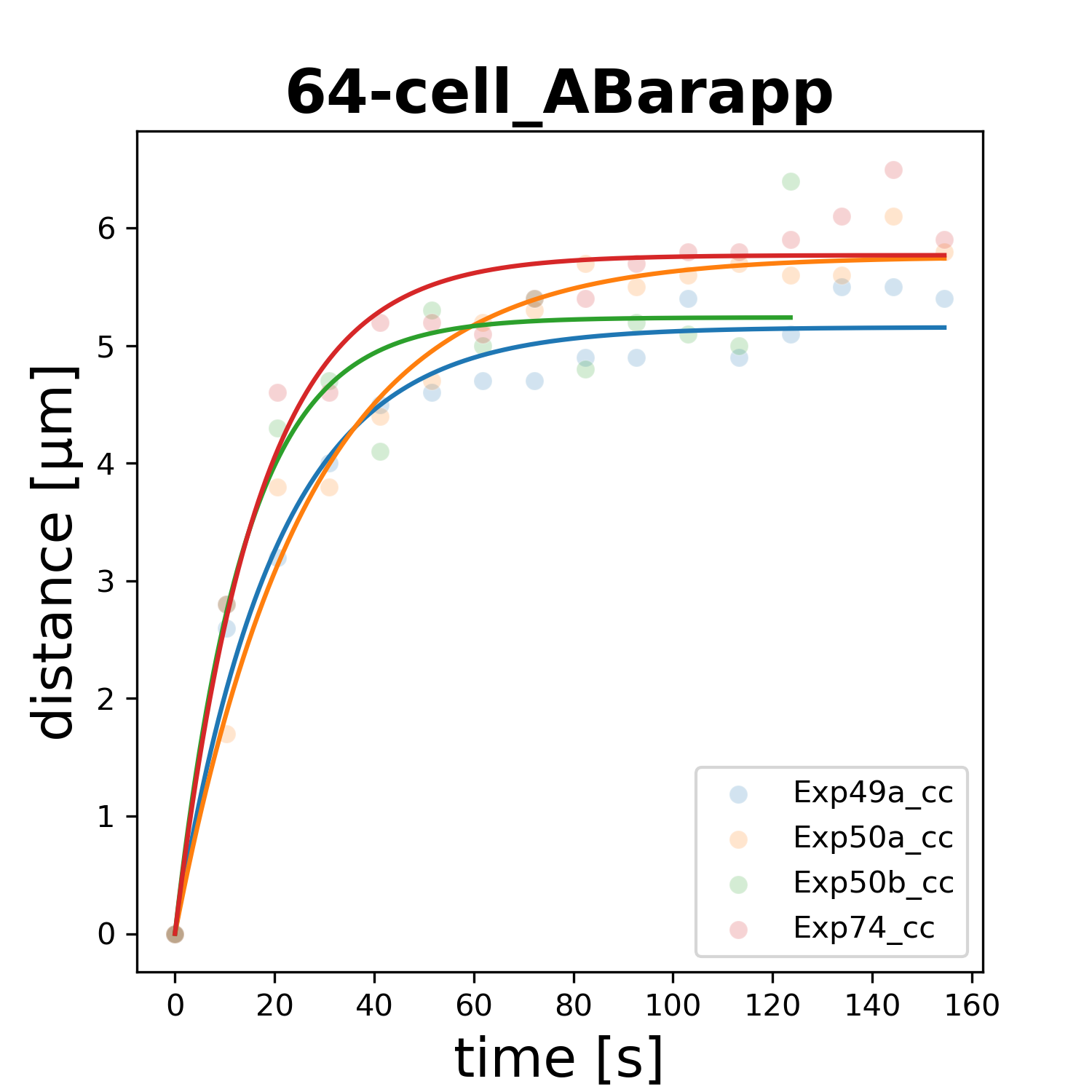

Supplement: Supplement 2 [file media-2.zip › Supplementary Material/ani2(RNAi)_chromosome_to_chromosome_distance/64-cell_ABarapp.png]

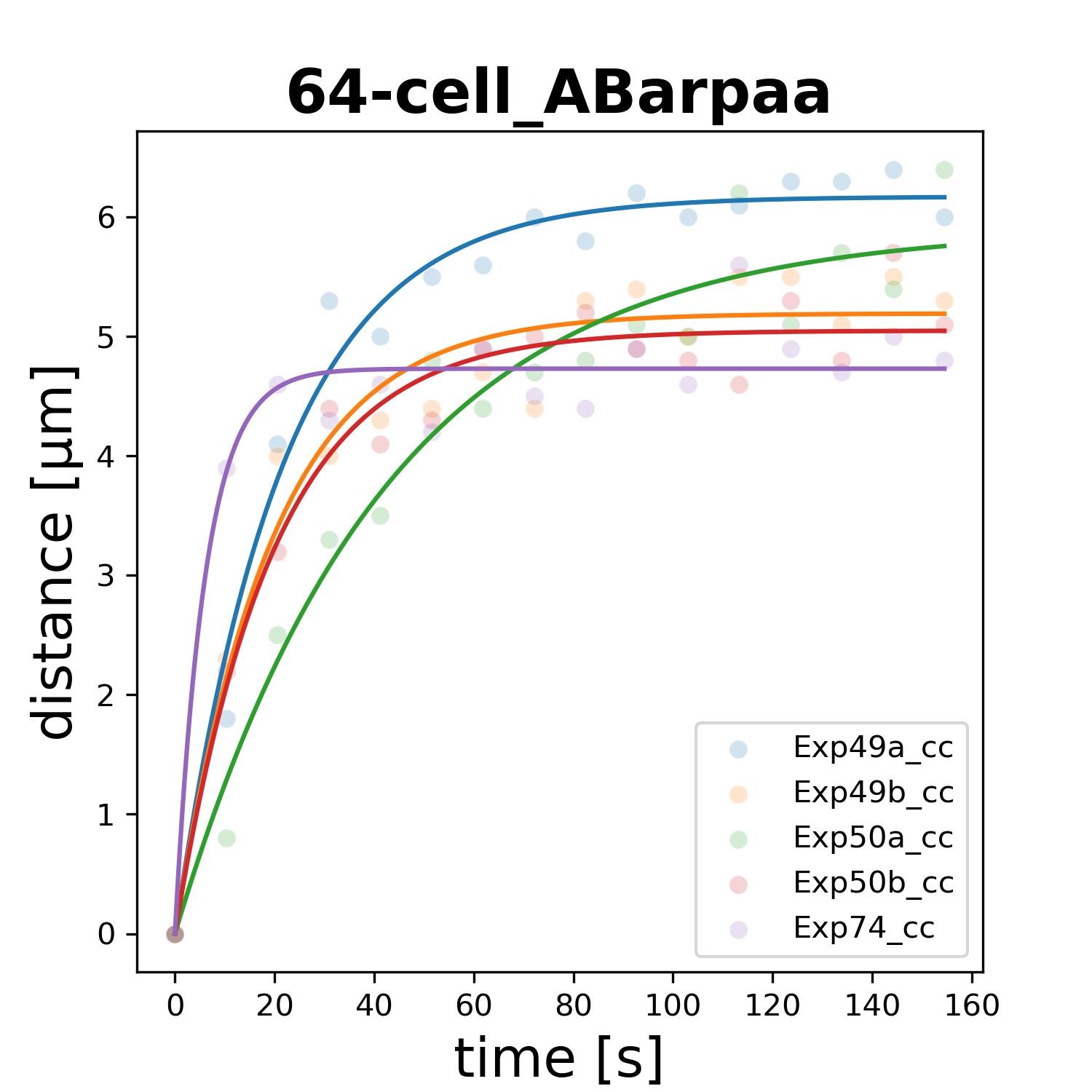

Supplement: Supplement 2 [file media-2.zip › Supplementary Material/ani2(RNAi)_chromosome_to_chromosome_distance/64-cell_ABarpaa.png]

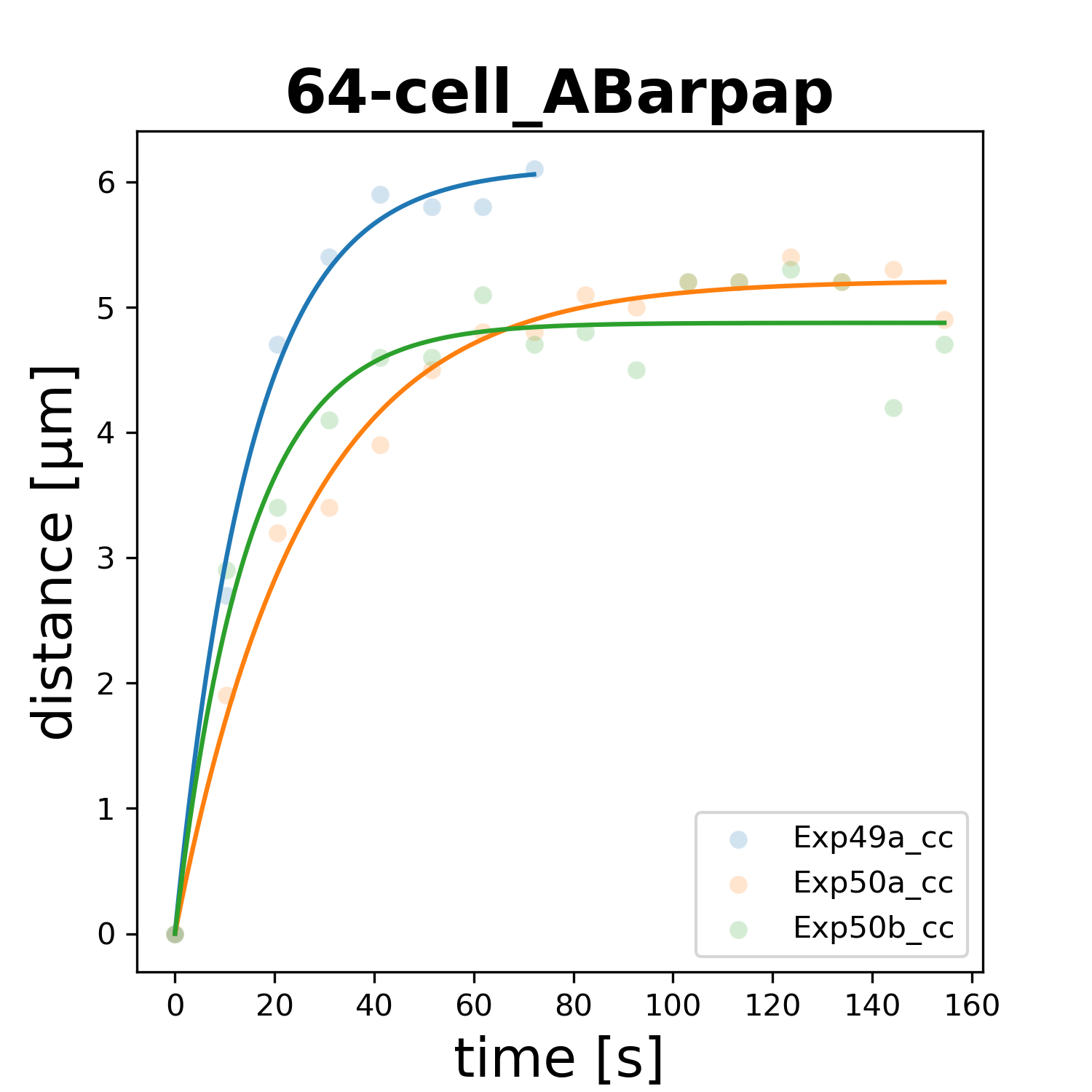

Supplement: Supplement 2 [file media-2.zip › Supplementary Material/ani2(RNAi)_chromosome_to_chromosome_distance/64-cell_ABarpap.png]

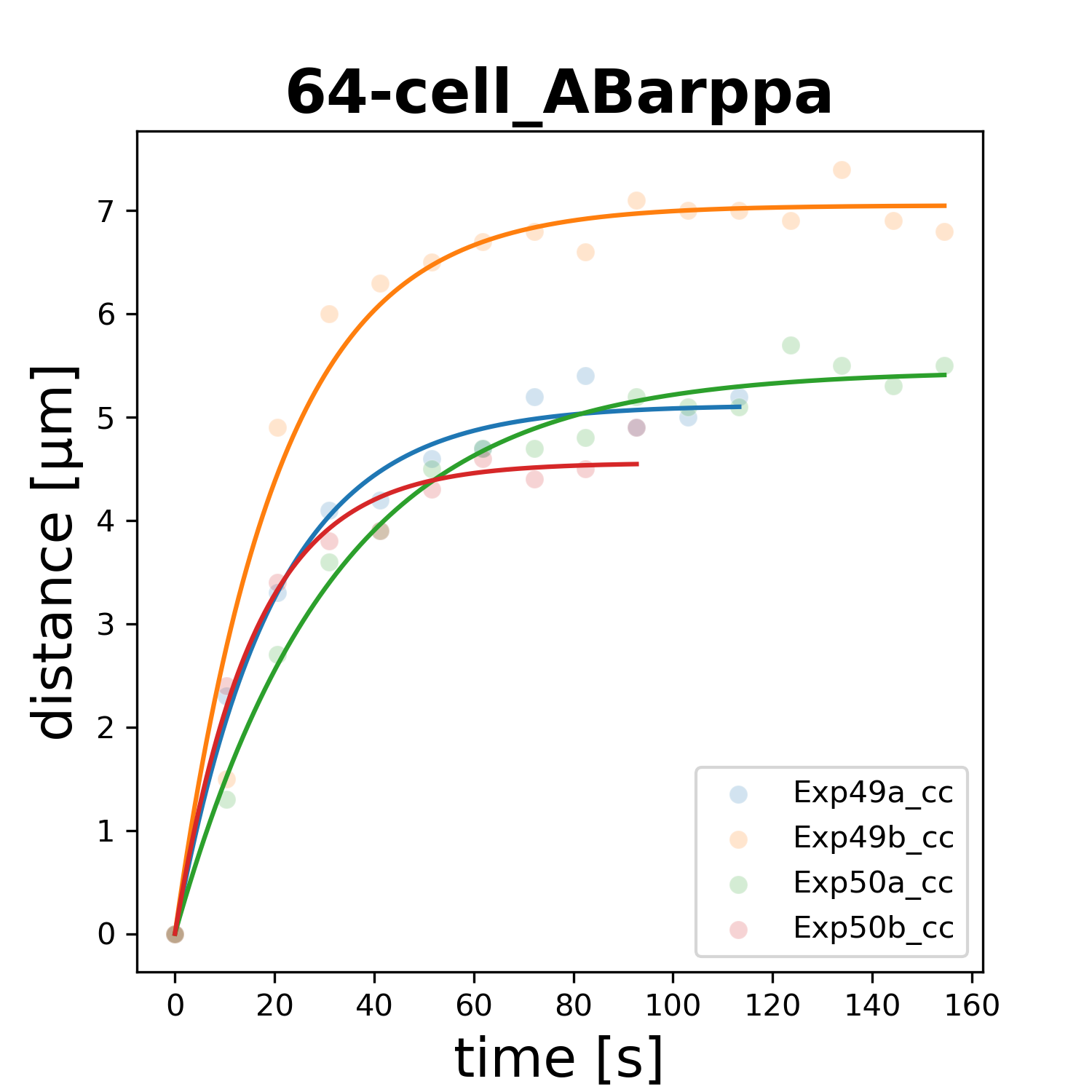

Supplement: Supplement 2 [file media-2.zip › Supplementary Material/ani2(RNAi)_chromosome_to_chromosome_distance/64-cell_ABarppa.png]

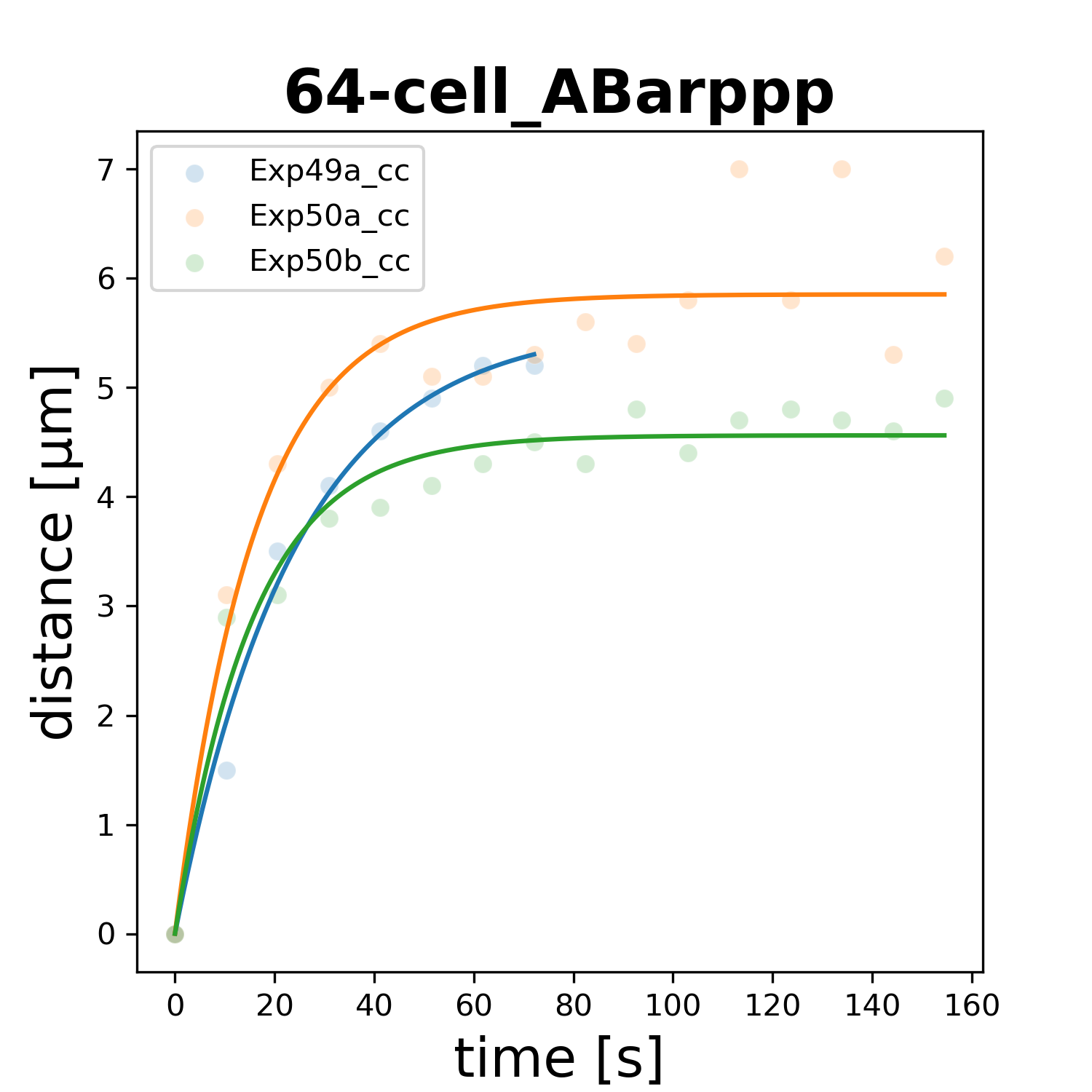

Supplement: Supplement 2 [file media-2.zip › Supplementary Material/ani2(RNAi)_chromosome_to_chromosome_distance/64-cell_ABarppp.png]

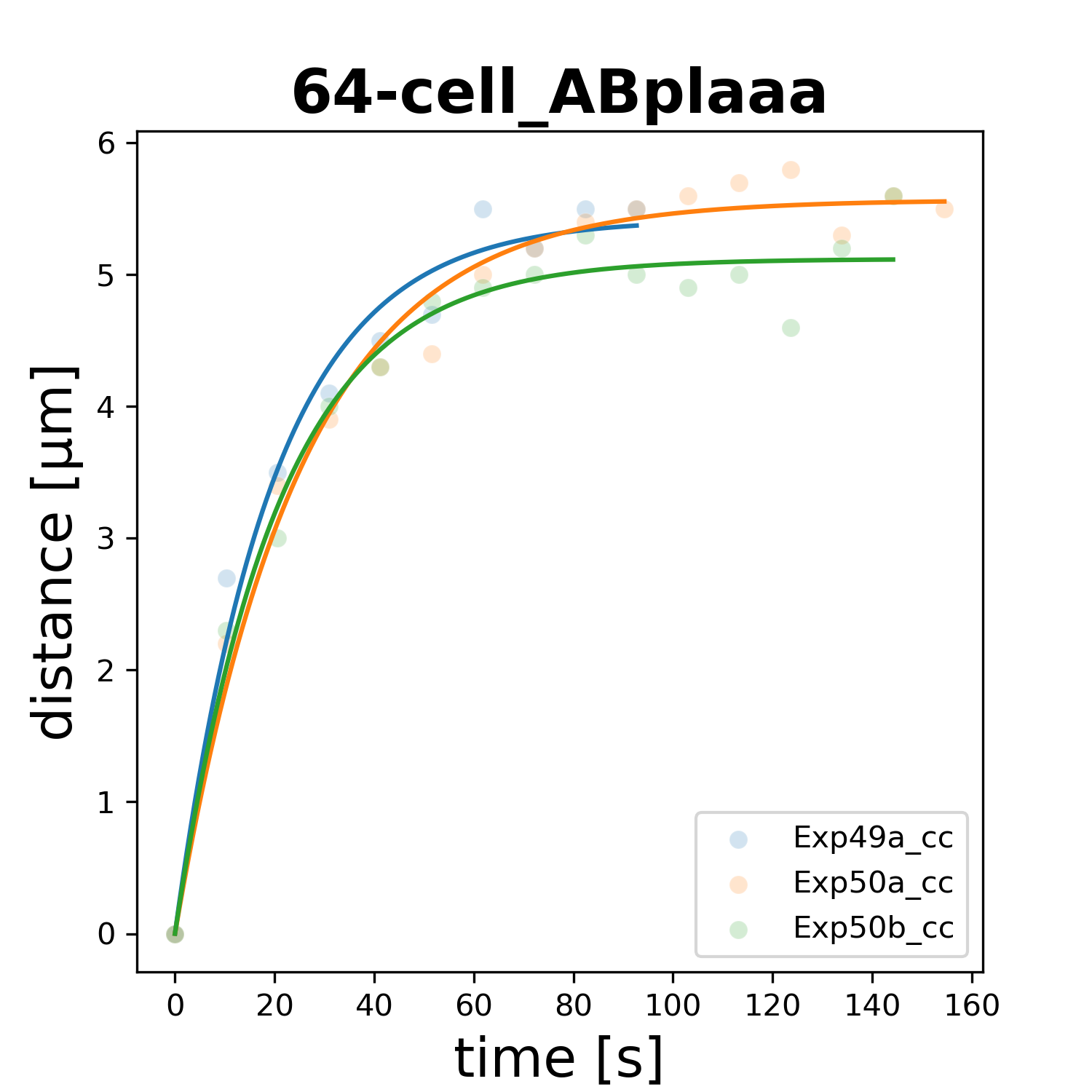

Supplement: Supplement 2 [file media-2.zip › Supplementary Material/ani2(RNAi)_chromosome_to_chromosome_distance/64-cell_ABplaaa.png]

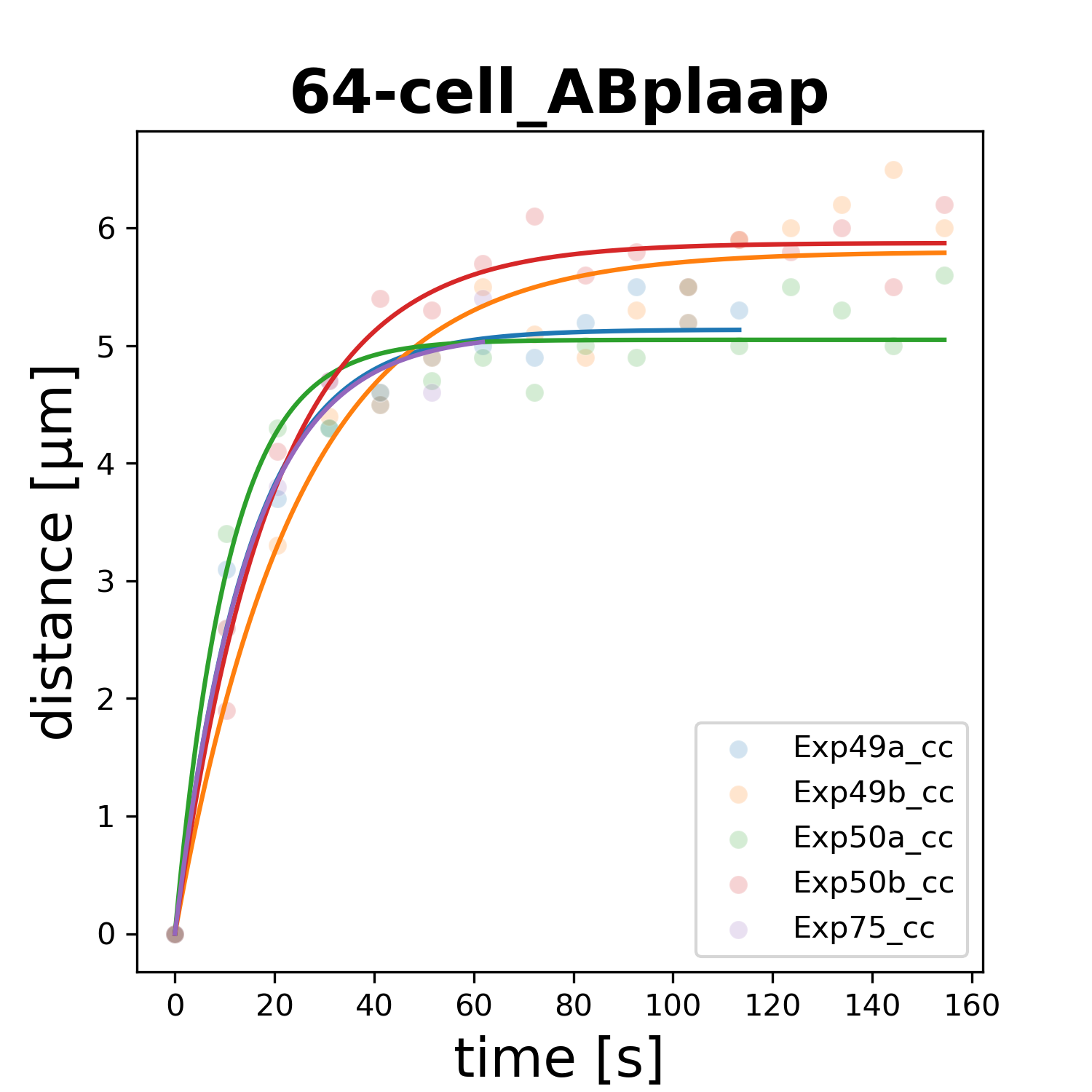

Supplement: Supplement 2 [file media-2.zip › Supplementary Material/ani2(RNAi)_chromosome_to_chromosome_distance/64-cell_ABplaap.png]

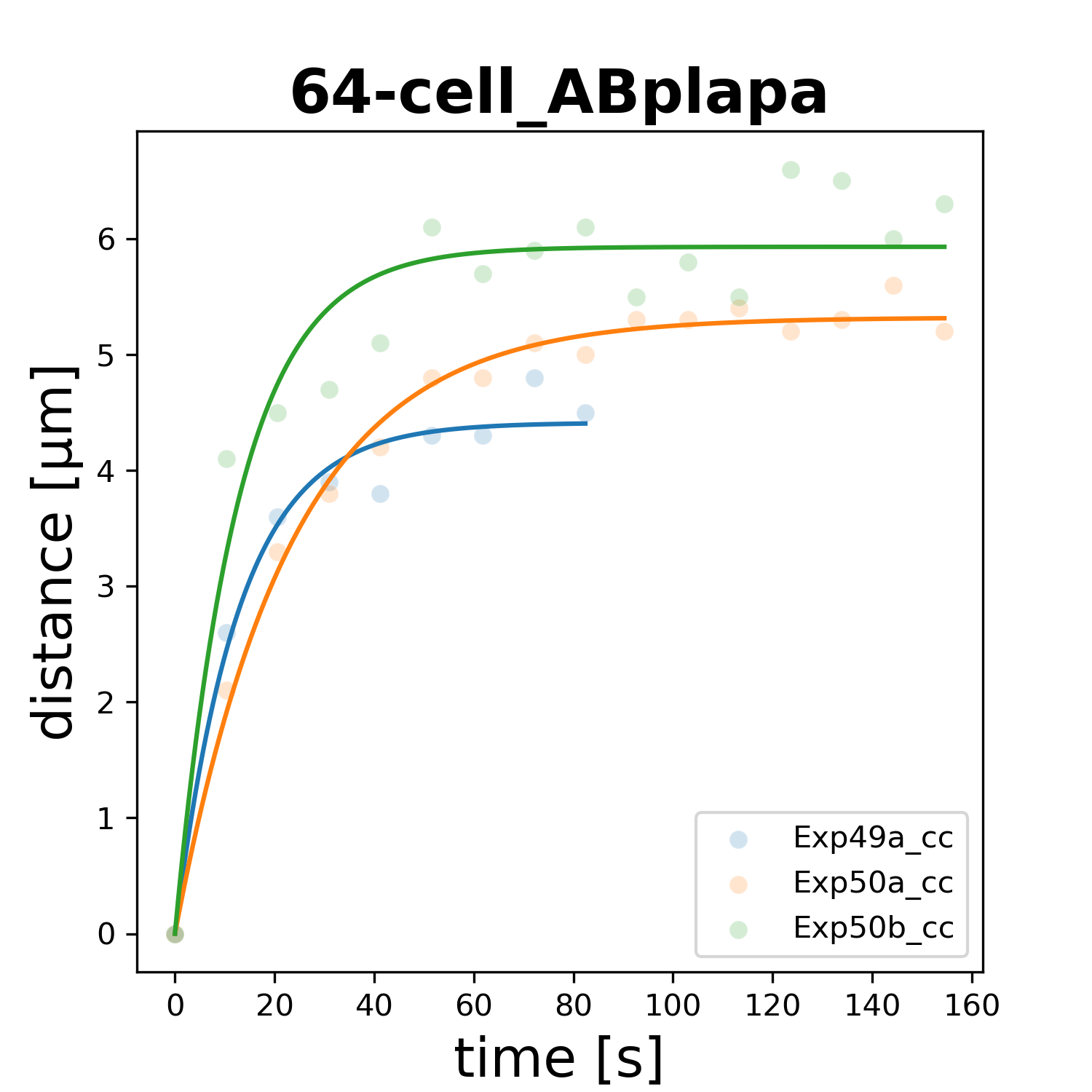

Supplement: Supplement 2 [file media-2.zip › Supplementary Material/ani2(RNAi)_chromosome_to_chromosome_distance/64-cell_ABplapa.png]

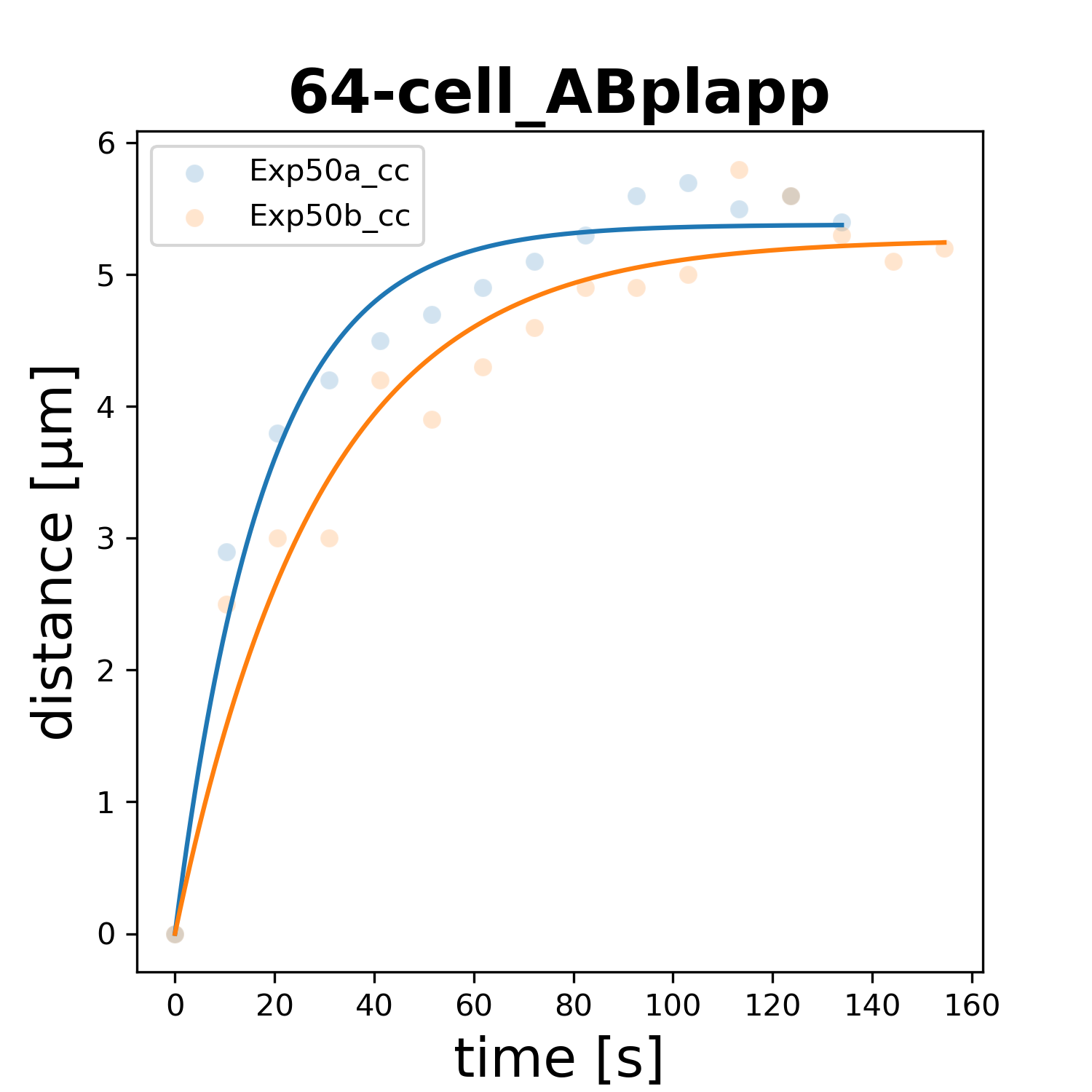

Supplement: Supplement 2 [file media-2.zip › Supplementary Material/ani2(RNAi)_chromosome_to_chromosome_distance/64-cell_ABplapp.png]

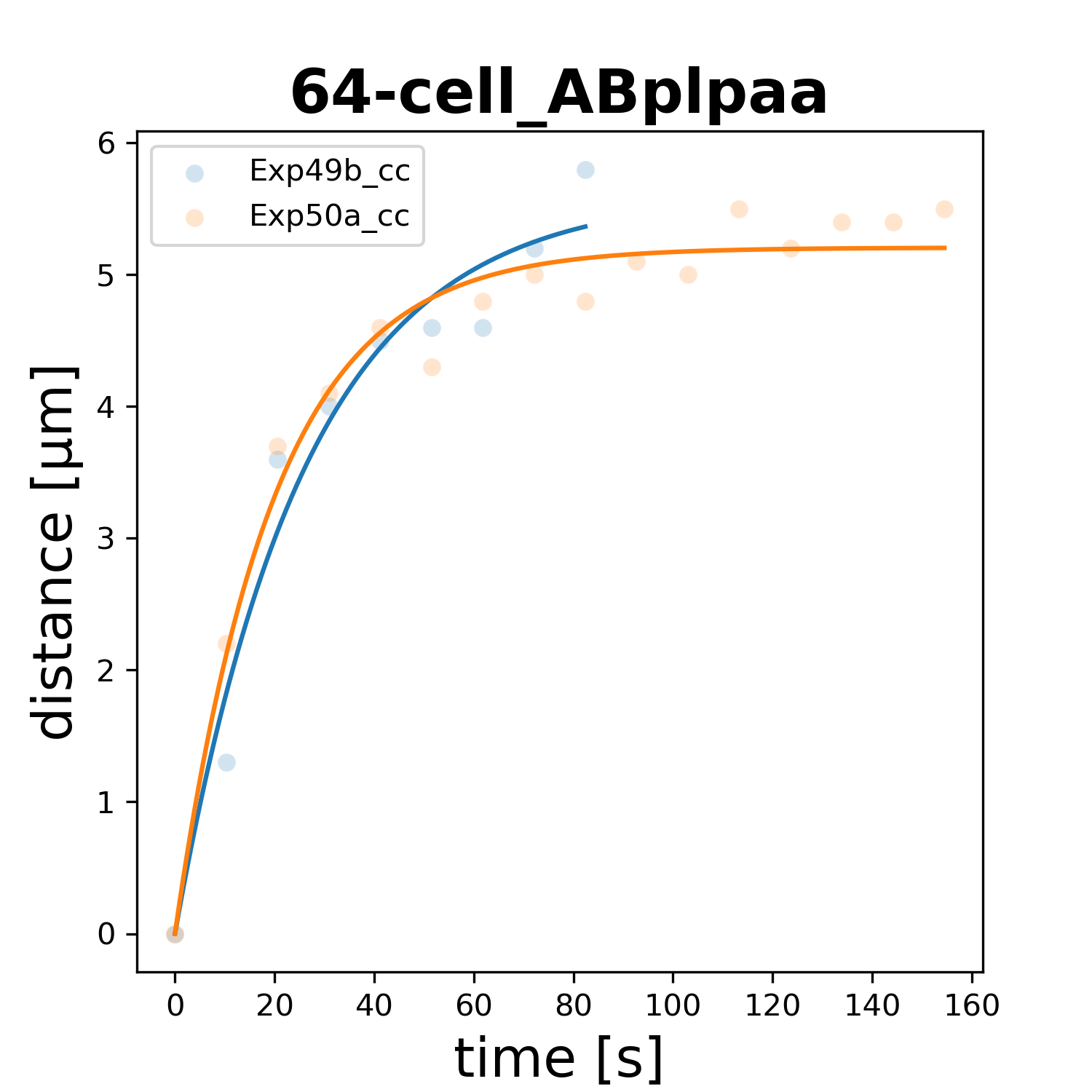

Supplement: Supplement 2 [file media-2.zip › Supplementary Material/ani2(RNAi)_chromosome_to_chromosome_distance/64-cell_ABplpaa.png]

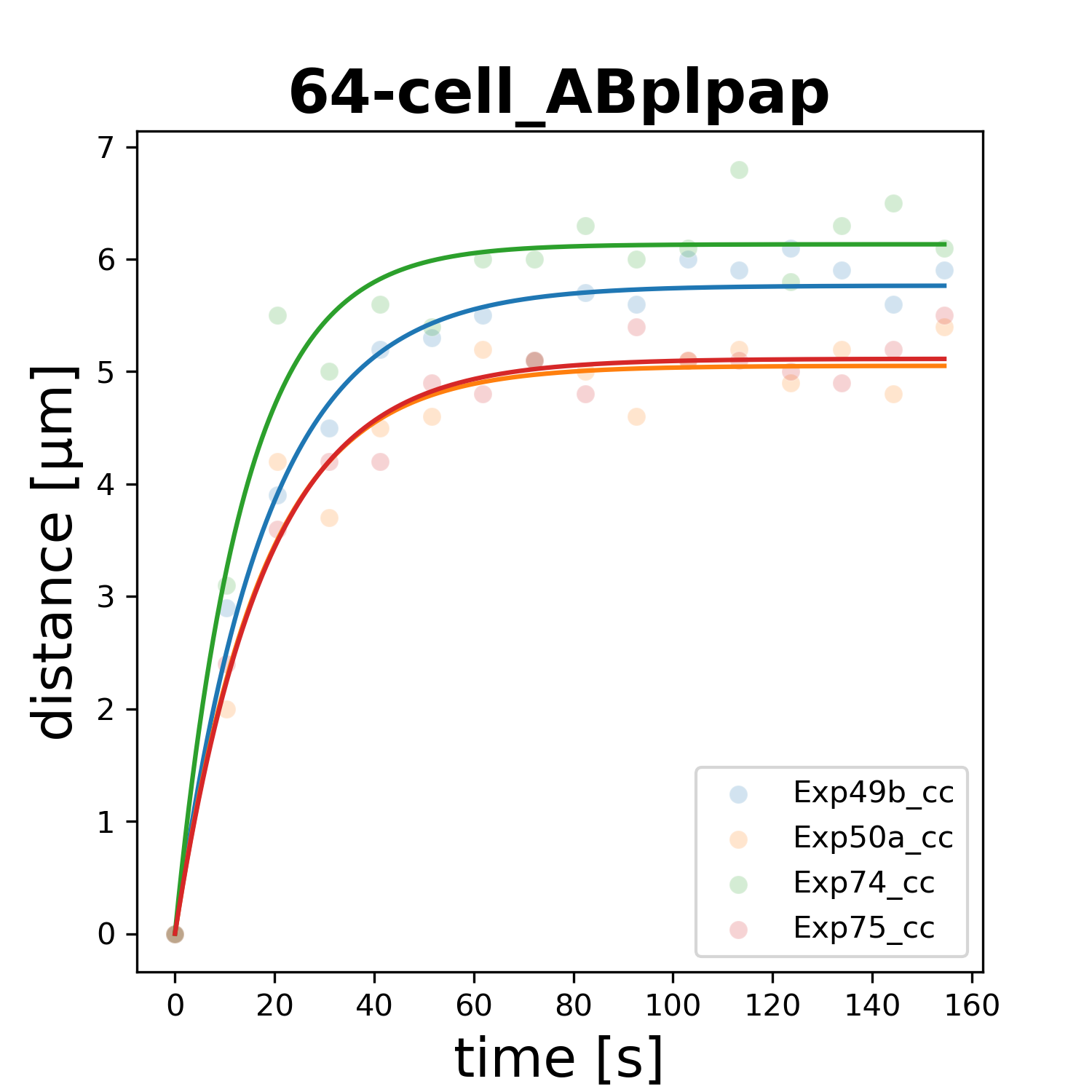

Supplement: Supplement 2 [file media-2.zip › Supplementary Material/ani2(RNAi)_chromosome_to_chromosome_distance/64-cell_ABplpap.png]

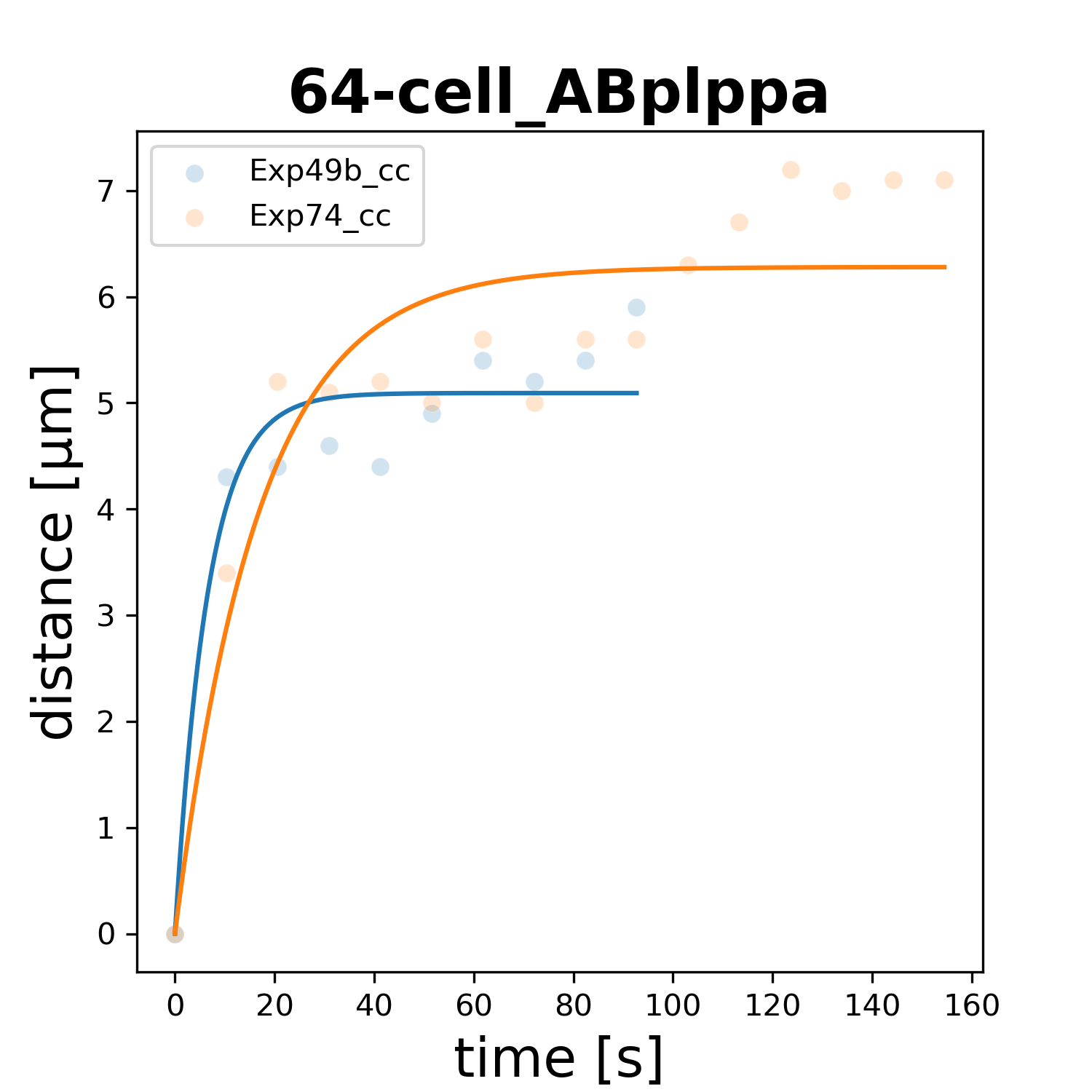

Supplement: Supplement 2 [file media-2.zip › Supplementary Material/ani2(RNAi)_chromosome_to_chromosome_distance/64-cell_ABplppa.png]

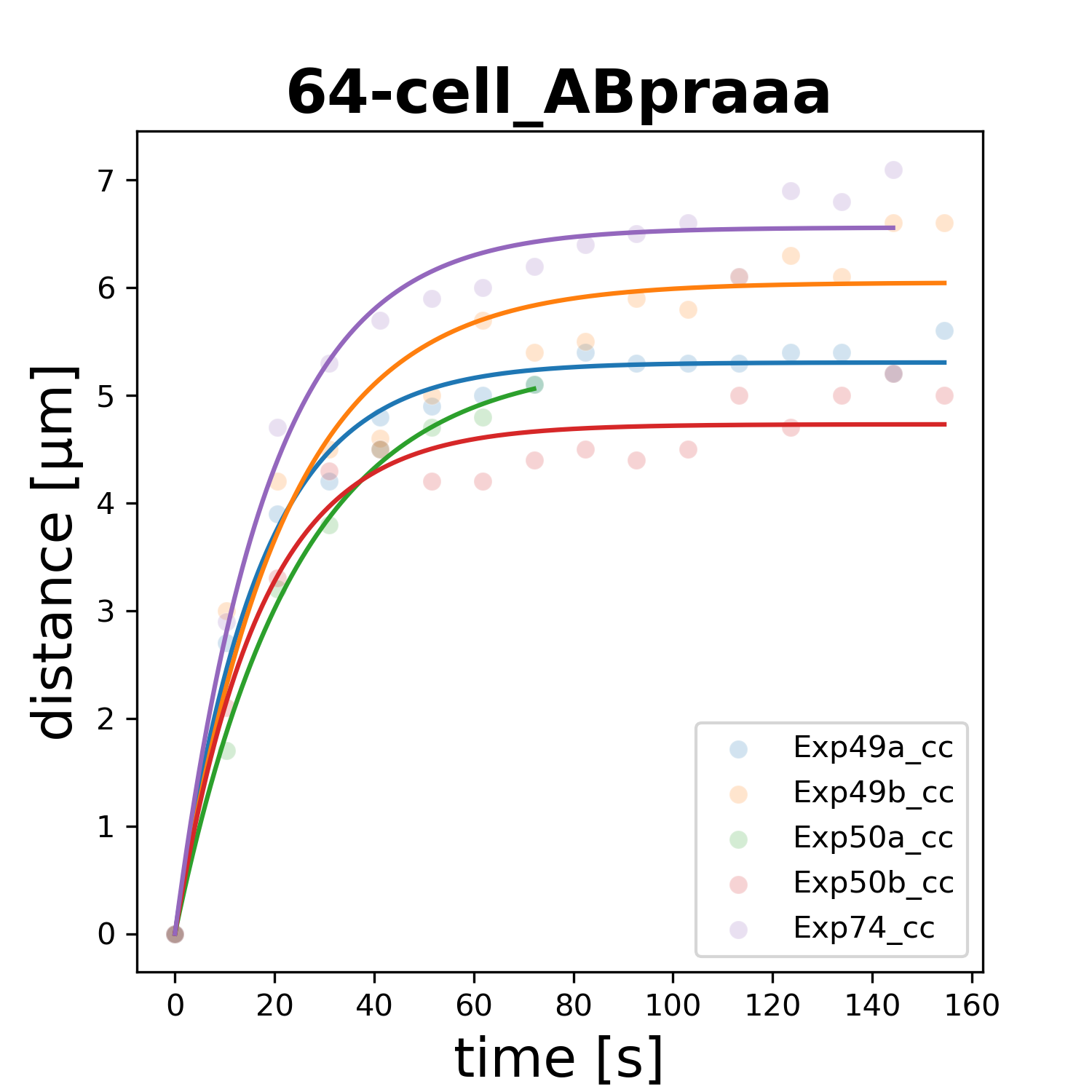

Supplement: Supplement 2 [file media-2.zip › Supplementary Material/ani2(RNAi)_chromosome_to_chromosome_distance/64-cell_ABpraaa.png]

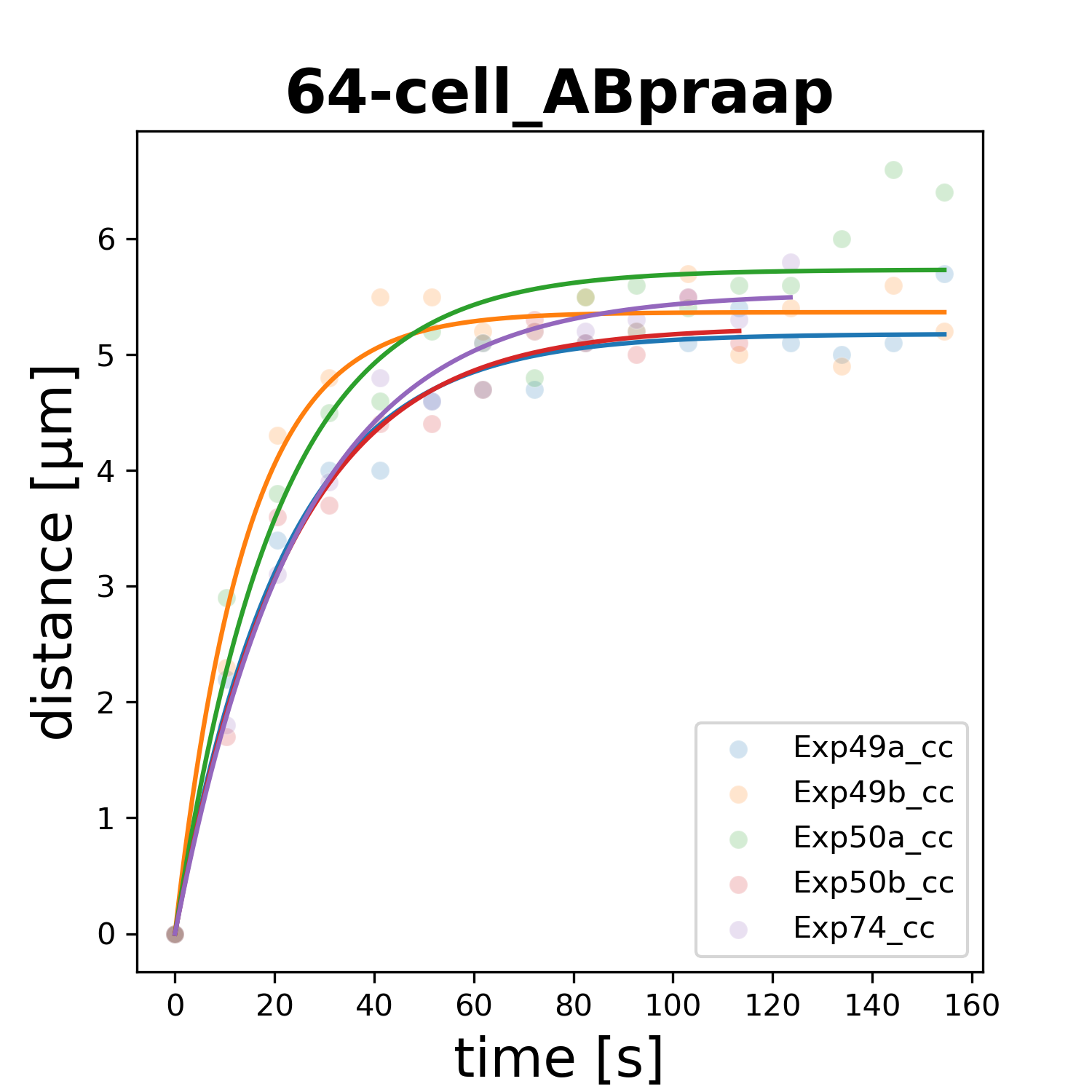

Supplement: Supplement 2 [file media-2.zip › Supplementary Material/ani2(RNAi)_chromosome_to_chromosome_distance/64-cell_ABpraap.png]

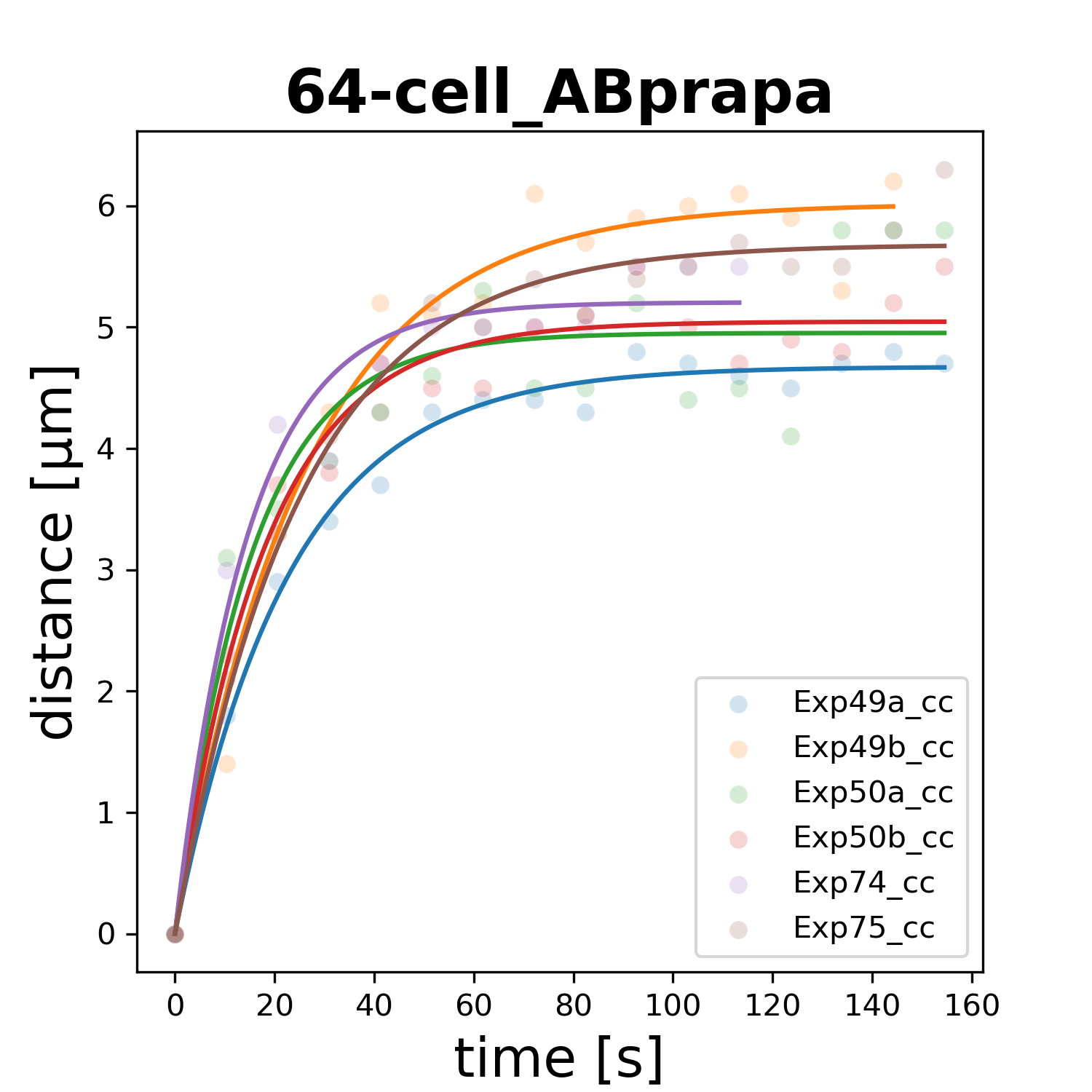

Supplement: Supplement 2 [file media-2.zip › Supplementary Material/ani2(RNAi)_chromosome_to_chromosome_distance/64-cell_ABprapa.png]

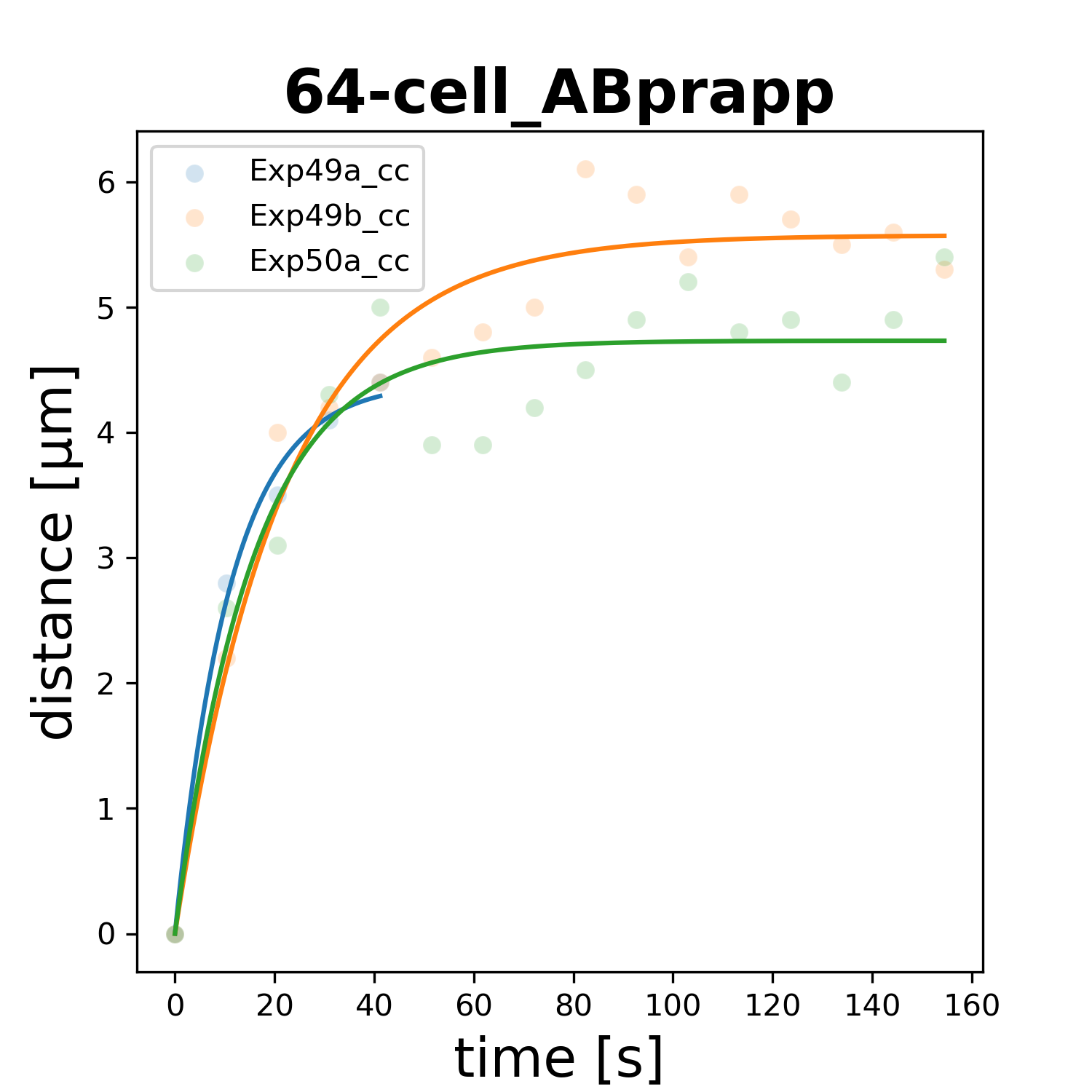

Supplement: Supplement 2 [file media-2.zip › Supplementary Material/ani2(RNAi)_chromosome_to_chromosome_distance/64-cell_ABprapp.png]

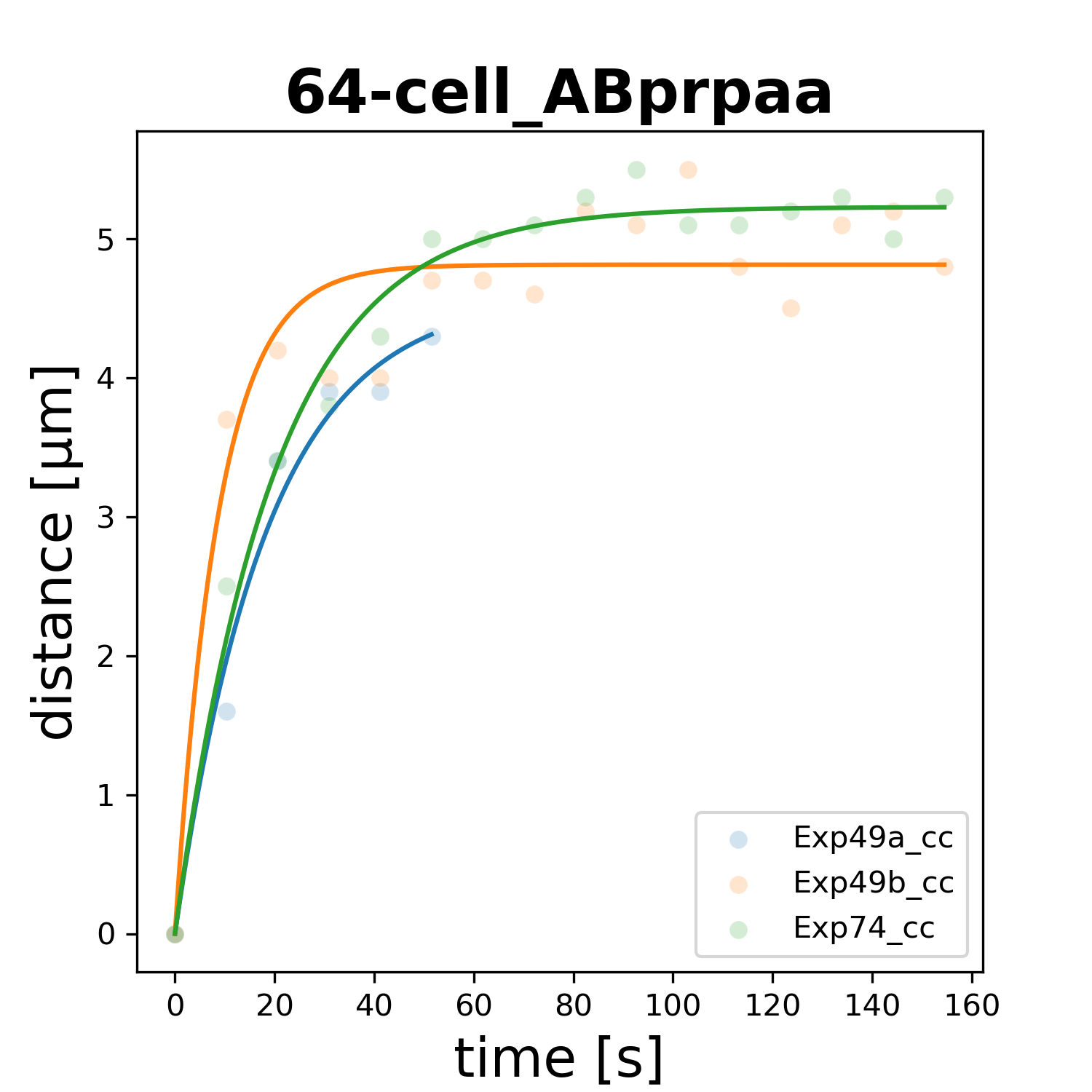

Supplement: Supplement 2 [file media-2.zip › Supplementary Material/ani2(RNAi)_chromosome_to_chromosome_distance/64-cell_ABprpaa.png]

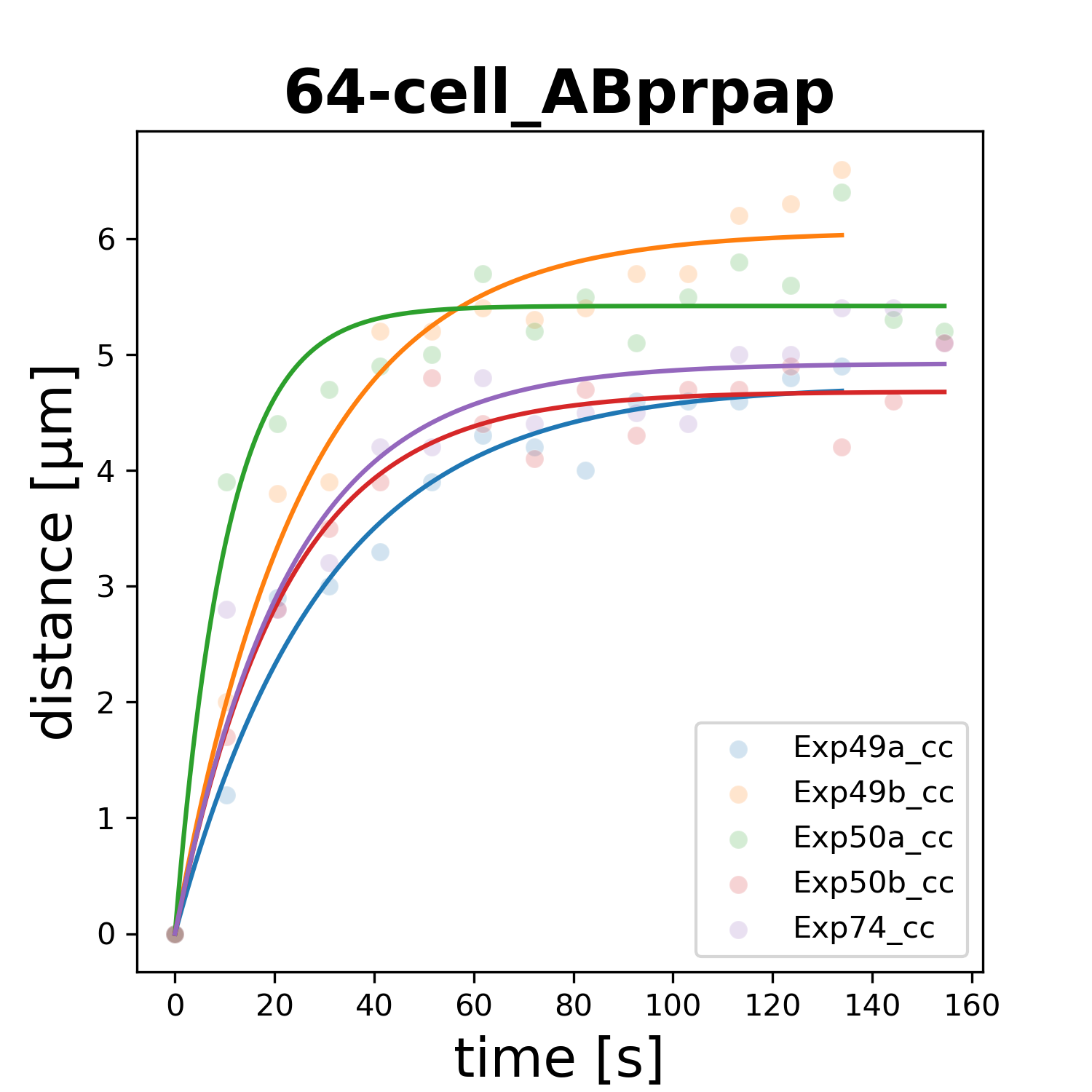

Supplement: Supplement 2 [file media-2.zip › Supplementary Material/ani2(RNAi)_chromosome_to_chromosome_distance/64-cell_ABprpap.png]

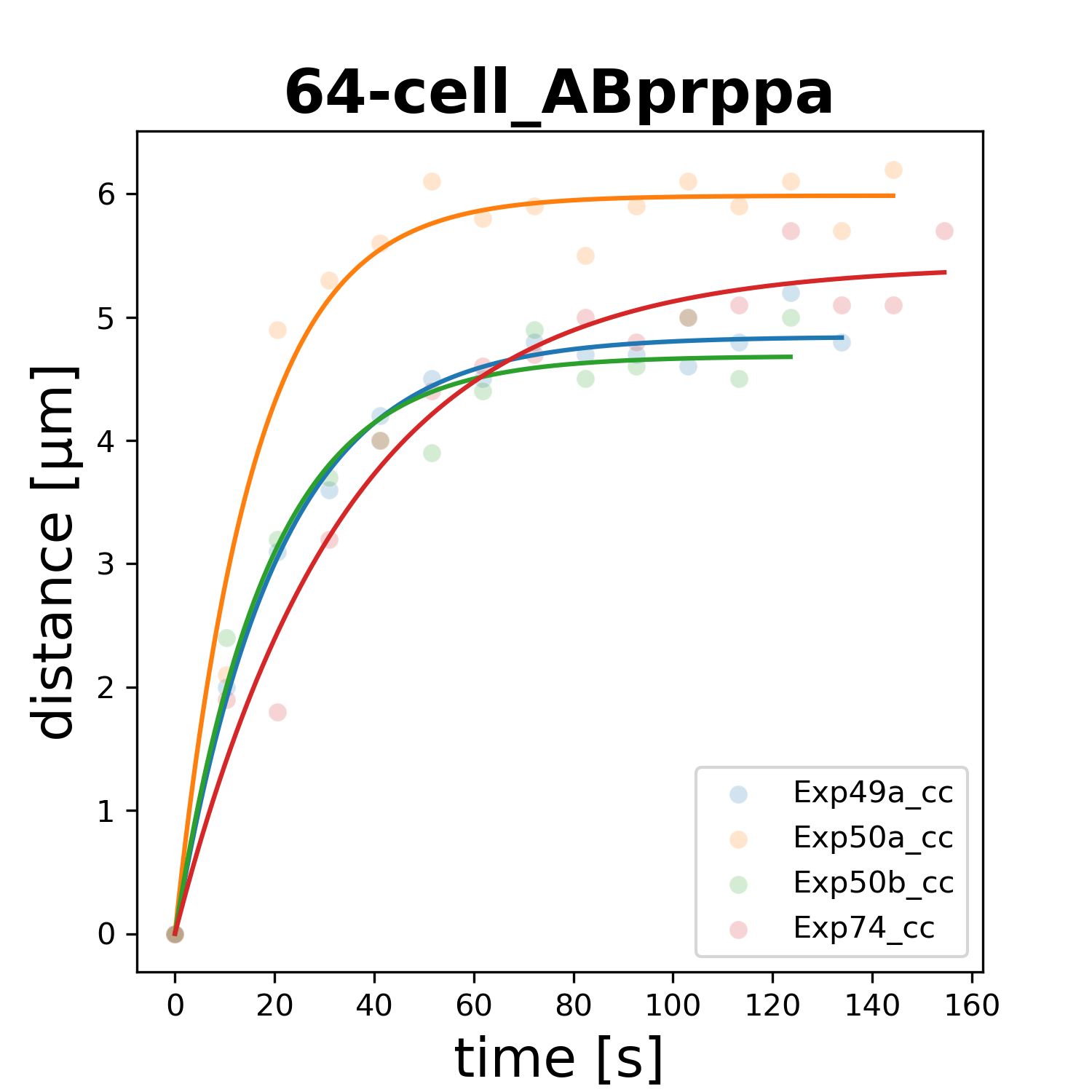

Supplement: Supplement 2 [file media-2.zip › Supplementary Material/ani2(RNAi)_chromosome_to_chromosome_distance/64-cell_ABprppa.png]

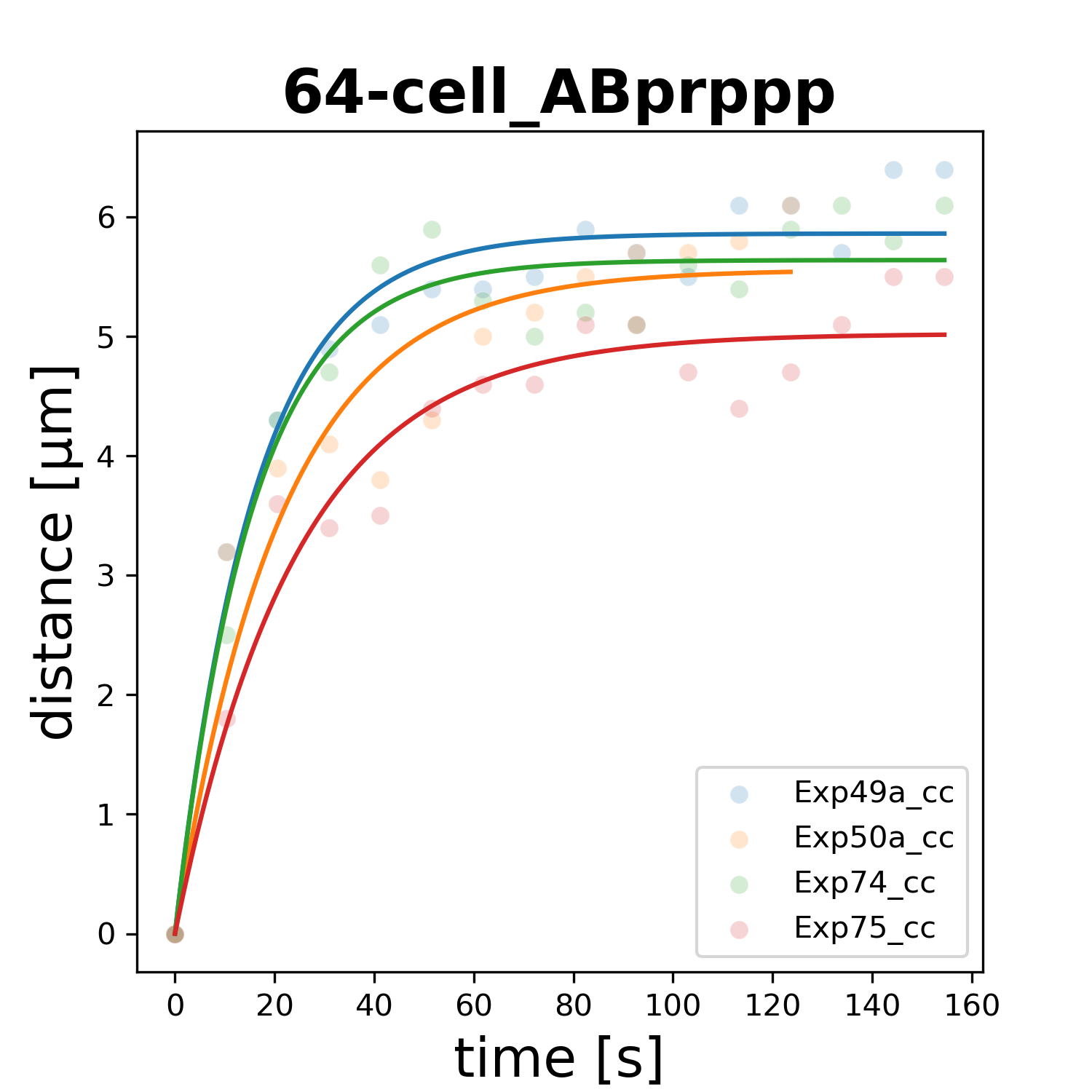

Supplement: Supplement 2 [file media-2.zip › Supplementary Material/ani2(RNAi)_chromosome_to_chromosome_distance/64-cell_ABprppp.png]

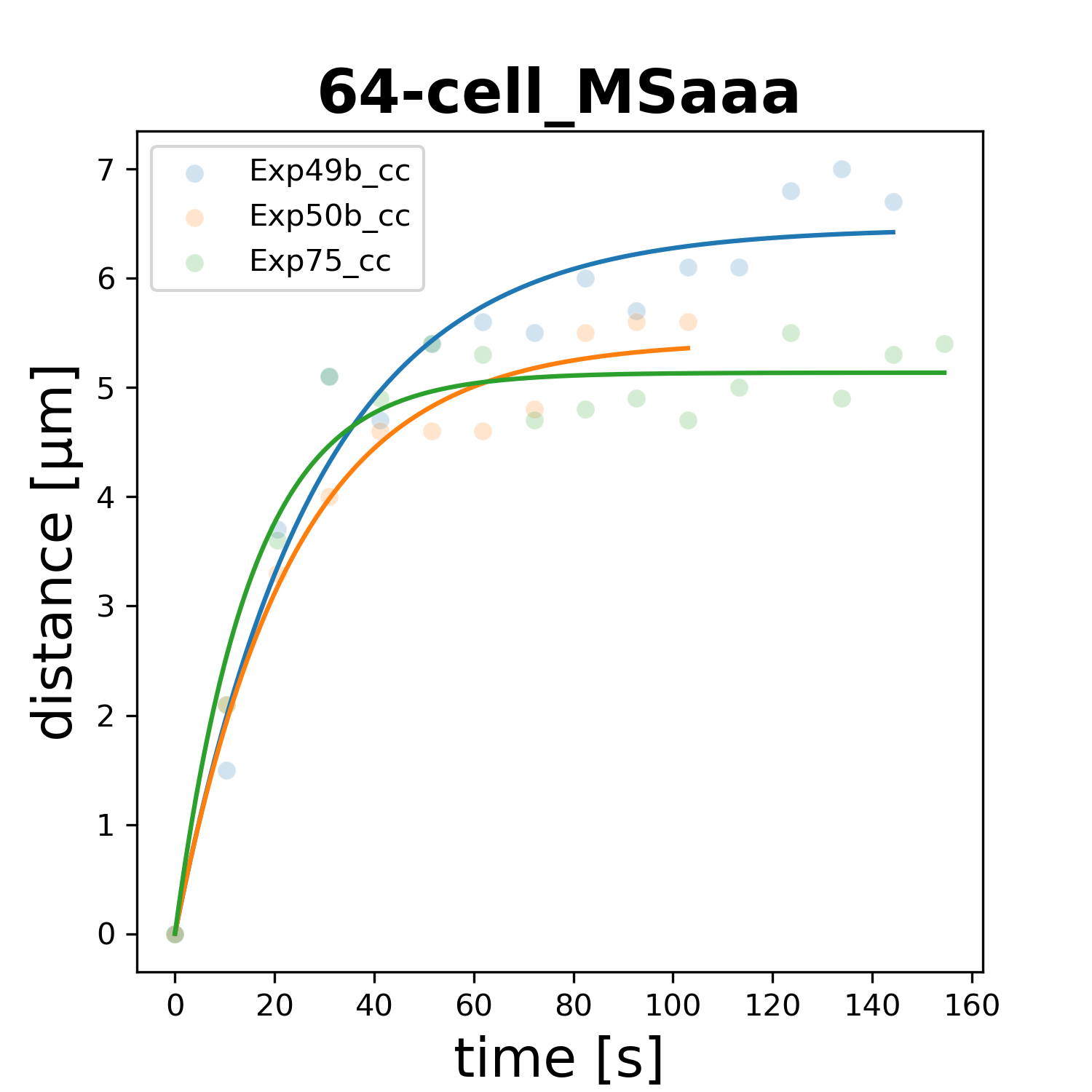

Supplement: Supplement 2 [file media-2.zip › Supplementary Material/ani2(RNAi)_chromosome_to_chromosome_distance/64-cell_MSaaa.png]

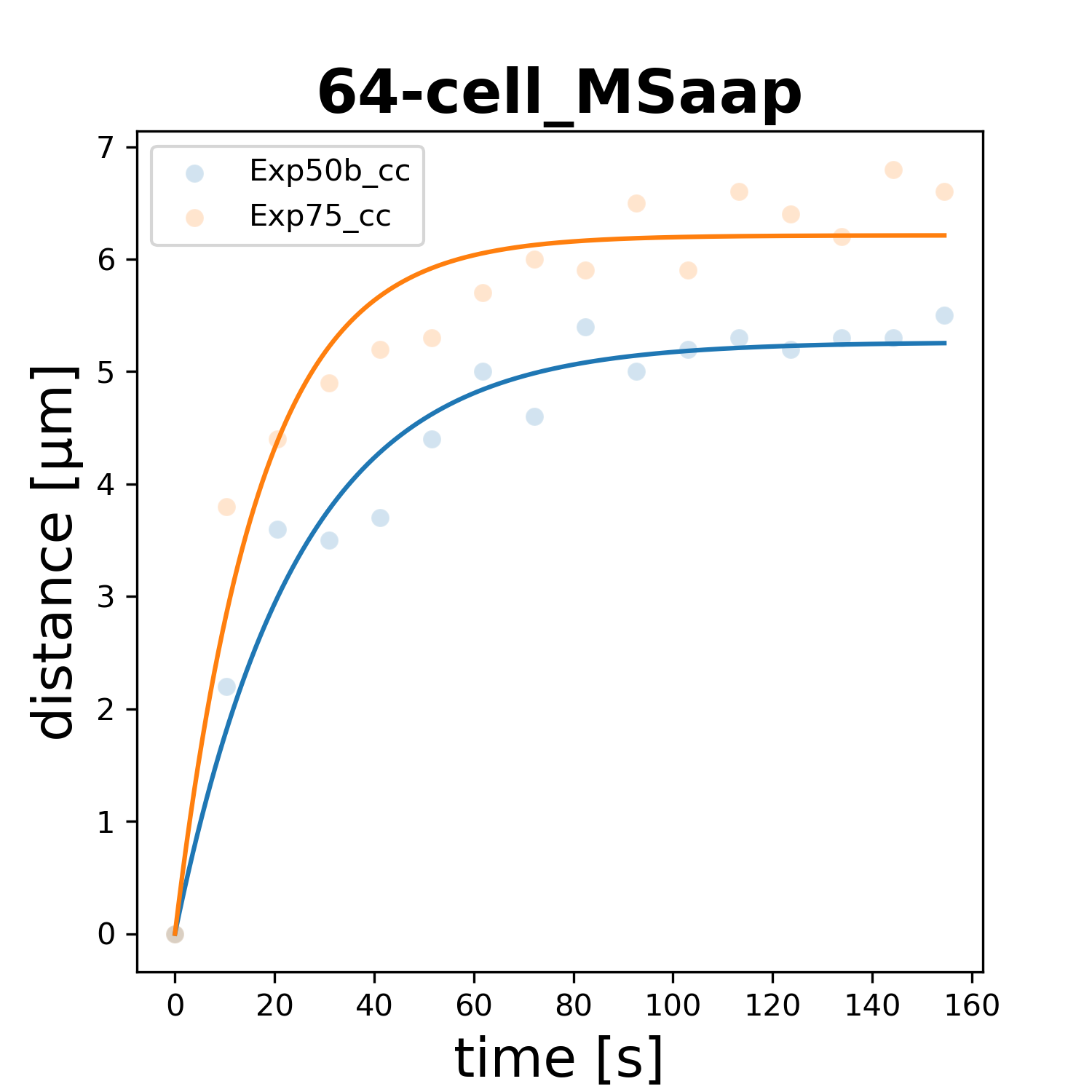

Supplement: Supplement 2 [file media-2.zip › Supplementary Material/ani2(RNAi)_chromosome_to_chromosome_distance/64-cell_MSaap.png]

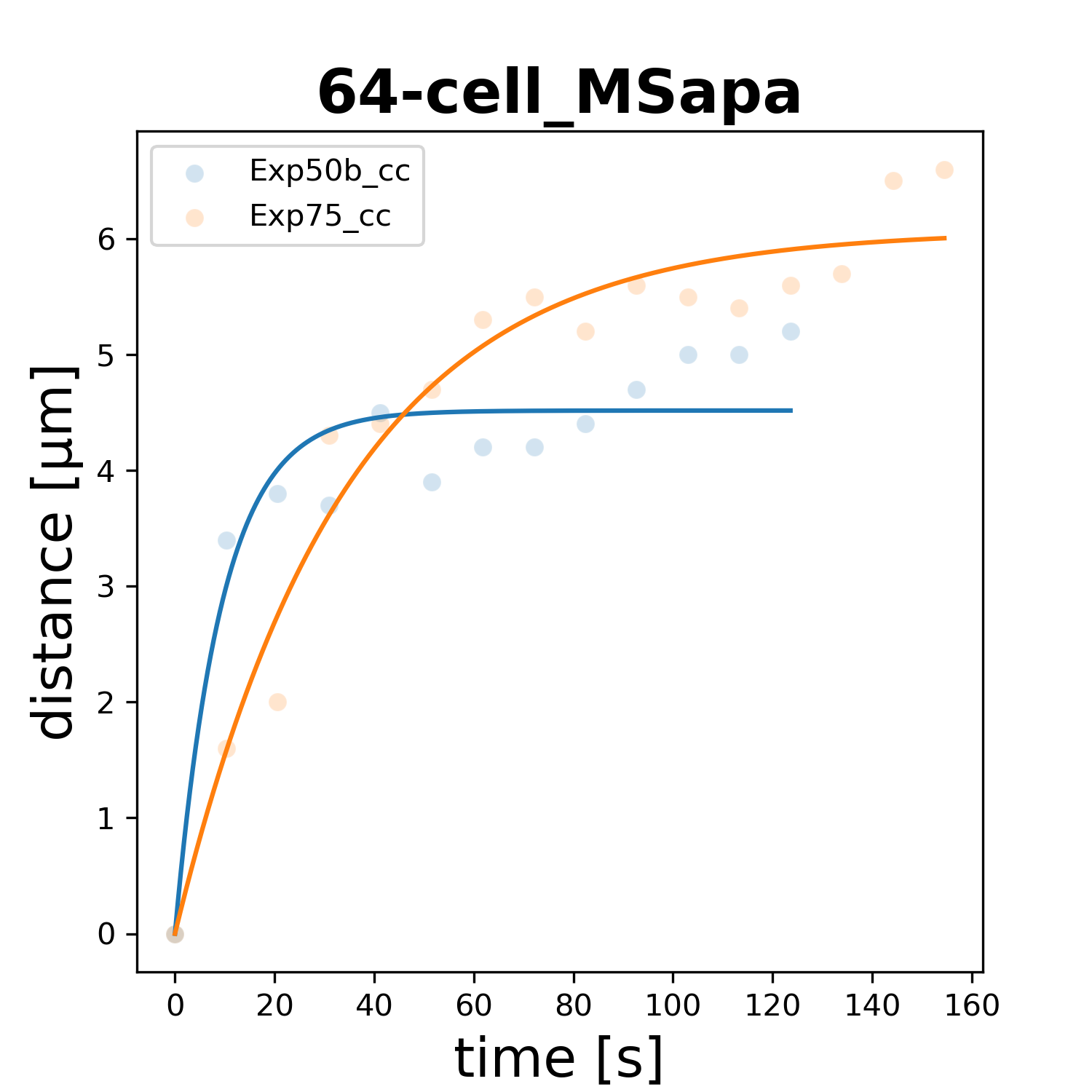

Supplement: Supplement 2 [file media-2.zip › Supplementary Material/ani2(RNAi)_chromosome_to_chromosome_distance/64-cell_MSapa.png]

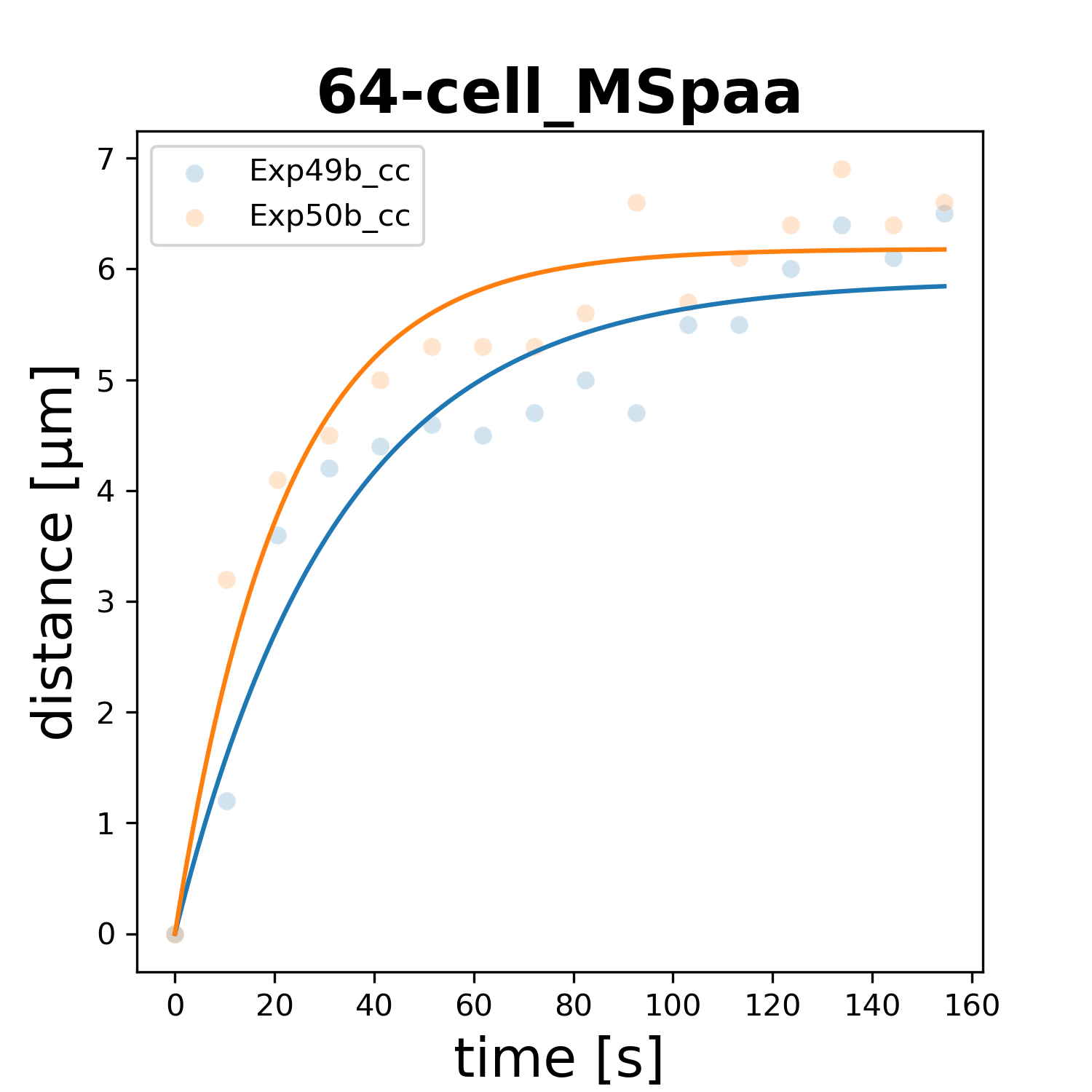

Supplement: Supplement 2 [file media-2.zip › Supplementary Material/ani2(RNAi)_chromosome_to_chromosome_distance/64-cell_MSpaa.png]

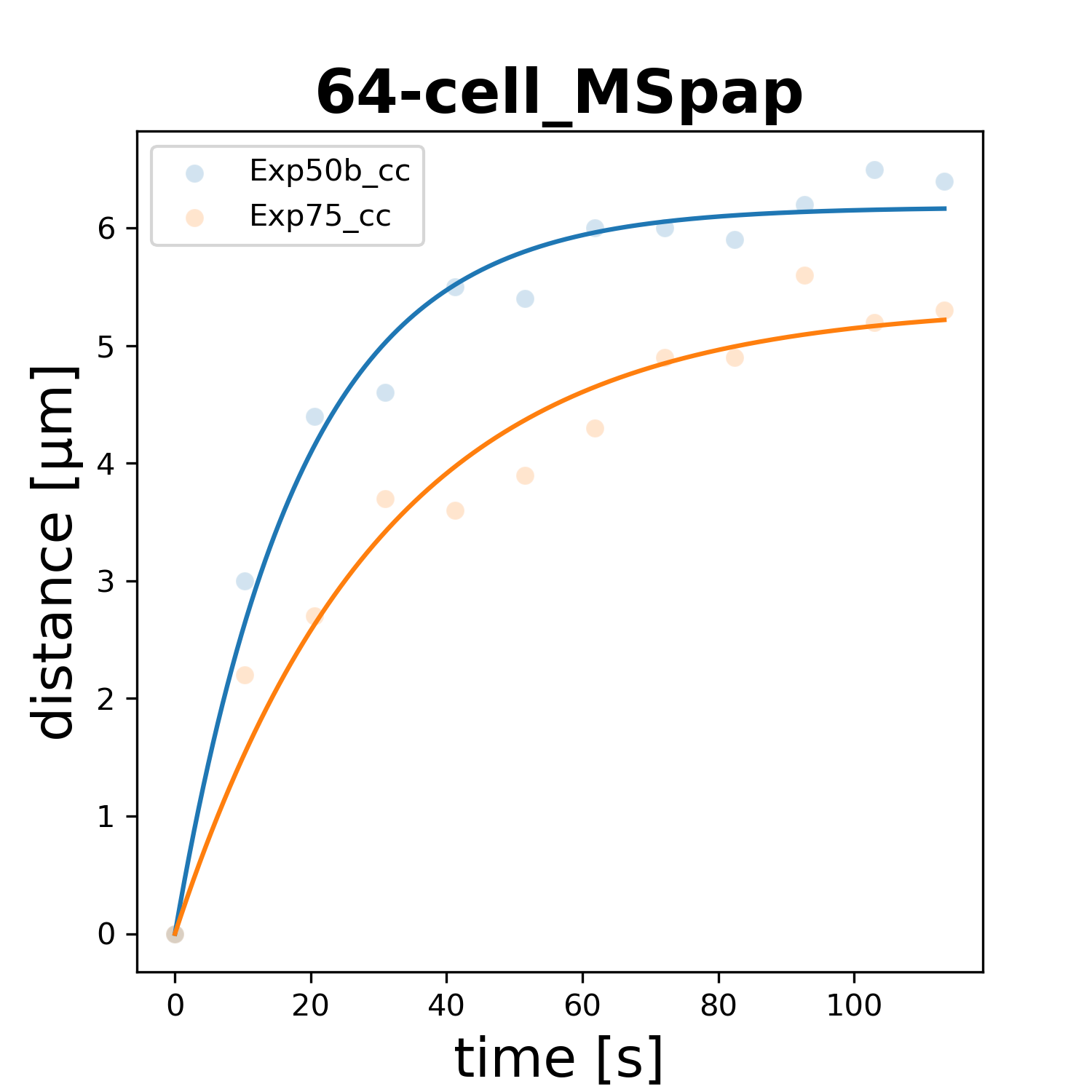

Supplement: Supplement 2 [file media-2.zip › Supplementary Material/ani2(RNAi)_chromosome_to_chromosome_distance/64-cell_MSpap.png]

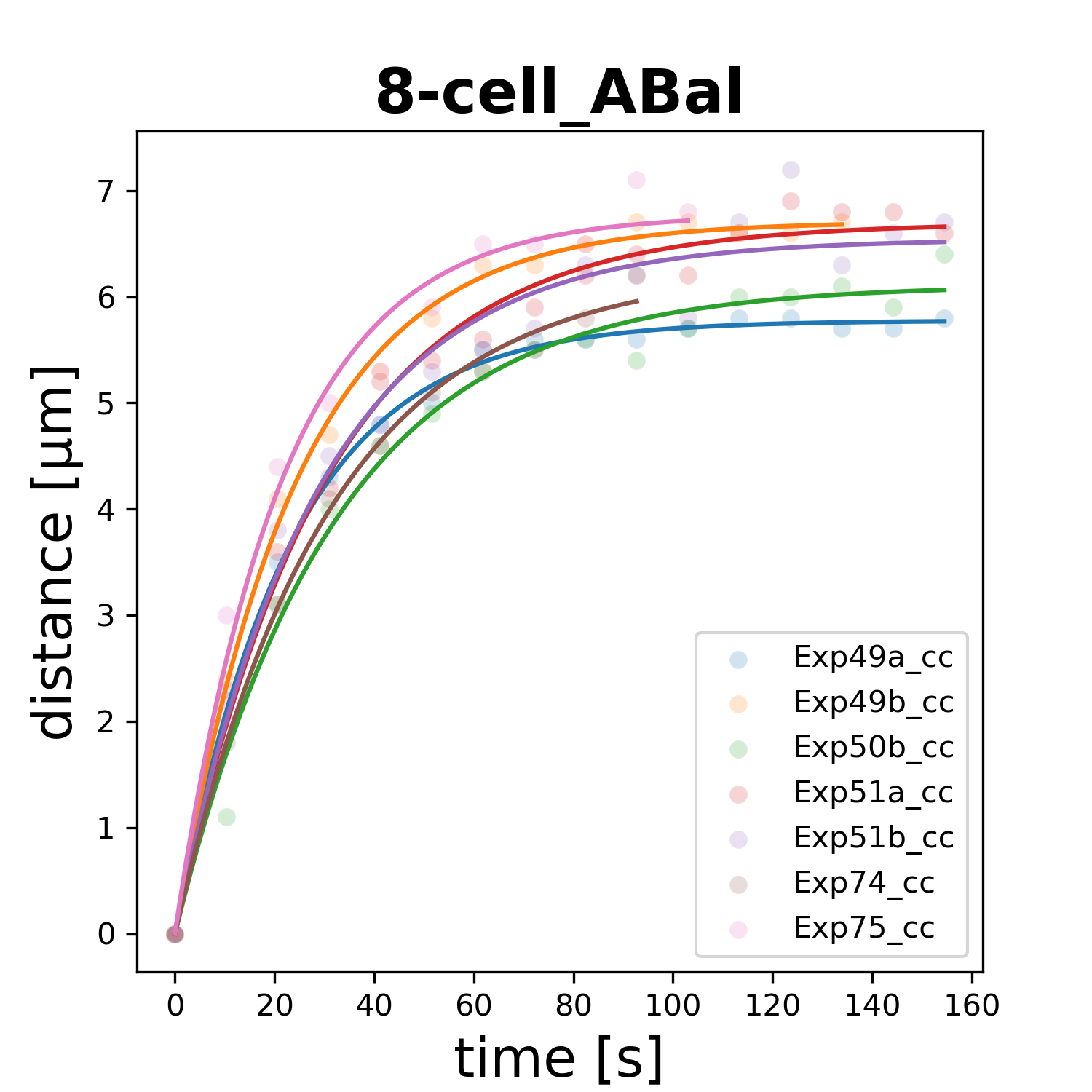

Supplement: Supplement 2 [file media-2.zip › Supplementary Material/ani2(RNAi)_chromosome_to_chromosome_distance/8-cell_ABal.png]

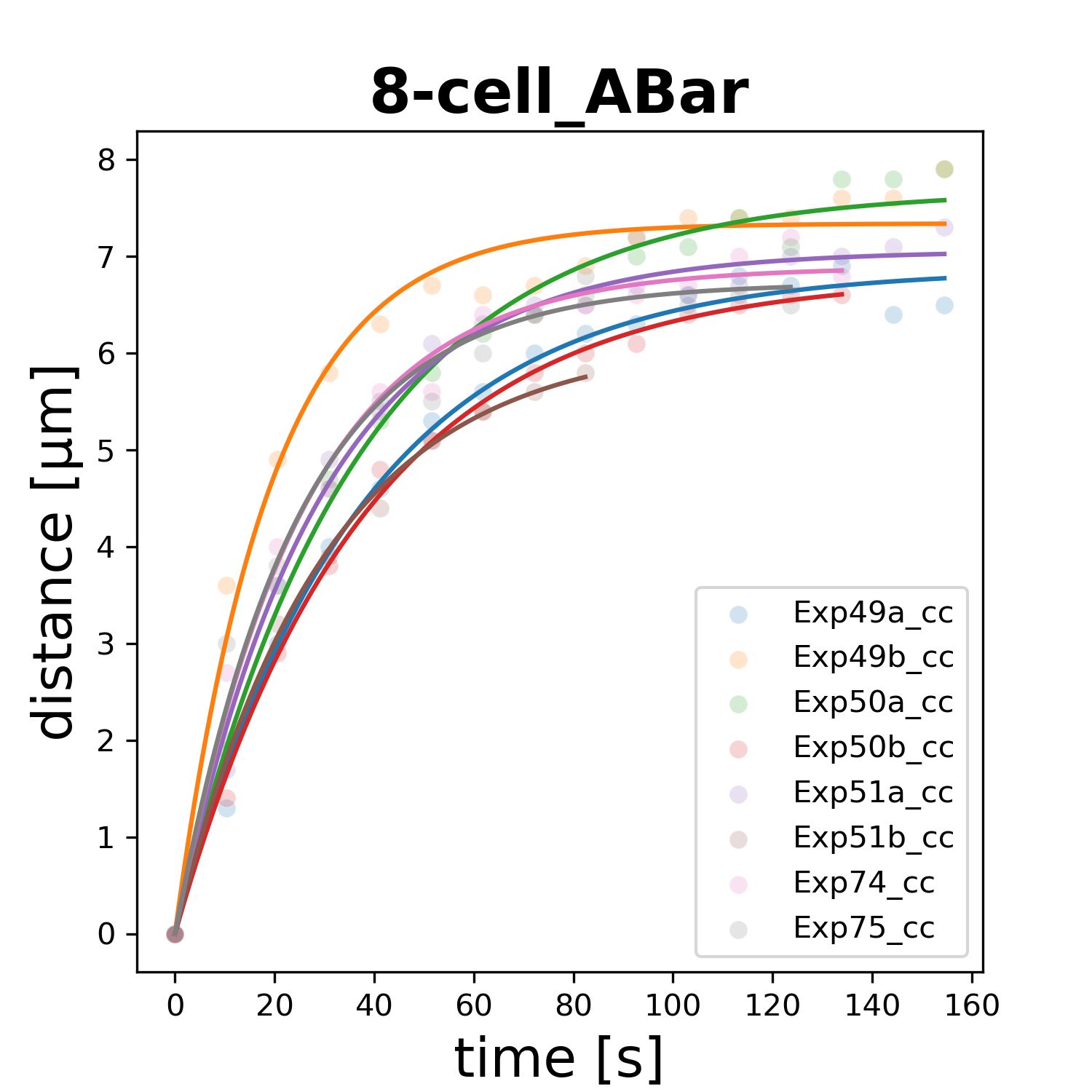

Supplement: Supplement 2 [file media-2.zip › Supplementary Material/ani2(RNAi)_chromosome_to_chromosome_distance/8-cell_ABar.png]

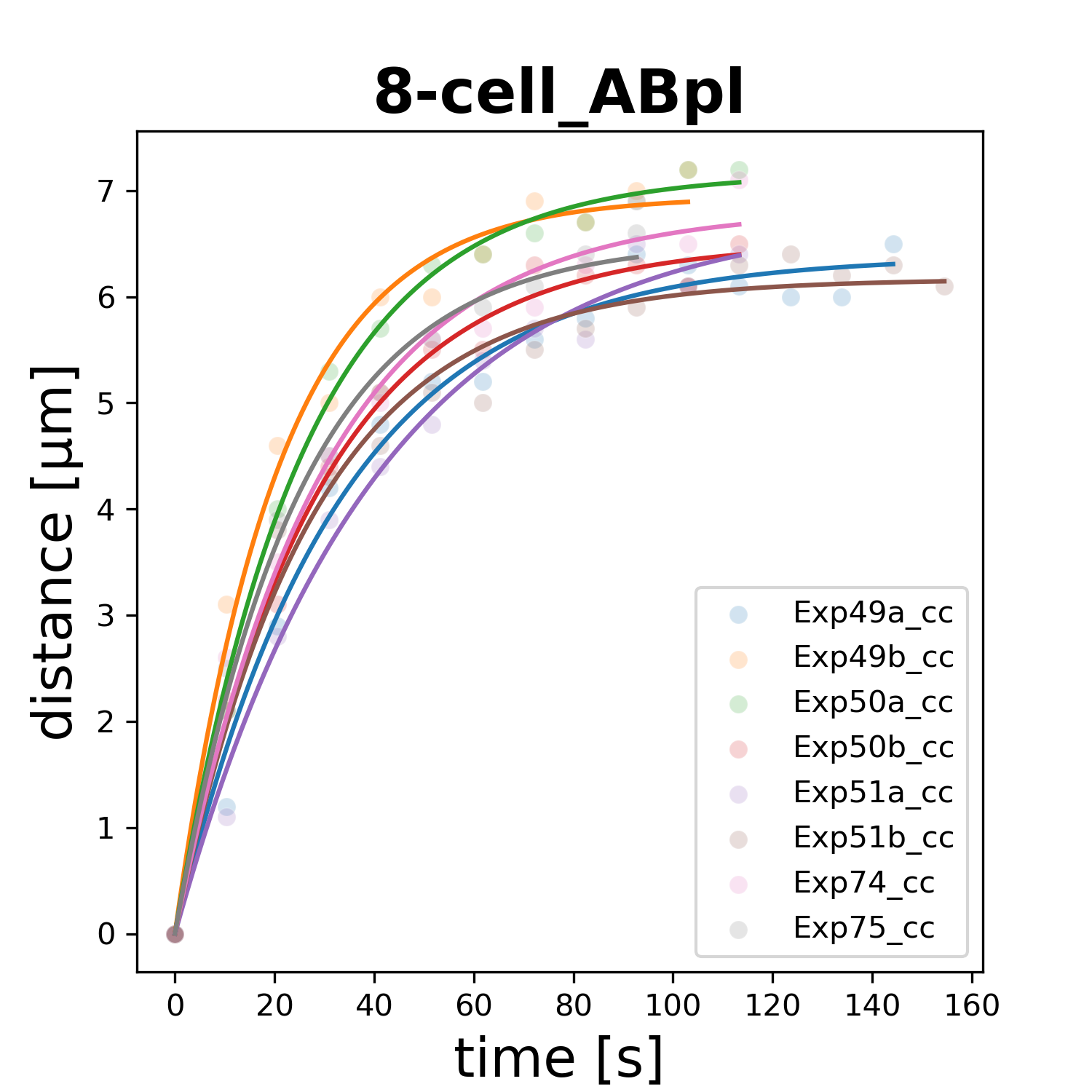

Supplement: Supplement 2 [file media-2.zip › Supplementary Material/ani2(RNAi)_chromosome_to_chromosome_distance/8-cell_ABpl.png]

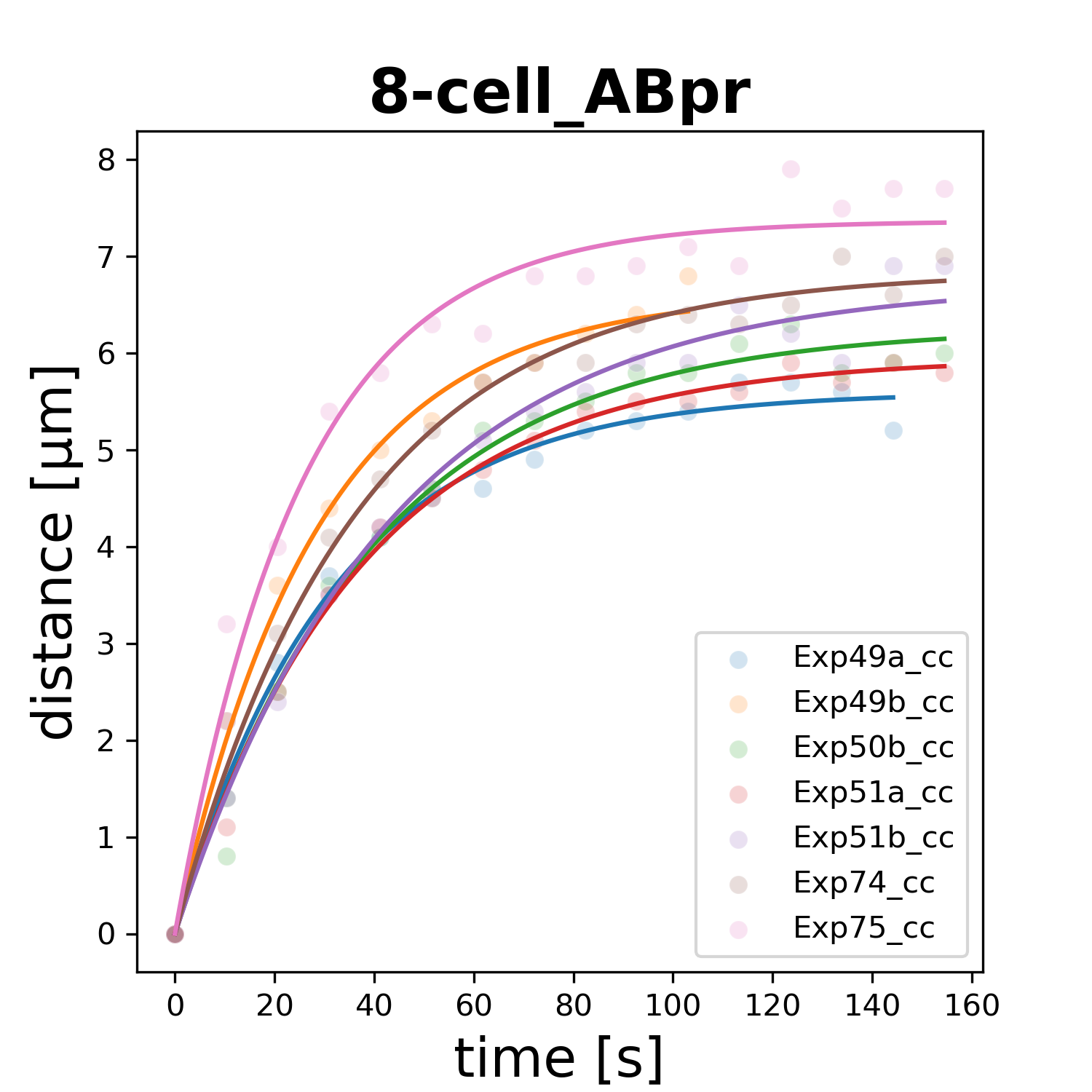

Supplement: Supplement 2 [file media-2.zip › Supplementary Material/ani2(RNAi)_chromosome_to_chromosome_distance/8-cell_ABpr.png]

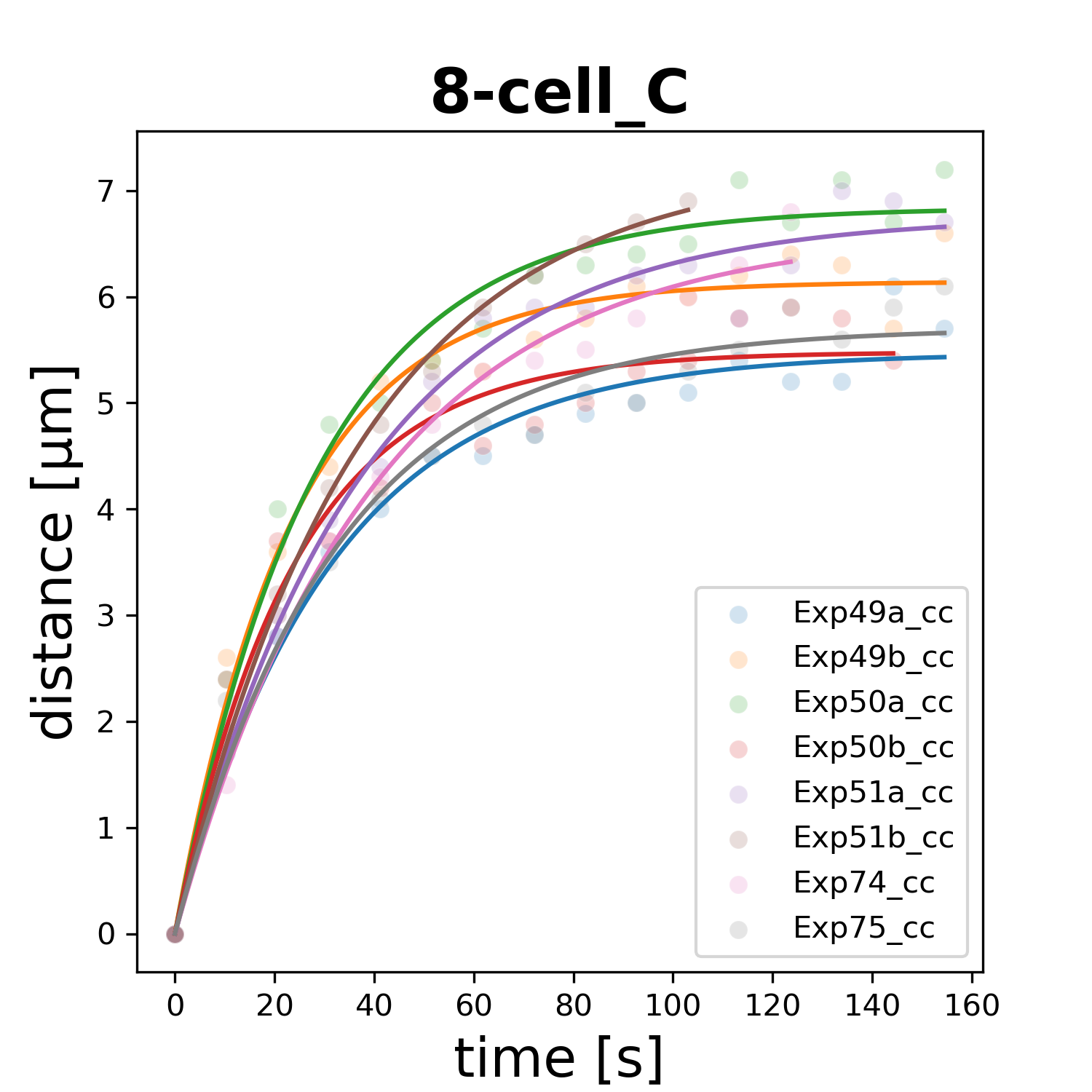

Supplement: Supplement 2 [file media-2.zip › Supplementary Material/ani2(RNAi)_chromosome_to_chromosome_distance/8-cell_C.png]

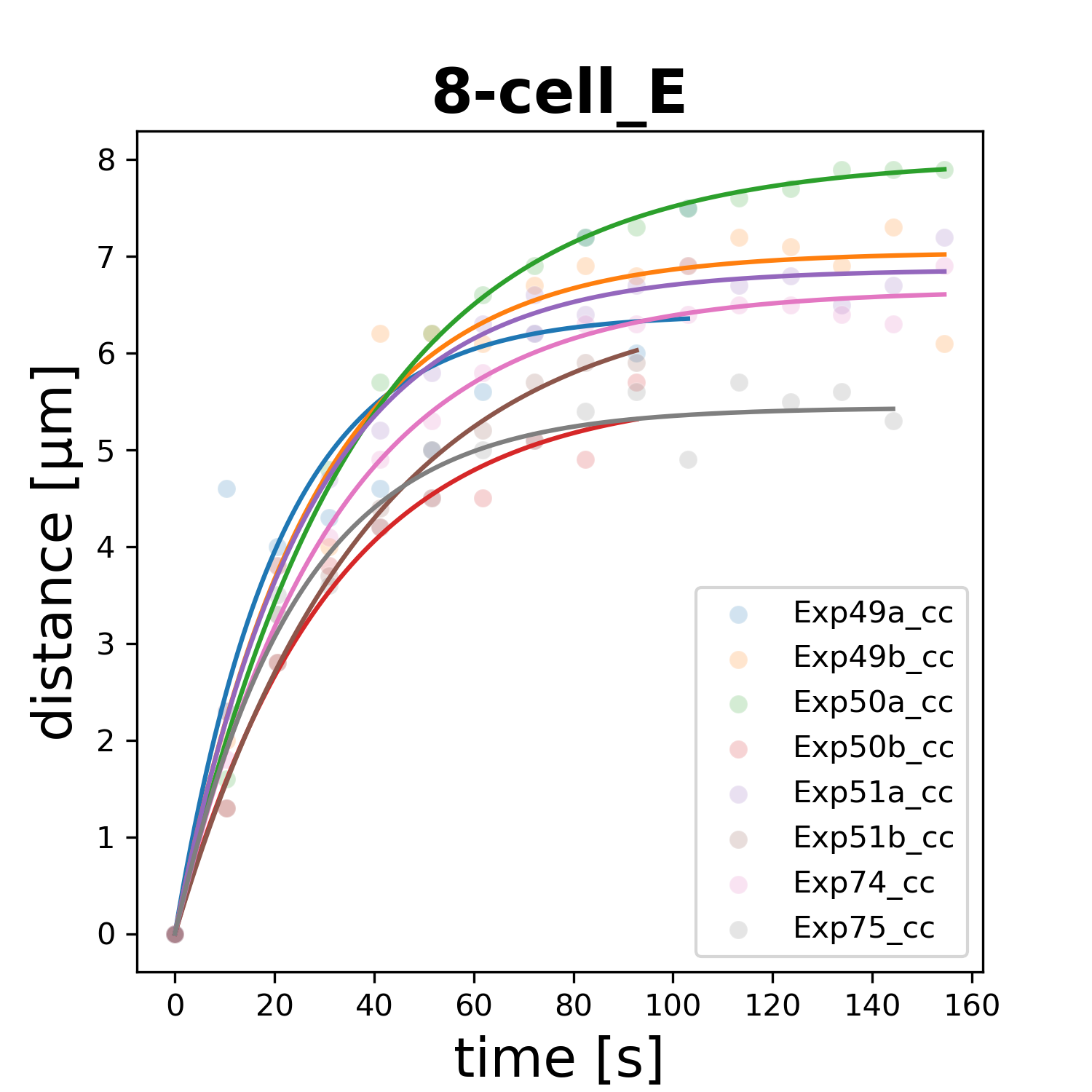

Supplement: Supplement 2 [file media-2.zip › Supplementary Material/ani2(RNAi)_chromosome_to_chromosome_distance/8-cell_E.png]

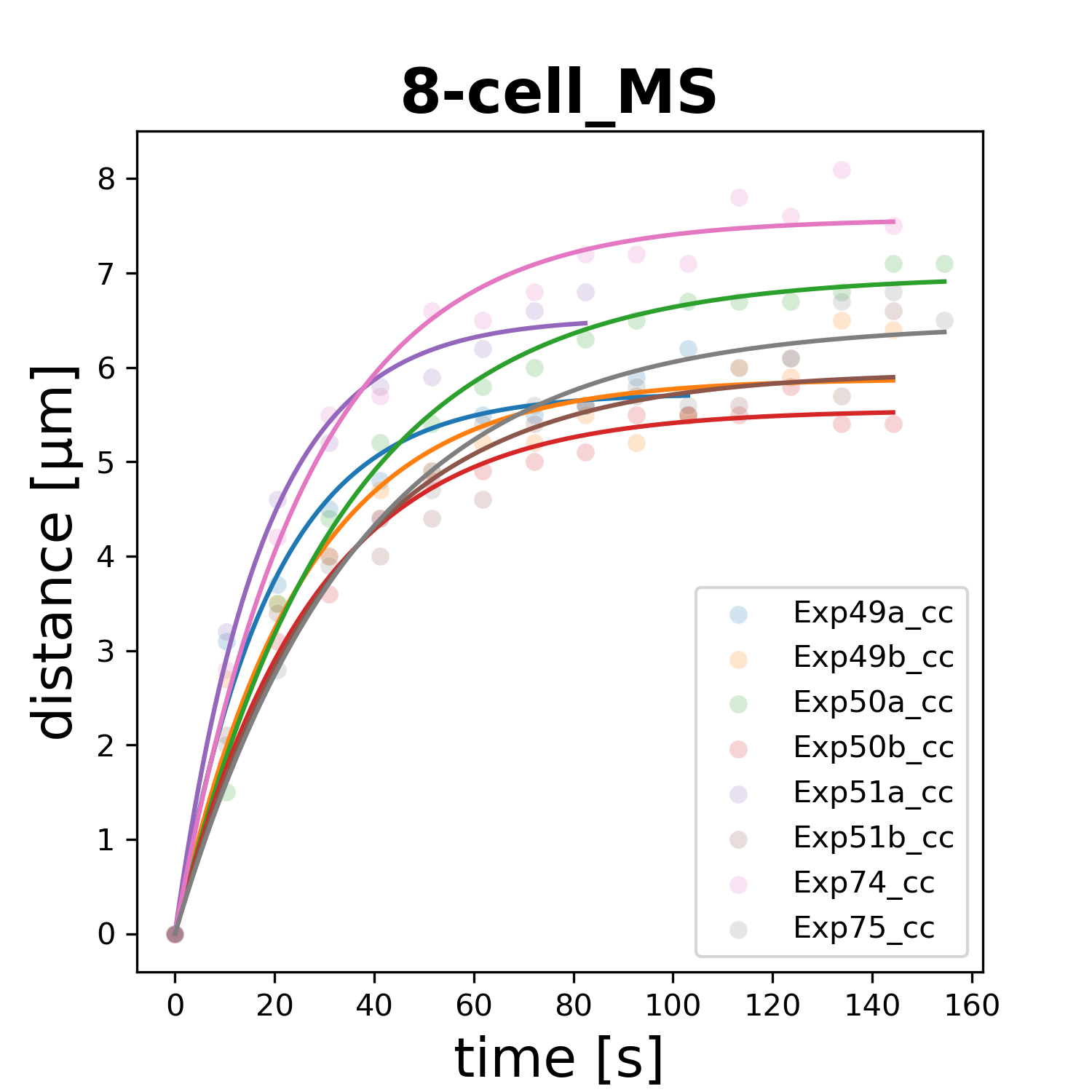

Supplement: Supplement 2 [file media-2.zip › Supplementary Material/ani2(RNAi)_chromosome_to_chromosome_distance/8-cell_MS.png]

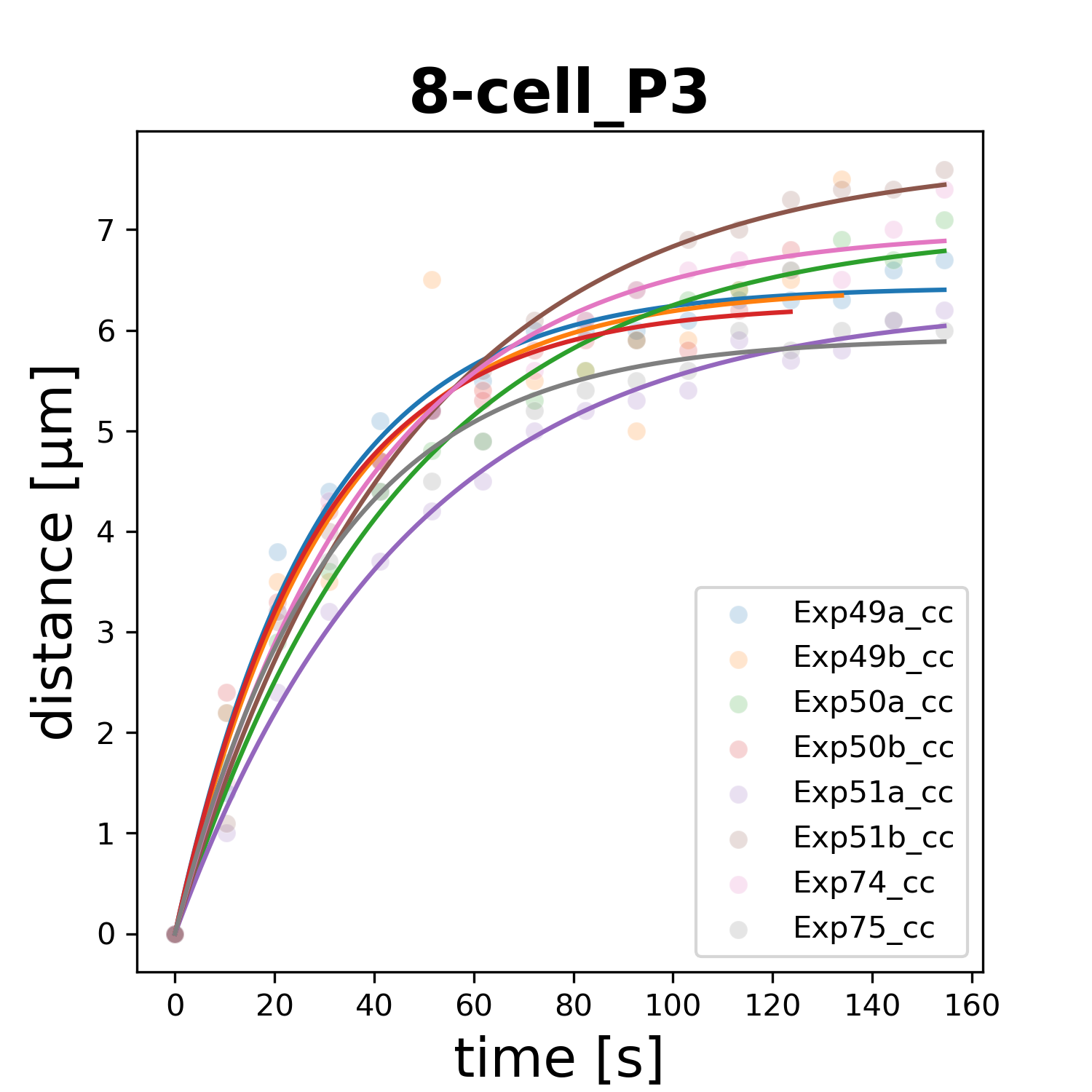

Supplement: Supplement 2 [file media-2.zip › Supplementary Material/ani2(RNAi)_chromosome_to_chromosome_distance/8-cell_P3.png]

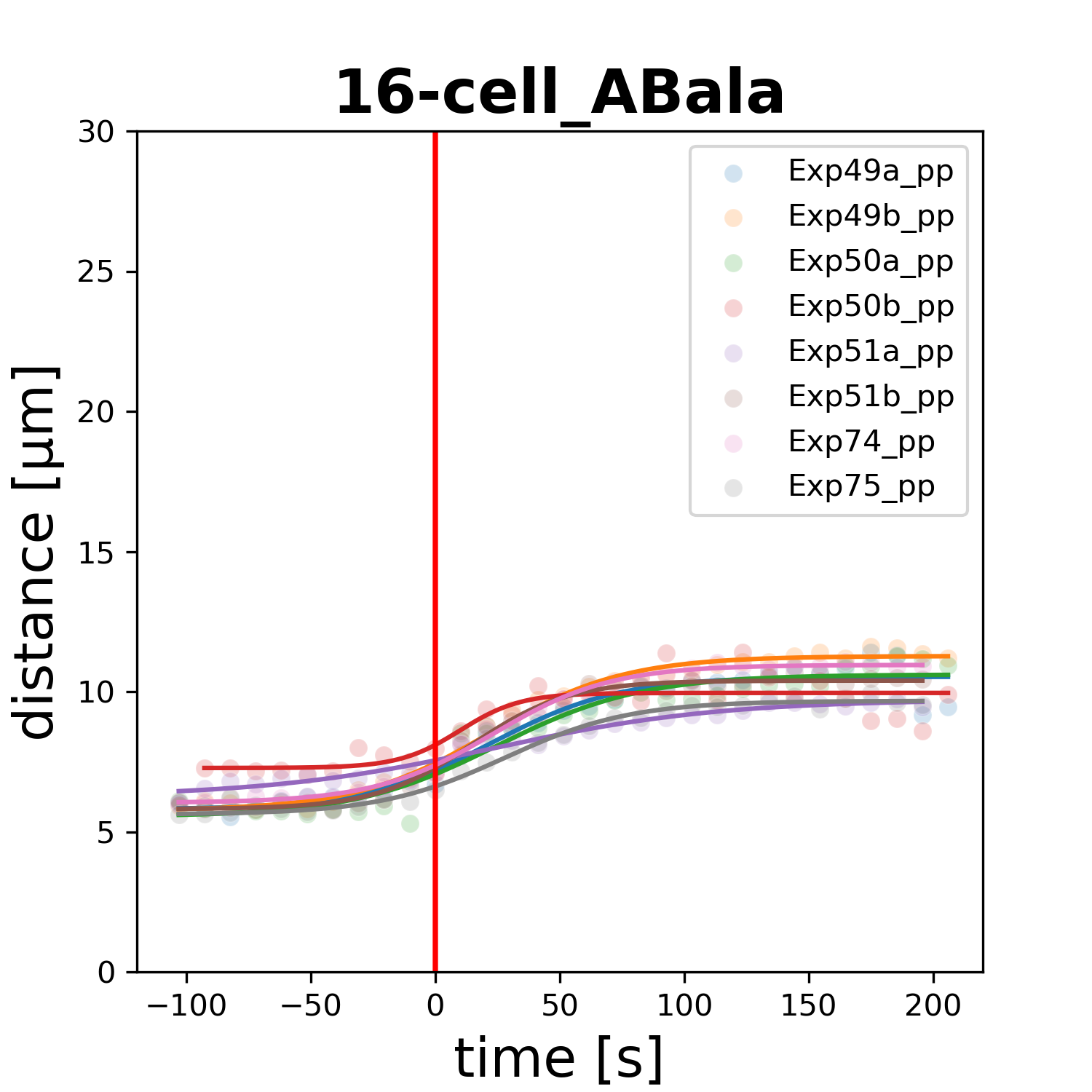

Supplement: Supplement 2 [file media-2.zip › Supplementary Material/ani2(RNAi)_pole_to_pole_distance/16-cell_ABala.png]

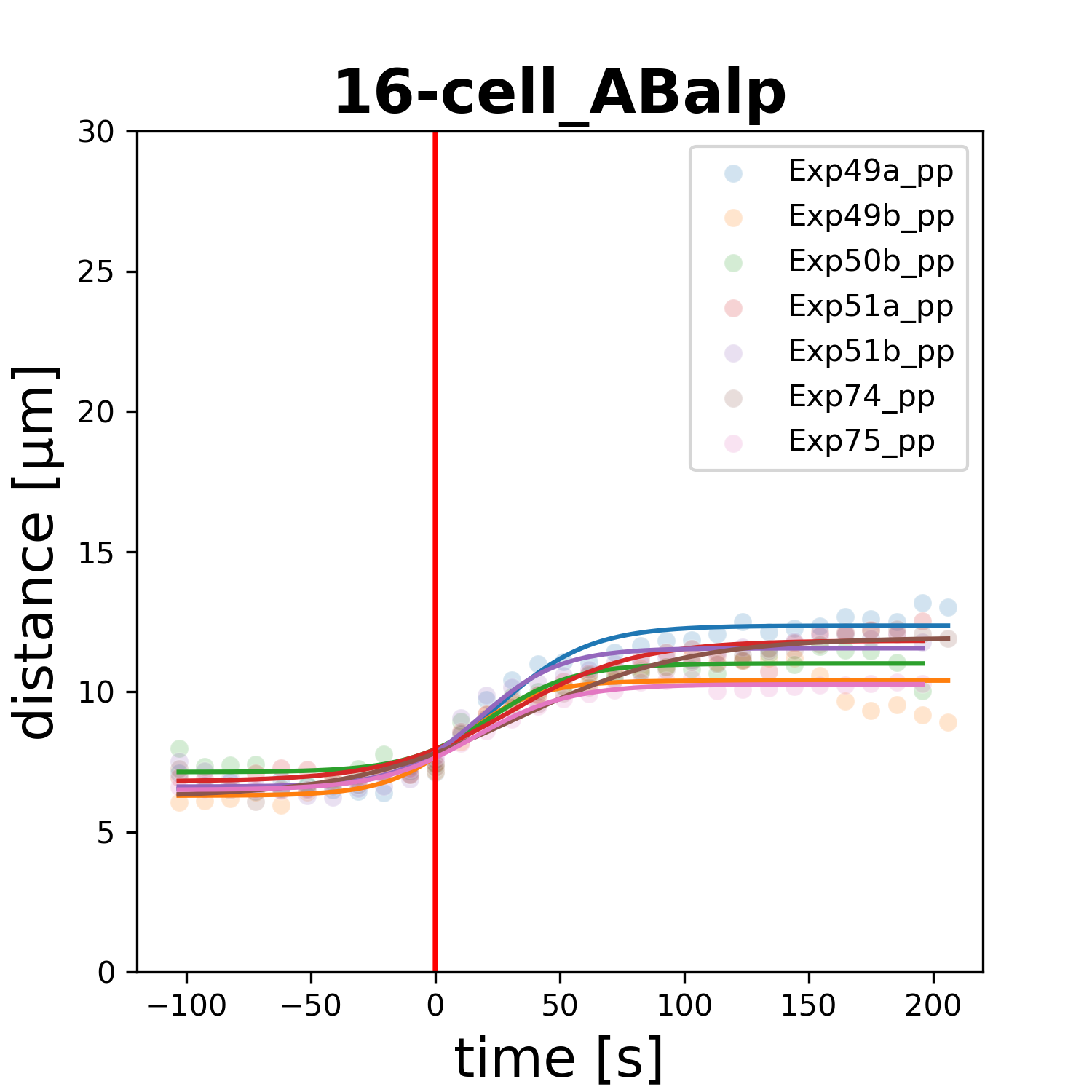

Supplement: Supplement 2 [file media-2.zip › Supplementary Material/ani2(RNAi)_pole_to_pole_distance/16-cell_ABalp.png]

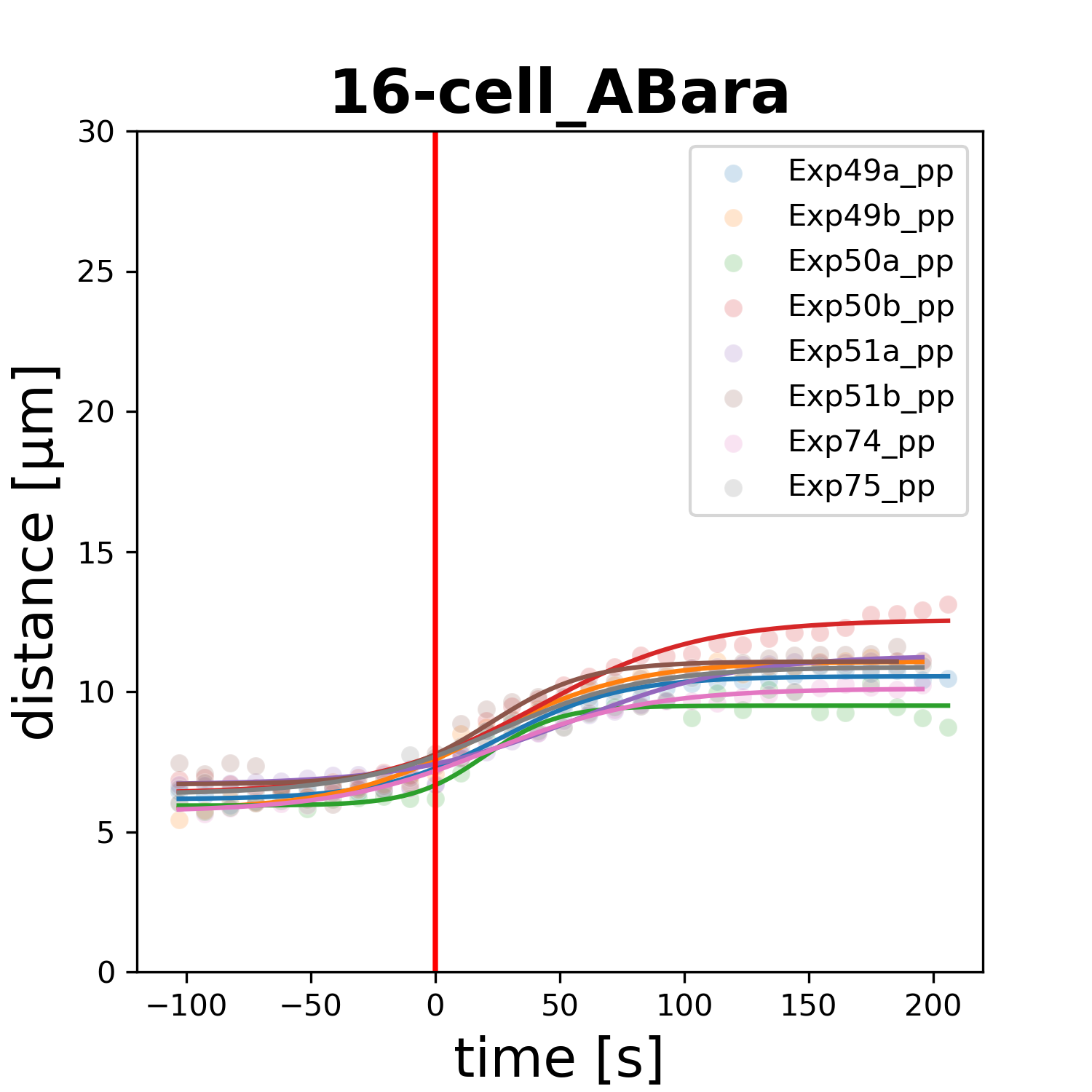

Supplement: Supplement 2 [file media-2.zip › Supplementary Material/ani2(RNAi)_pole_to_pole_distance/16-cell_ABara.png]

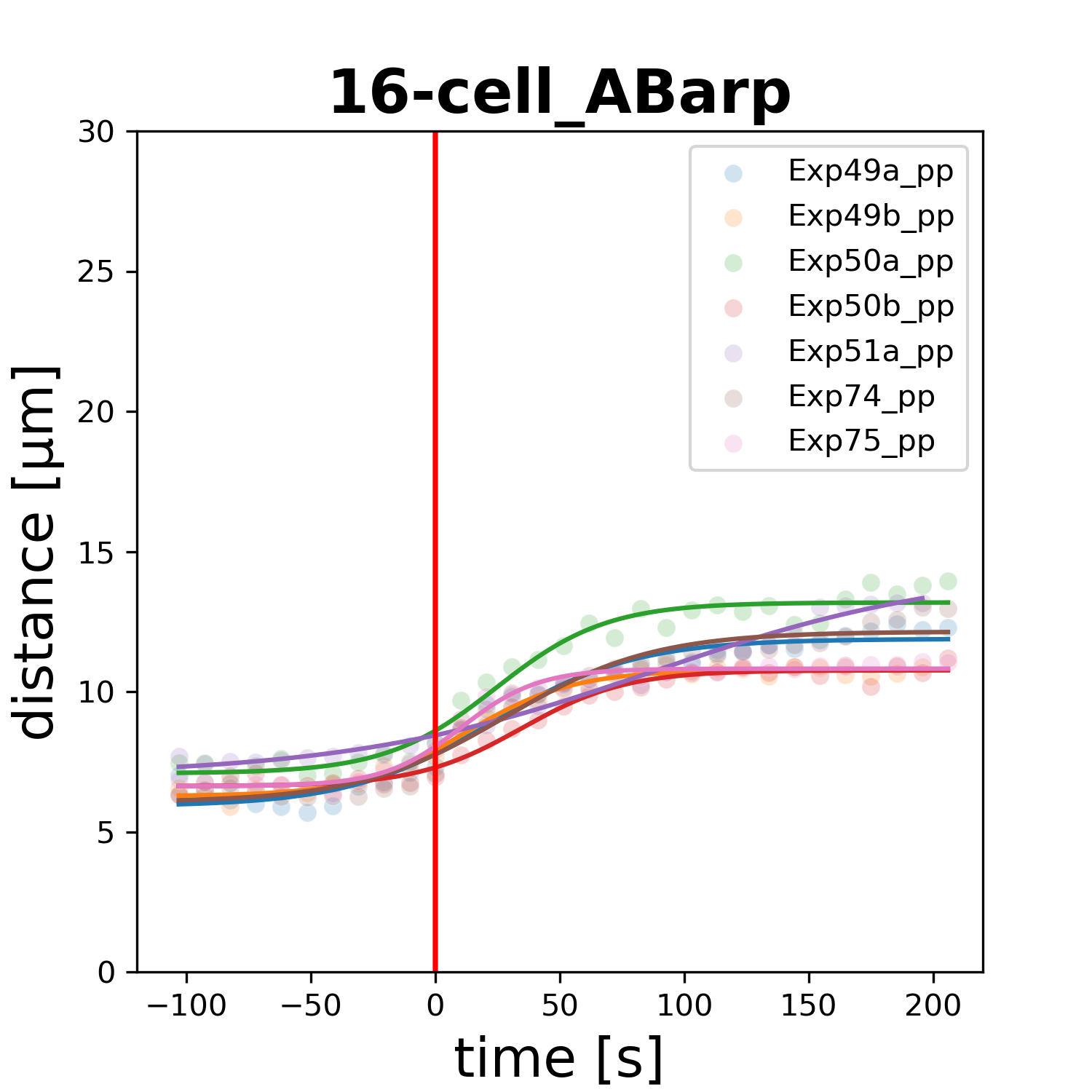

Supplement: Supplement 2 [file media-2.zip › Supplementary Material/ani2(RNAi)_pole_to_pole_distance/16-cell_ABarp.png]

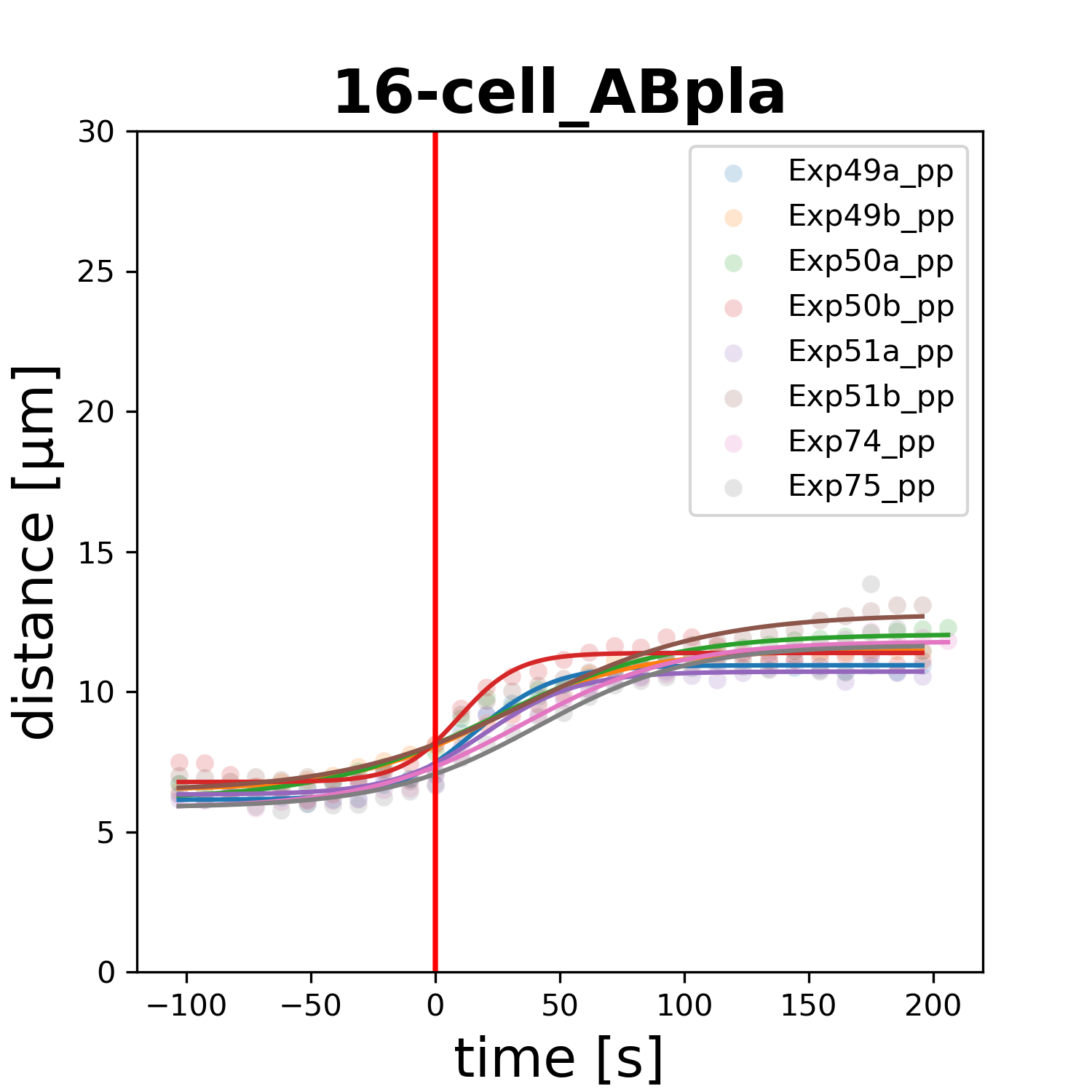

Supplement: Supplement 2 [file media-2.zip › Supplementary Material/ani2(RNAi)_pole_to_pole_distance/16-cell_ABpla.png]
